# Characterisation of a novel SCC*mec* VI element harbouring *fusC* in an emerging *Staphylococcus aureus* strain from the Arabian Gulf region

**DOI:** 10.1371/journal.pone.0223985

**Published:** 2019-11-05

**Authors:** Abiola Senok, Peter Slickers, Helmut Hotzel, Samar Boswihi, Sascha D. Braun, Darius Gawlik, Elke Müller, Anju Nabi, Rania Nassar, Hedda Nitschke, Annett Reissig, Antje Ruppelt-Lorz, Joseph Mafofo, Ali M. Somily, Edet Udo, Ralf Ehricht, Stefan Monecke

**Affiliations:** 1 College of Medicine, Mohammed Bin Rashid University of Medicine and Health Sciences, Dubai, United Arab Emirates; 2 InfectoGnostics Research Campus Jena, Jena, Germany; 3 Abbott (Alere Technologies GmbH), Jena, Germany; 4 Friedrich-Loeffler-Institut (Federal Research Institute for Animal Health), Institute of Bacterial Infections and Zoonoses, Jena, Germany; 5 Department of Microbiology, Faculty of Medicine, Kuwait University, Jabriya, Kuwait; 6 Leibniz Institute of Photonic Technology (IPHT), Jena, Germany; 7 PTC - Phage Technology Center GmbH, Bönen, Germany; 8 Microbiology & Infection Control Unit, Pathology Department, Rashid Hospital, Dubai Health Authority, Dubai, United Arab Emirates; 9 Department of Laboratory Medicine, Hospital Dresden-Neustadt, Dresden, Germany; 10 Institute for Medical Microbiology and Hygiene, Medical Faculty "Carl Gustav Carus", Technische Universität Dresden, Dresden, Germany; 11 Agiomix FZ-LLC, Dubai Science Park Warehouse Complex, Dubai, United Arab Emirates; 12 Department of Pathology and Laboratory Medicine, College of Medicine, King Khalid University Hospital and King Saud University, Riyadh, Saudi Arabia; Universidade Nova de Lisboa, PORTUGAL

## Abstract

Fusidic acid is a steroid antibiotic known since the 1960s. It is frequently used in topical preparations, *i*.*e*., ointments, for the treatment of skin and soft tissue infections caused by *Staphylococcus aureus*. There is an increasing number of methicillin-resistant *S*. *aureus* (MRSA) strains that harbour plasmid-borne *fusB/far1* or *fusC* that is localised on SCC elements. In this study we examined a series of related CC30-MRSA isolates from the Arabian Gulf countries that presented with SCC*mec* elements and *fusC*, including a variant that—to the best of our knowledge—has not yet formally been described. It consisted of a class B *mec* complex and *ccrA/B-4* genes. The fusidic acid resistance gene *fusC* was present, but contrary to the previously sequenced element of HDE288, it was not accompanied by *tirS*. This element was identified in CC30 MRSA from Kuwait, Saudi Arabia and the United Arab Emirates that usually also harbour the Panton-Valentin leukocidin (PVL) genes. It was also identified in CC8 and ST834 isolates. In addition, further CC30 MRSA strains with other SCC*mec* VI elements harbouring *fusC* were found to circulate in the Arabian Gulf region. It can be assumed that MRSA strains with SCC*mec* elements that include *fusC* have a selective advantage in both hospital and community settings warranting a review of the use of topical antibiotics and indicating the necessity of reducing over-the-counter sale of antibiotics, including fusidic acid, without prescription.

## Introduction

Within a year after of the introduction of penicillinase-resistant semi-synthetic penicillins such as methicillin, oxacillin and the first/second generation cephalosporins, methicillin-resistant *Staphylococcus aureus* (MRSA) was reported in the United Kingdom [1]. Beta-lactam resistance in MRSA is due to modified penicillin-binding proteins encoded by different *mec* genes, out of which *mecA* is by far the most common and most widespread [2, 3]. The *mecA* gene is located on potentially mobile, large and complex genetic elements, known as SCC*mec* (“staphylococcal cassette chromosome” or “staphylococcal chromosomal cassette” harbouring *mecA*). In addition to *mecA* or *mecC*, SCC*mec* elements include *ccr* recombinase genes, regulatory elements and, variably, additional genes encoding resistance to other antimicrobials, such as aminoglycosides or macrolides, and to heavy metal ions [4–11]. They also might contain the gene *fusC* encoding fusidic acid resistance [12, 13]. Fusidic acid [14, 15] is a steroid antibiotic known since the 1960s. It is frequently used in topical preparations, *i*.*e*., ointments, for the treatment of skin and soft tissue infections caused by *S*. *aureus*. In some countries, intravenous preparations are licensed that are administered in combination with other antimicrobials in order to treat staphylococcal bloodstream or orthopaedic infections. Resistance in staphylococci towards fusidic acid can essentially be attributed to five different genetic causes. One is caused by random mutation under selective pressure in the ubiquitous *fusA* or *efg* gene coding for elongation factor G [16]. Similarly, point mutations in *fusE*, or *rplF*, encoding riboprotein L6 can confer resistance [17, 18]. Another mechanism is related to the presence of the plasmid-borne gene *fusB*, also known as *far1*. Its gene product binds to elongation factor G (*efg*) and thereby protects *efg* from fusidic acid. Acquired resistance due to *fusB/far1* is commonly observed in the community acquired MRSA strain CC80-MRSA-IV that is common in Mediterranean and Middle Eastern countries [19–26]. A similar gene, *fusC* (Q6GD50) is localized on SCC elements. Such an element was first sequenced in a methicillin-susceptible strain, MSSA476 (GenBank BX571857.1) [27] where it is accompanied by *ccrA/B1* genes. However, there are also various SCC*mec* elements that comprise both, *mecA* and *fusC*, together with various combinations of *ccr* genes and other markers [12, 13]. One of these markers is *tirS*, a putative virulence factor mimicking the human Toll/interleukin-1 receptor (TIR) resulting in attenuation of the inflammatory response [28]. Finally, there is a gene, *fusD*, that has been found in various coagulase-negative staphylococci (*S*. *arlettae*, *S*. *cohnii*, *S*. *microti*, *S*. *pettenkoferi* and *S*. *saprophyticus*) [17] but apparently not yet in *S*. *aureus*. A high consumption of fusidic acid at a population level has been shown to confer a clear selective advantage to strains carrying *fusC* and subsequently to their emergence and proliferation as it was well documented for New Zealand [29]. Much less is known on the situation in other geographic regions. However, a high prevalence of *fusB/far1* nd *fusC* and/or a high prevalence of fusidic acid resistance suggest a similar effect in Middle Eastern/Arabian Gulf countries. Indeed, fusidic acid is—or was until recently—available for purchase over-the-counter without prescription there. In the United Arab Emirates (UAE), prescriptions for the purchase of antibiotics became mandatory in late 2017. In this work we examine a series of related MRSA isolates from Arabian Gulf countries that presented with SCC*mec* VI elements and *fusC*. This included a variant that—to the best of our knowledge—has not yet formally been described and therefore it was characterised in detail.

## Material and methods

### Isolates

One CC30 MRSA isolate (RUH-32) obtained in September 2014 from a patient with septic arthritis at the King Khalid University Teaching Hospital in Riyadh, Saudi Arabia, yielded a microarray hybridisation pattern that did not match hybridisation patterns of previously known SCC elements. This isolate was subjected to whole genome sequencing. The BioSample accession number for the isolate is SAMN06925305, the master accession number of the assembled contigs is SGWB00000000.1. This investigation prompted a search for additional CC30 isolates with both, SCC*mec* VI elements and *fusC* yielding one isolate from Dubai, UAE (2018), sixteen from Kuwait (2016/2017) [30]and two from Riyadh, Saudi Arabia (2014 and 2018). All were obtained from hospitalised patients. Finally, two archived non-CC30 isolates were retrospectively found by using an array for SCC*mec* characterisation (see below and [9]) to harbour the same variant of a SCC*mec* VI element as RUH-32. One was a ST834-MRSA isolated in 2011 from a cutaneous abscess of a 3 years old child from Riyadh [31]. The other one was a CC8-MRSA that was isolated in 2017 from a hospitalised patient in Dresden, Saxony, who had a history of travel to, or migration from, the Middle East.

### Microarray-based molecular characterisation

All isolates were characterised using two different microarray-based assays (Alere Technologies GmbH/Abbott, Jena, Germany) designed for *S*. *aureus* typing [32, 33] and for the characterisation of SCC elements [9]. This allowed a rapid detection of species markers, virulence genes, resistance genes and SCC-related markers as well as an assignment to strains and clonal complexes. Details on DNA preparation and hybridisation procedures, as well as on probe and primer sequences and on data analysis have been described previously [9, 32, 33].

### Genome sequencing

Whole-genome sequencing of the RUH-32 isolate was carried out by a commercial service provider using the Illumina HiSeq-2500 platform. Raw reads were deposited in the Short Read Archive under accession SRR5520614. Sequencing reads were assembled *de-novo* using SPAdes version 3.10.1 (http://bioinf.spbau.ru/spades). 51 Contigs were obtained with sizes larger than 200 nt and k-mer coverages greater than 10. Sequences from two reference strains, MRSA18 (European Nucleotide Archive accession number ERR108048) and 20121643 (ERR1595888), were downloaded from the European Nucleotide Archive and assembled with spades. In both cases, the complete SCC elements were found to be located on a single contig. The sequences of the SCC elements were excised from the respective contigs and then annotated.

### Sanger sequencing for closing the gap between the contigs

Inspection of the contigs revealed that two contigs comprised typical SCC genes and that they may be linked by an IS431-like insertion element. Primers were designed for amplifying and capillary electrophoresis (CE) sequencing of the ambiguous linker region. Primer sequences are provided in Table 1. The process included PCR amplification using a thermal cycle program with an initial denaturation temperature of 96°C for 60s followed by 35 cycles of denaturation at 96°C for 15s, annealing at 70°C for 60s and extension at 72°C for 90s. The PCR product was fractionated by gel electrophoresis on a 1.5% agarose, the band with the main amplicon was excised and purified with the QIAamp Gel Extraction Kit (Qiagen, Hilden, Germany) according to the recommendations of the manufacturer. Cycle sequencing was carried out using BigDye Terminator v1.1 Cycle Sequencing Kit (Applied Biosystems, Darmstadt, Germany) on a ABI PRISM 3130 (Applied Biosystems). The first sequence obtained was then further extended by primer walking to a final size of 1133 nt (submitted to GenBank as “linker_RUH-32”). The final linker sequence (GenBank accession number MK991790) is overlapping with contig SGWB01000020.1 by 303 nt, and with contig SGWB01000002.1 by 490 nt. These two contigs and the linker sequence were joined to a single contiguous sequence. For detailed sequence analysis, the SCC element and its flanking genes were extracted and deposited as a separate sequence entry in GenBank (accession number is MK991791). The SCC element was annotated by comparison to a database of genes which have previously been found in related SCC elements.

**Table 1:** Primers for amplification and conventional sequencing.

Primer designation	Aim	Sequence (5´-3´)
IS431syntheny_05	A, S	TCT ATG GTA GTG AAA TCA AAC GGG AG
IS431syntheny_06	A, S	TCG TAT TCT TCG ACT GAT AAT TGC TCT C
IS431-seq-01	S	ATT GAA GAG ATT ATT TTC GG
IS431-seq-02	S	CTA AGA TAT ACA TTG AGT TAT CG
IS431-seq-03	S	CTT TGC TGT ATT GAT ACT TTG
IS431-seq-04	S	CAA TTT TGT ATC AAA TTT GG

A—Amplification, S—Sequencing

## Results

### A novel variant of a SCC*mec* VI element harbouring *fusC*

**Fig 1:**
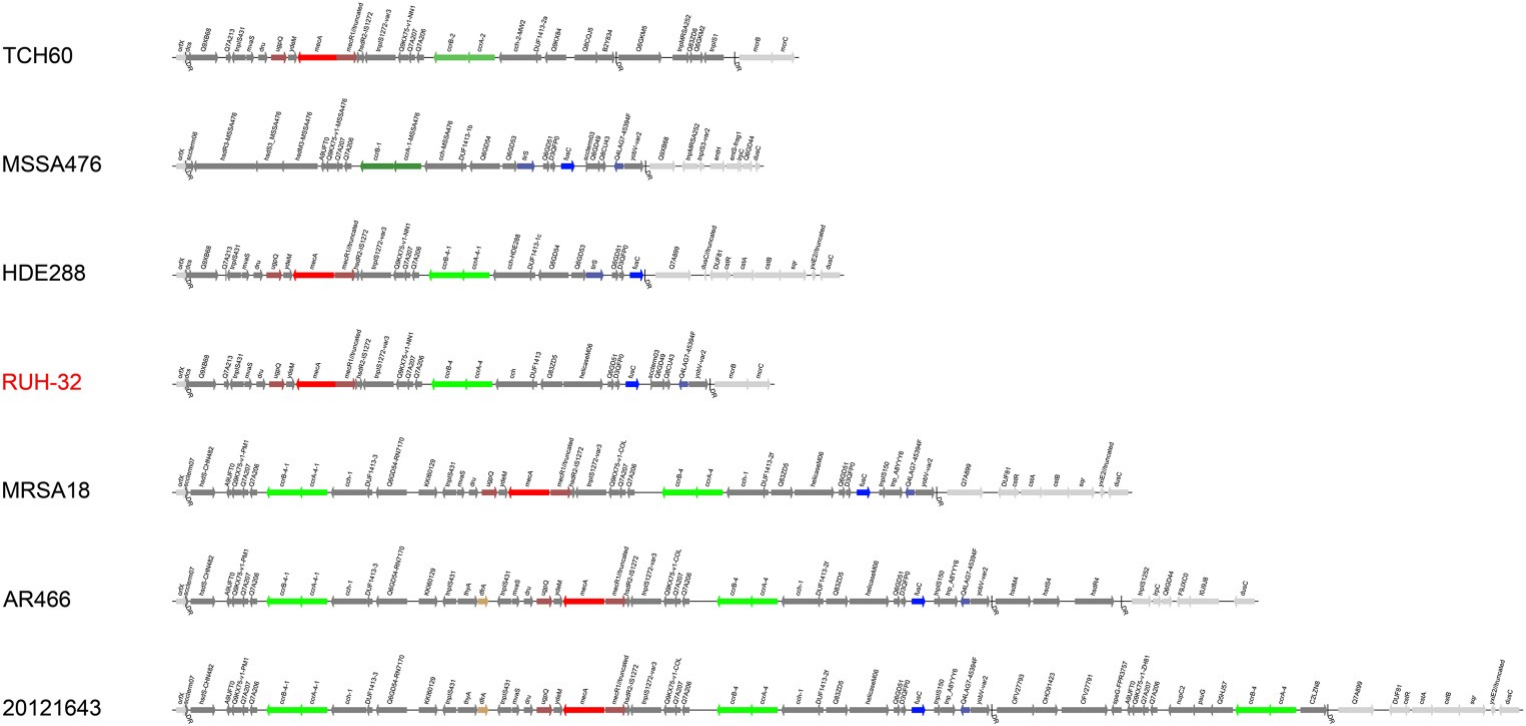
Schematic representation of the SCC*mec* VI _(RUH-32)_ element and, for comparison, of the SCC*mec* IVc element of another CC30 strain (TCH60, GenBank CP002110), of the SCC*fus* element from a CC1-MSSA (MSSA476, BX571857) and of other SCC VI elements that include *fusC* (HDE288, AF411935; MRSA18, ERR108048; AR466, CP029080.1 and 20121643, ERR1595888/SAMEA3924203). Genes outside SCC are drawn in light grey, genes within dark grey. *mecA* is red, typical *mec* complex genes dark red. *fusC* and accompanying genes are blue. The *ccr* recombinase genes are indicated in different shades of green, and *dfrA* in brown.

An overview on the gene content and the order of genes in the SCC*mec* VI _(RUH-32)_ element is provided in Table 2 and a graphic representation is shown in Fig 1. In short, the element consists of a class B *mec* complex in which a *mecA*_BA000018_ (a N315-like allele of *mecA* [2]) is combined with *ugpQ* and delta *mecR1*. The fusidic acid resistance gene *fusC* is present, *tirS* is absent. In sequences of RUH-32, *ccrA/B-4* are present, but *ccrA-4* does not yield signals in array experiments. The reasons are polymorphisms in the probe binding site of *ccrA-4* in RUH-32. Several other SCC*mec* elements that include *fusC* can be identified among previously published sequences. One is present in the CC5 strain HDE288 (AF411935; [13]) and can also be identified in CC8 (“UK-EMRSA-12/13”) sequences ASARH101 (SAMEA1565121), MPROS978 (SAMEA2041631) and MPROS1215 (SAMEA2663833). The SCC*mec* VI _(RUH-32)_ element differed from the SCCmec VI _(HDE288)_ element in an absence of *tirS*. Another difference is the presence of Q4LAG7 (“putative protein”; BX571857.1, position 55452 to 55880) in the RUH-32 sequence which was also detected by microarray. Differences to SCC*mec* VI element harbouring *fusC* of the CC8 strain MRSA18 (ERR10804/SAMEA1317993; [12]) include the presence of Q9XB68-*dcs* and SCCterm 3 (rather than SCCterm 7). Furthermore, MRSA18 has two copies of *ccrA*/*B-4* genes. Strain AR466 (CP029080.1), a CC45-MRSA, has a SCC*mec* VI element harbouring *fusC* that could not be differentiated from the one in MRSA18 by array (hence, it is not shown in Table 3). However, it includes an additional *hsdS/M/R* (type I restriction-modification) operon and *dfrA* (dihydrofolate reductase), which usually is plasmid borne. Finally, there is another SCC*mec* VI-derived element in the CC8 strain 20121643 (ERR1595888/SAMEA3924203) that harbours *fusC*. It differs from RUH-32 in several markers (see Fig 1) including an absence of Q9XB68-*dcs*, presence of *speG* (spermidine N-acetyltransferase) and *dfrA* as well as a presence of three copies of *ccrA/B-4* genes.

**Table 2:** Genes in SCC*mec* VI _(RUH-32)_.

Gene	Description/gene product	Comments	Orientation	Start position	End position
*orfX*	23S rRNA methyltransferase with the SCC integration site being located at the 3' end of *orfX*	Identical (no mismatches) to GU235984.1[9:488]	FORWARD	1	480
DR_SCC	direct repeat of SCC	Identical to BA000033.2 [34252:34270]	FORWARD	462	480
Q9XB68-*dcs*	putative protein	Identical to AFEF01000013.1 [388744:390039:r]	FORWARD	661	1,955
Q7A213	putative protein	Identical to BA000033.2 [36062:36400]	FORWARD	2,370	2,609
tnpIS431–06	transposase for IS431	Identical to BA000018.3 [36435:37109:r]	REVERSE	2,641	3,315
*mvaS-*SCC	truncated 3-hydroxy-3-methylglutaryl CoA synthase	Identical to BA000033.2 [37179:37531]	FORWARD	3,388	3,740
Q5HJW6	putative protein	Identical to BA000033.2 [37629:37859]	FORWARD	3,838	4,068
*dru*	SCC direct repeat units	*dru* repeats 0–2d-2d-2g-2n-3a-3b-4e-4i-5a-5b-7b-7b. No match identified in *dru* database.	FORWARD	3,978	4,415
*ugpQ*	glycerophosphoryl diester phosphodiesterase	Identical to BA000033.2 [38288:39031]	FORWARD	4,617	5,360
*ydeM*	putative dehydratase	Identical to BA000033.2 [39128:39556]	FORWARD	5,457	5,885
*mecA*	penicillin binding protein 2a	Identical to BA000033.2 [39602:41608:r]	REVERSE	5,949	7,937
Delta *mecR1*	truncated methicillin resistance operon repressor 1/ signal transducer protein	Identical to BA000033.2 [41708:42682]	FORWARD	8,037	9,011
*hsdR2*-IS1272	fragment of type I restriction-modification system endonuclease	Identical to BA000033.2 [42683:42916]	FORWARD	9,012	9,245
tnpIS1272	transposase for IS1272 from isolate TCH	Identical to BA000033.2 [42917:44440:r]	REVERSE	9,246	10,769
Q9KX75-v1-NN1	putative protein	Identical to BA000033.2 [44576:45082:r]	REVERSE	10,905	11,411
Q7A207	putative protein	Identical to BA000033.2 [45097:45408:r]	REVERSE	11,426	11,737
Q7A206	putative protein	Identical to HF569097 [32048:32398:r]	REVERSE	11,824	12,174
UTR_*ccrB*	highly conserved 3'-untranslated region of *ccrB*		REVERSE	12,175	12,649
*ccrB-4*	cassette chromosome recombinase B, type 4		REVERSE	12,677	14,305
*ccrA-4*	cassette chromosome recombinase A, type 4		REVERSE	14,302	15,663
*cch*	cassette chromosome helicase		REVERSE	15,850	17,619
DUF1413	putative protein associated with *cch*		REVERSE	17,619	17,909
Q83ZD5	putative protein		REVERSE	18,080	19,150
helicaseM06	DEAD/DEAH box helicase domain protein		FORWARD	19,244	21,184
Q6GD51	putative protein	Related to BX571857.1[51926:52234]; six mismatches	FORWARD	21,441	21,749
D3QFP0	putative lipase/protease	Identical to BX571857.1[52281:52519]	FORWARD	21,859	22,017
*fusC*	fusidic acid resistance protein C	Related to BX571857.1[52820:53458]; one mismatch	FORWARD	22,335	22,973
sccterm03	terminus of SCC towards *orfX*	For a discussion of the SCC terminal regions, and its variability, see [9]	FORWARD	23,571	23,658
Q6GD49	putative protein within SCC		FORWARD	23,659	24,288
Q8CU43	putative protein	Identical to BX571857.1 [54788:55030]	FORWARD	24303	24,545
Q4LAG7–45394F	putative protein	Identical to BX571857.1 [55452:55880:r]	REVERSE	24967	25,395
*yobV*	transcriptional regulator	Related to BX571857.1[55861–56889]; two mismatches	FORWARD	25,475	26,404
DR_SCC	direct repeat of SCC		FORWARD	26,501	26,519
UTR_*mcrB*	5'- untranslated region of *mcrB*	Identical to BX571856.1 [92902:93144]	FORWARD	26,520	26,762
*mcrB*	type IV 5-methylcytosine-specific restriction enzyme subunit B	Identical to BX571856.1 [93145:94848]	FORWARD	26,763	28,466
*mcrC*	type IV 5-methylcytosine-specific restriction enzyme subunit C	Identical to BX571856.1 [94841:95881	FORWARD	28,459	29,499

**Table 3:** Hybridisation profiles (selected markers only) for CC30-MRSA-[VI+*fus*], other isolates with the SCC*mec* VI _(RUH-32)_ element and predicted hybridisation patterns for reference sequences. Column “A/S” indicates whether the isolate was characterised by array (A) or if a genome sequence was analysed (S).

**Isolate**	**A/S**	**CC**	**SCC*mec* type/subtype**	***ugpQ***	***mecA***	**Delta *mecR1***	***mvaS*-SCC**	**Q4LAG7 (fus)**	***fusC* (Q6GD50)**	***tirS***	**ACME and *opp3***	***speG***	***ccrA-4***	***ccrB-4***	**Q9XB68*-dcs***	**SCC*mec* Term. 3**	**SCC*mec* Term. 7**	***mco* (plasmidic)**	***copA2* (plasmidic)**	***arsB* (plasmidic)**	***cadA* (TN554)**	***cadC* (TN554)**	***cadD***	***cadX* (plasmidic)**
**Riyadh (RUH-) 32**	**S**	**30**	**SCCmec VI**_**(RUH-32)**_	**+**	**+**	**+**	**+**	**+**	**+**	**-**	**-**	**-**	**-**	**+**	**+**	**+**	**-**	**-**	**-**	**-**	**-**	**-**	**+**	**+**
**Riyadh (RUH-) 32**	**A**	**30**	**SCCmec VI**_**(RUH-32)**_	**+**	**+**	**+**	**+**	**+**	**+**	**-**	**-**	**-**	**-**	**+**	**+**	**+**	**-**	**-**	**-**	**-**	**-**	**-**	**+**	**+**
**Dubai_M25**	**A**	**30**	**SCCmec VI**_**(RUH-32)**_	**+**	**+**	**+**	**+**	**+**	**+**	**-**	**-**	**-**	**-**	**+**	(+)	**+**	**-**	**-**	**-**	**-**	**-**	**-**	**+**	**+**
**Kuwait_2017_17412**	**A**	**30**	**SCCmec VI**_**(RUH-32)**_	**+**	**+**	**+**	**+**	**+**	**+**	**-**	**-**	**-**	**-**	**+**	**+**	**+**	**-**	**-**	**-**	**-**	**-**	**-**	**+**	**+**
**Kuwait_2017_17555**	**A**	**30**	**SCCmec VI**_**(RUH-32)**_	**+**	**+**	**+**	**+**	**+**	**+**	**-**	**-**	**-**	**-**	**+**	**+**	**+**	**-**	**-**	**-**	**-**	**-**	**-**	**+**	**+**
**Kuwait_2017_17749**	**A**	**30**	**SCCmec VI**_**(RUH-32)**_	**+**	**+**	**+**	**+**	**+**	**+**	**-**	**-**	**-**	**-**	**+**	**+**	**+**	**-**	**-**	**-**	**-**	**-**	**-**	**+**	**+**
**Kuwait_4445–1**	**A**	**30**	**SCCmec VI**_**(RUH-32)**_	**+**	**+**	**+**	**+**	**+**	**+**	**-**	**-**	**-**	**-**	**+**	**+**	**+**	**-**	**-**	**-**	**-**	**-**	**-**	**+**	**+**
**Riyadh_52**	**A**	**30**	**SCCmec VI**_**(RUH-32)**_	**+**	**+**	**+**	**+**	**+**	**+**	**-**	**-**	**-**	**-**	**+**	**+**	**+**	**-**	**-**	**-**	**-**	**-**	**-**	**+**	**+**
**Dresden-10436836**	**A**	**8**	**SCCmec VI**_**(RUH-32)**_	**+**	**+**	**+**	**+**	**+**	**+**	**-**	**-**	**-**	**-**	**+**	**+**	**+**	**-**	**-**	**-**	**+**	**-**	**-**	**+**	**+**
**Riyadh_3497247**	**A**	**834**	**SCCmec VI**_**(RUH-32)**_	**+**	**+**	**+**	**+**	**+**	**+**	**-**	**-**	**-**	**-**	**+**	**+**	**+**	**-**	**-**	**-**	**-**	**-**	**-**	**+**	**+**
**HDE288**	**S**	**5**	**SCCmec VI**_**(HDE288)**_	**+**	**+**	**+**	**+**	**-**	**+**	**+**	**-**	**-**	**+**	**+**	**+**	**-**	**-**	**-**	**-**	+*	**-**	**-**	**-**	**-**
**Kuwait_2017_4703**	**A**	**30**	**SCCmec VI**_**(HDE288)**_	**+**	**+**	**+**	**+**	**-**	**+**	**+**	**-**	**-**	**+**	**+**	**+**	**-**	**-**	**+**	**+**	**+**	**+**	**+**	**-**	**-**
**Kuwait_2017_5250**	**A**	**30**	**SCCmec VI**_**(HDE288)**_	**+**	**+**	**+**	**+**	**-**	**+**	**+**	**-**	**-**	**+**	**+**	**+**	**-**	**-**	**+**	**+**	**+**	**+**	**+**	**-**	**-**
**Kuwait_2017_18848**	**A**	**30**	**SCCmec VI**_**(HDE288)**_	**+**	**+**	**+**	**+**	**-**	**+**	(+)	**-**	**-**	**+**	**+**	**+**	**-**	**-**	**+**	**+**	**+**	**+**	**+**	**-**	**-**
**Kuwait_2017_4924**	**A**	**30**	**SCCmec VI**_**(HDE288)**_	**+**	**+**	**+**	**+**	**-**	**+**	**+**	**-**	**-**	**+**	**+**	**+**	**-**	**-**	**+**	**+**	**+**	**+**	**+**	**-**	**-**
**Kuwait_2017_5145**	**A**	**30**	**SCCmec VI**_**(HDE288)**_	**+**	**+**	**+**	**+**	**-**	**+**	**+**	**-**	**-**	**+**	**+**	**+**	**-**	**-**	**+**	**+**	**+**	**+**	**+**	**-**	**-**
**Kuwait_2017_17841**	**A**	**30**	**SCCmec VI**_**(HDE288)**_	**+**	**+**	**+**	**+**	**-**	**+**	**+**	**-**	**-**	**+**	**+**	**+**	**-**	**-**	**+**	**+**	**+**	**+**	**+**	**-**	**-**
**Kuwait_2017_18255**	**A**	**30**	**SCCmec VI**_**(HDE288)**_	**+**	**+**	**+**	**+**	**-**	**+**	**+**	**-**	**-**	**+**	**+**	**+**	**-**	**-**	**+**	**+**	**+**	**+**	**+**	**-**	**-**
**Kuwait_4527**	**A**	**30**	**SCCmec VI**_**(HDE288)**_	**+**	**+**	**+**	**+**	**-**	**+**	**+**	**-**	**-**	**+**	**+**	**+**	**-**	**-**	**+**	**+**	**+**	**+**	**+**	**-**	**-**
**Kuwait_5635**	**A**	**30**	**SCCmec VI**_**(HDE288)**_	**+**	**+**	**+**	**+**	**-**	**+**	**+**	**-**	**-**	**+**	**+**	**+**	**-**	**-**	**+**	**+**	**+**	**+**	**+**	**-**	**-**
**Kuwait_5750–1**	**A**	**30**	**SCCmec VI**_**(HDE288)**_	**+**	**+**	**+**	**+**	**-**	**+**	**+**	**-**	**-**	**+**	**+**	**+**	**-**	**-**	**+**	**+**	**+**	**+**	**+**	**-**	**-**
**Kuwait_5771**	**A**	**30**	**SCCmec VI**_**(HDE288)**_	**+**	**+**	**+**	**+**	**-**	**+**	**(+)**	**-**	**-**	**+**	**+**	**(+)**	**-**	**-**	**+**	**+**	**+**	**+**	**+**	**-**	**-**
**Riyadh_39**	**A**	**30**	**SCCmec VI**_**(HDE288)**_	**+**	**+**	**+**	**+**	**-**	**+**	**+**	**-**	**-**	**+**	**+**	**(+)**	**-**	**-**	**+**	**+**	**+**	**+**	**+**	**-**	**-**
**MRSA18**	**S**	**8**	**SCCmec VI**_**(MRSA18)**_	**+**	**+**	**+**	**+**	**+**	**+**	**-**	**-**	**-**	**-**	**+**	**-**	**-**	**+**	**-**	**-**	**-**	**-**	**-**	**+**	**+**
**Kuwait_2017_5056**	**A**	**30**	**SCC*mec* VI**_**(MRSA18)**_	**+**	**+**	**+**	**+**	**+**	**+**	**-**	**-**	**-**	**-**	**+**	**-**	**-**	**+**	**-**	**-**	**-**	**-**	**-**	**-**	**-**
Strain 20121643**	**S**	**8**	**SCC*mec* VI**_**(Strain 20121643)**_	**+**	**+**	**+**	**+**	**+**	**+**	**-**	**-**	**+**	**-**	**+**	**-**	**-**	**+**	**-**	**-**	**-**	**-**	**-**	**+**	**+**
**Isolate**	**A/S**	**CC**	**SCC*mec* type/subtype**	***blaZ***	***erm*(C)**	***msrA***	***linA/lnu*(A)**	***aadD***	***dfrA***	***dfrG***	***tet*(K)**	***tst1***	***seb+sek+seq***	***sec+sel***	***sed+sej+ser***	***egc***	***lukF/S*-PV**	***sea***	***sep* (= *sea***_**N315**_**)**	***sak***	***chp***	***scn***		
**Riyadh (RUH-) 32**	**S**	**30**	**SCCmec VI**_**(RUH-32)**_	**+**	**-**	**-**	**+**	**+**	**-**	**+**	**+**	**-**	**-**	**-**	**-**	**+**	**+**	**+**	**-**	**+**	**+**	**+**		
**Riyadh (RUH-) 32**	**A**	**30**	**SCCmec VI**_**(RUH-32)**_	**+**	**-**	**-**	**+**	**+**	**-**	**(+)**	**+**	**-**	**-**	**-**	**-**	**+**	**+**	**+**	**-**	**+**	**+**	**+**		
**Dubai_M25**	**A**	**30**	**SCCmec VI**_**(RUH-32)**_	**+**	**-**	**-**	**+**	**+**	**-**	**+**	**+**	**-**	**-**	**-**	**-**	**+**	**+**	**+**	**-**	**+**	**+**	**+**		
**Kuwait_2017_17412**	**A**	**30**	**SCCmec VI**_**(RUH-32)**_	**+**	**-**	**-**	**-**	**+**	**-**	**+**	**+**	**-**	**-**	**-**	**-**	**+**	**-**	**+**	**-**	**+**	**+**	**+**		
**Kuwait_2017_17555**	**A**	**30**	**SCCmec VI**_**(RUH-32)**_	**+**	**-**	**-**	**+**	**+**	**-**	**+**	**+**	**-**	**-**	**-**	**-**	**+**	**+**	**+**	**-**	**+**	**+**	**+**		
**Kuwait_2017_17749**	**A**	**30**	**SCCmec VI**_**(RUH-32)**_	**+**	**-**	**-**	**+**	**+**	**-**	**+**	**+**	**-**	**-**	**-**	**-**	**+**	**+**	**+**	**-**	**+**	**+**	**+**		
**Kuwait_4445–1**	**A**	**30**	**SCCmec VI**_**(RUH-32)**_	**+**	**-**	**-**	**+**	**+**	**-**	**+**	**+**	**-**	**-**	**-**	**-**	**+**	**+**	**+**	**-**	**+**	**+**	**+**		
**Riyadh_52**	**A**	**30**	**SCCmec VI**_**(RUH-32)**_	**+**	**-**	**-**	**+**	**+**	**-**	**+**	**+**	**-**	**-**	**-**	**-**	**+**	**+**	**+**	**-**	**+**	**+**	**+**		
**Dresden-10436836**	**A**	**8**	**SCCmec VI**_**(RUH-32)**_	**+**	**-**	**-**	**+**	**-**	**-**	**+**	**-**	**-**	**+**	**-**	**-**	**-**	**-**	**-**	**+**	**+**	**-**	**+**		
**Riyadh_3497247**	**A**	**834**	**SCCmec VI**_**(RUH-32)**_	**+**	**-**	**+**	**-**	**-**	**-**	**-**	**-**	**+**	**-**	**+**	**-**	**-**	**-**	**-**	**-**	**+**	**+**	**+**		
**HDE288**	**S**	**5**	**SCCmec VI**_**(HDE288)**_	**+**	**-**	**-**	**-**	**-**	**-**	**-**	**-**	**-**	**-**	**-**	**-**	**+**	**-**	**-**	**-**	**+**	**+**	**+**		
**Kuwait_2017_4703**	**A**	**30**	**SCCmec VI**_**(HDE288)**_	**+**	**-**	**-**	**-**	**-**	**-**	**-**	**-**	**+**	**-**	**-**	**-**	**+**	**-**	**+**	**-**	**+**	**+**	**+**		
**Kuwait_2017_5250**	**A**	**30**	**SCCmec VI**_**(HDE288)**_	**+**	**-**	**-**	**-**	**-**	**-**	**-**	**-**	**+**	**-**	**-**	**-**	**+**	**-**	**+**	**-**	**+**	**+**	**+**		
**Kuwait_2017_18848**	**A**	**30**	**SCCmec VI**_**(HDE288)**_	**+**	**+**	**-**	**-**	**-**	**-**	**-**	**-**	**+**	**-**	**-**	**-**	**+**	**-**	**-**	**-**	**+**	**+**	**+**		
**Kuwait_2017_4924**	**A**	**30**	**SCCmec VI**_**(HDE288)**_	**+**	**+**	**-**	**-**	**-**	**-**	**-**	**-**	**+**	**-**	**-**	**-**	**+**	**+**	**+**	**-**	**+**	**+**	**+**		
**Kuwait_2017_5145**	**A**	**30**	**SCCmec VI**_**(HDE288)**_	**+**	**-**	**-**	**-**	**-**	**-**	**-**	**-**	**+**	**-**	**-**	**-**	**+**	**+**	**+**	**-**	**+**	**+**	**+**		
**Kuwait_2017_17841**	**A**	**30**	**SCCmec VI**_**(HDE288)**_	**+**	**-**	**-**	**-**	**-**	**-**	**-**	**-**	**+**	**-**	**-**	**-**	**+**	**+**	**+**	**-**	**+**	**+**	**+**		
**Kuwait_2017_18255**	**A**	**30**	**SCCmec VI**_**(HDE288)**_	**+**	**-**	**-**	**-**	**-**	**-**	**-**	**-**	**+**	**-**	**-**	**-**	**+**	**+**	**+**	**-**	**+**	**+**	**+**		
**Kuwait_4527**	**A**	**30**	**SCCmec VI**_**(HDE288)**_	**+**	**-**	**-**	**-**	**-**	**-**	**-**	**-**	**+**	**-**	**-**	**-**	**+**	**+**	**+**	**-**	**+**	**+**	**+**		
**Kuwait_5635**	**A**	**30**	**SCCmec VI**_**(HDE288)**_	**+**	**-**	**-**	**-**	**-**	**-**	**-**	**-**	**+**	**-**	**-**	**-**	**+**	**+**	**+**	**-**	**+**	**+**	**+**		
**Kuwait_5750–1**	**A**	**30**	**SCCmec VI**_**(HDE288)**_	**+**	**+**	**-**	**-**	**-**	**-**	**-**	**-**	**+**	**-**	**-**	**-**	**+**	**+**	**+**	**-**	**+**	**+**	**+**		
**Kuwait_5771**	**A**	**30**	**SCCmec VI**_**(HDE288)**_	**+**	**+**	**-**	**-**	**-**	**-**	**-**	**-**	**+**	**-**	**-**	**-**	**+**	**+**	**+**	**-**	**+**	**+**	**+**		
**Riyadh_39**	**A**	**30**	**SCCmec VI**_**(HDE288)**_	**+**	**-**	**-**	**-**	**-**	**-**	**-**	**-**	**+**	**-**	**-**	**-**	**+**	**+**	**+**	**-**	**+**	**+**	**+**		
**MRSA18**	**S**	**8**	**SCCmec VI**_**(MRSA18)**_	**-**	**-**	**-**	**-**	**-**	**-**	**-**	**-**	**-**	**-**	**-**	**+**	**-**	**-**	**+**	**-**	**+**	**-**	**+**		
**Kuwait_2017_5056**	**A**	**30**	**SCC*mec* VI**_**(MRSA18)**_	**-**	**-**	**-**	**-**	**-**	**+**	**-**	**+**	**-**	**-**	**-**	**-**	**+**	**+**	**-**	**-**	**+**	**-**	**+**		
Strain 20121643**	**S**	**8**	**SCC*mec* VI**_**(Strain 20121643)**_	**+**	**-**	**-**	**-**	**-**	**+**	**-**	**-**	**-**	**-**	**-**	**+**	**-**	**-**	**+**	**-**	**+**	**-**	**+**		

* *arsB* in HDE288: absent in SAMN03255441, but present in SAMN03255487,

**harbours additionally genes *erm*(A) and *ant*9, which are not shown in the table.

### CC30-MRSA-[VI+*fus*] strains in the Arabian Gulf region

The novel SCC*mec* VI _(RUH-32)_ element described herein was not present in all CC30-MRSA-[VI+*fus*] isolates. Instead, they could be categorized into three distinct clusters (Table 3). Isolates of one cluster carried the SCC*mec* VI _(RUH-32)_ element. They harboured the PVL genes although there was one exception. They also were positive for *cadD*, *cadX*, *blaZ*, *linA/lnu*(A) (again, with one exception), *aadD*, *dfrG* and *tet*(K). A second cluster carried another SCC [*mec* VI+*fus*] element that yielded the same hybridisation signals as expected for a previously sequenced element from the Portuguese CC5 strain HDE288 [13]. One major difference to SCC*mec* VI _(RUH-32)_ was the presence of *tirS*. All isolates from this cluster were positive for the gene encoding the toxic shock syndrome toxin, *tst1*. A majority of them also were positive for *pvl* genes (nine out of twelve) and the enterotoxin gene *sea* (eleven out of twelve). Other markers included the copper resistance genes *mco*, *copA2*, arsenic and cadmium resistance genes *arsB*, *cadA*, *cadC* as well as *blaZ* and (in some isolates) *erm*(C). A third cluster, comprising of a single isolate harboured another SCC*mec* element consistent to one previously described from a CC8 isolate “MRSA18” [12]. The Kuwaiti CC30 isolate was PVL-positive. It lacked *tirS* and *tst1*. Heavy metal resistance genes were absent and *tetK* was the only antimicrobial resistance gene present in addition to *mecA*.

### Other MRSA strains with the same SCC*mec* element

One PVL-negative CC8 isolate was identified in a patient in Saxony who had links to the Middle East. Based on array hybridisation results, it also carried the SCC*mec* VI _(RUH-32)_ element (Table 3). This observation prompted an extensive database search yielding four more CC8-MRSA sequences with SCC*mec* VI _(RUH-32)_ in the NCBI Short Read Archive (SAMEA2385458, SAMEA2385540, SAMEA2664046, SAMEA2664096). We also identified the same element, SCC*mec* VI _(RUH-32)_, retrospectively in a ST834 isolate (Riyadh_3497247, see Table 3).

## Discussion

We describe a novel variant of a SCC element that harbours determinants for both, methicillin/beta-lactam and fusidic acid resistance. It was identified when performing DNA-microarray-based typing of clinical strains from Saudi Arabia because of a previously unseen hybridisation pattern and further characterised by sequencing. This element is related, but clearly distinguishable (see Results) from other such elements observed elsewhere as well as in the studied region. Furthermore, there is evidence for its horizontal transfer as we were able to detect it in two further, unrelated lineages of *S*. *aureus*. On a practical level, the observations suggest that emerging fusidic acid resistance might hamper its use as topical treatment for staphylococcal skin and soft tissue infections. A replacement of this substance by mupirocin is not feasible as the usability of mupirocin itself is endangered by the spread of resistance [34, 35]. Other substances, such as betaisodona, polyhexanide or octenidine, should be considered. On a more theoretical level, a co-localisation of genes encoding beta-lactam (*mecA*) and fusidic acid (*fusC*) resistance on one potentially mobile genetic element is an interesting example for coalescence of two “selfish replicators” [36] for mutual advantage. A community use of easily available topical fusidic acid could thus select for *mecA* methicillin resistance and a hospital use of systemic beta-lactam compounds could select for *fusC*, conferring benefit to MRSA with such combined elements in either biotope. Thus it can be expected that such strains emerge in hospital as well as in community settings and that it will be very complicated to contain or to eradicate them once they are established in a population. Indeed, there is a remarkable variety of different types and subtypes of SCC*mec* elements that additionally harbour *fusC*, indicating ongoing emergence and evolution. Such elements have been observed in as much as twenty-two different clonal complexes of *S*. *aureus*, CC1, CC5, CC6, CC7, CC8, CC15, CC22, CC30, CC45 [*agr* I], CC45 [*agr* IV], CC50, CC59, ST72, CC88, CC97, CC121, CC152, CC361, CC779, ST834, CC913 and CC1153 from geographic regions as diverse as Germany, France, UK, Ireland, Portugal, Malta, Saudi Arabia, UAE, Kuwait, Australia and New Zealand. Regarding SCC*mec* VI elements that include *fusC*, there are at least five distinct variants (as represented by RUH-32, HDE288, MRSA18, AR466 and Strain 20121643; see above), and to the best of our knowledge, they have been identified in CC5, CC7, CC8 (“UK-EMRSA-12/13”), CC22, CC30, CC45, CC97, CC152, ST834 and CC913 from Germany, United Kingdom, Portugal, Kuwait and Saudi Arabia [9, 12, 13, 23, 29, 30, 32, 37–42]. Observations of SCC*mec* elements that harbour *fusC* geographically cluster in Western Europe, the Middle Eastern/Arabian Gulf countries, Australia and New Zealand. Whether this is caused by a sampling bias or related to formulations and usage of fusidic acid, or both, we currently cannot tell. However, given the current patterns of travel and migration, appearance and emergence of fusidic acid resistant MRSA cannot be ruled out anywhere. This phenotypic property cannot be seen any more as a surrogate marker for the presumptive identification of PVL-positive CC80-MRSA-IV (“European”/Mediterranean clone) but it should prompt further investigation. As mentioned above, co-evolution and co-selection of resistance traits that favour resistant pathogens in hospitals as well as in the community outside pose a public health hazard. This should prompt a review of the use of topical antibiotics such as fusidic acid (or mupirocin) including restrictions to uncontrolled and unlimited over-the-counter sale of such compounds.

## Supporting information

S1 File Full sequence of SCC*mec* VI _(RUH-32)_ as well as of assembled sequences of MRSA18 (ERR10804/SAMEA1317993) and strain 20121643 (ERR1595888/SAMEA3924203). (PDF)
