# Supplementary material for: Characterisation of a novel SCCmec VI element harbouring fusC in an emerging Staphylococcus aureus strain from the Arabian Gulf region
Source: PLoS One. 2019 Nov 5;14(11):e0223985. doi: 10.1371/journal.pone.0223985 (PMC6830749; doi:10.1371/journal.pone.0223985)
Supplement: S1 File — (PDF) [file pone.0223985.s001.pdf]

<u>Supplemental file 1:</u> Full sequence of the SCC*mec* VI $_{(RUH\text{-}32)}$ element. *mecA* is shaded black, with white letters. *fusC* is shaded grey. The gap region that was sequenced conventionally is indicated in lower case letters. Assembled sequences of MRSA18 (ERR10804/SAMEA1317993), page 9, and strain 20121643 (ERR1595888/SAMEA3924203), page 22, are additionally included.

> SCCmec VI (RUH-32)

ATGAAAATCACCATTTTAGCTGTAGGGAAACTAAAAGAGAAATATTGGAAGCAAGCCATAGCAGAATATGAAAAACGTTTAGGCCCATACACCAAGATAGACATCATAGAAGTTCCAGACGAAAAAGCA
CCAGAAAATATGAGCGACAAAGAAATTGAGCAAGTAAAAGAAAAAGAAGGCCAACGAATACTAGCCAAAATCAAACCACAATCAACAGTCATTACATTAGAAATACAAGGAAAGATGCTATCTTCCGA
AGGATTGGCCCAAGAATTGAACCAACGCATGACCCAAGGGCAAAGCGACTTTGTATTCGTCATTGGCGGATCAAACGGCCTGCACAAGGACGTCTTACAACGCAGTAACTACGCACTATCATTCAGCAAA
ATGACATTCCCACATCAAATGATGCGGGTTGTGTTAATTGAACAAGTGTACAGAGCATTTAAGATTATGCGTGGAGAAGCTTATCATAAGTAATGAGGTTCATGATTTTTGACATAGTTAGCCTCCGCAGT
CTTTCATTTCAAGTAAATAATAGCGAAATATTCTTTATACTGAATACTTATAGTGAAGCAAAGTTCTAGCTTTGAGAAAATTCTTTCTGCAACTAAATATAGTAAATTACGGTAAAATATAAATAAGTACA
TATTGAAGAAAATGAGACATAATATATTTTATAATAGGAGGGAATTTCAAATGATAGACAACTTTATGCAGGTCCTTAAATTAATTAAAGAGAAACGTACCAATAATGTAGTTAAAAAATCTGATTGGGA
TAAAGGTGATCTATATAAAACTTTAGTCCATGATAAGTTACCCAAGCAGTTAAAAGTGCATATAAAAGAAGATAAATATTCAGTTGTAGGGAAGGTTGCTACTGGGAACTATAGTAAAGTTCCTTGGATT
TCAATATATGATGAGAATATAACAAAAGAAACAAAGGATGGATATTATTTGGTATATCTTTTTCATCCGGAAGGAGAAGGCATATACTTATCTTTGAATCAAGGATGGTCAAAGATAAGTATATGTTTCCG
CGGGATAAAAATGCTGCAAAACAAAGAGCATTAACTTTATCTTCCGAACTCAATAAATATATTACATCAAATGAATTTAATACTGGAAGATTTTATTACGCAGAAAATAAAGATTCATCTTATGATTTAAA
AAATGATTATCCATCAGGATATTCTCATGGATCAATAAGATTCAAATATTATGATTTGAATGAAGGATTCACAGAAGAAGATATGCTAGAGGATTTAAAGAAATTTTTAGAACTATTTAATGAATTAGCTT
CAAAAGTTACAAAAACATCCTATGATAGCTTGGTCAATAGCATAGACGAAATACAGGAAGACAGCGAAATTGAAGAAATTAGAACAGCACAAAAAGATAAGACACTCAAGGAAGTGGAAGCACCTAAA
GGAATAATTCCAAAATATAAAAAAGGTGTATCAAAGACTACTAAAAATGATTCAGAAATTGAAAAATCAAATAAAGAGAATAAATTAACCGGTAAAGTTGGAGAAAAATTAGCGCTAAATTACTTTAAT
GAGCTAATTGATAATAAAATAGACGAAGATAAGAAAGAACAGTTTAGGAATATTTTAAATGATAATCCAGGCTCTCAACACGGTCATGGCTATGATTTAGTAGCTTTTGATCCAACAAATACAGATAAAG
CTGTAGAAAAATTTATTGAAATTAAAACATCTACATCTTCTAGTATTGAGGAACCATTTTTTATGTCGCTAAATGAAATGTTTGCTATGAAAGAATATAAGCAGAAATATTTAATATTAAGAATATTTAAT
GTTTCCGGTAAAGAACCACAATTTTATTTTATAGATCCATATGCAAATTATTCTGAATTTAAAGATGTAGATGATCTCATTGACAAAGTATTTAATGTAGAAGCTATTCAGTATAAAGTTTTTGGCGAAAAA
TGATTACTTGAACAAGAGCTAAAATAAAATTGTGATCTAATAAAAATAGAAACTGTAATTTAAATAAAACTTTCTAAATAAGCTAACTGATAAAAAATCAGTTTGTCCACAGTCTGAAACAAGATTCCTA
TATTCTTTAGGAATCTTGTTTTTTCTATTTTTATGGTGATAAAGAGCAGATAAGATAATGTGTAATAATCACAAAAAAGTTAAATATTTTAAGGCTTGTTTAATTATTAATGATTTTATATATAAAGAGCAG
TATAATAAAGTTGTTAATATATTATGAATAATATTCAAGTAATTTTATTGTTTTTTAATTTGTCGATATTTAAGTTGAGTTAAATTTAAAGGGTGTAATTTGTTTTACAATGATGAAGATAATTAGTCTATCA
AAATAAAGGGGTTGGGACTGTTATGAGTGATAATTTGTCATTATTCATTGACTATATCAATGATAATATAATCTATGGTAGTGAAATCAAACGGGAGAAATTAGAGAATTTATTTAATCAATTTGCTATAA
AAAATGTTGAAAAGAACATTGTCTATGATGAACTGAAATCTTTAGATATTACAATCATTGAGTCACAGGATTCATATAAAAATAAATTGAAGAGATTATTTTCGGTTCTGTTGCAAAGTAAAAAAATATAG

CTAACCACTAATTTATCATGTCAGTGTTCGCTTAACTTGCTAGCATGATGCTAATTTCGTGGCATGGCGAAAATCCGTAGATCTGAAGAGACCTGCGGTTCTTTTTATATAGAGCGTAAATACATTCAATAC

CTTTTAAAGTAttctttgctgtattgatactttgataccttgtctttcttactttaatatgacggtgatcttgctcaatgaggttattcagatatttcgatgtacaatgacagtcaggtttaagtttaaaagctttaattactttagccattgctaccttcgttgaaggtgcctgatctgtaattaccttttgaggtttaccaaattgtttaatgagacgtttg

ataaacgcatatgctgaatgattatctcgttgcttacgcaaccaaatatctaatgtatgtccctctgcatcaatggcacgatataaatagctccattttcctttattttgatgtacgtctcatcaatACGCCATTTGTAATAAGCTTTTTTATGCTTTTTCTTCCAAATTTGATACAAAATTGGG

GCATATTCTTGAACCCAACGGTAGACCGTTGAATGATGAACGTTTACACCACGTTCCCTTAATATTTCAGATATATCACGATAACTCAATGTATATCTTAGATAGTAGCCAACGGCTACAGTGATAACATC

CTTGTTAAATTGTTTATATCTGAAATAGTTCATACAGAAGACTCCTTTTTGTTAAAATTATACTATAAATTCAACTTTGCAACAGAACCGTATTATGGAATAGAGATGTTGGTAACATTTATACAGGATCAT

TATACTTAAGTTTAATTTCGTTATTACAGAACCACACATTCCAACCAGAAGAGAAAGTATGTCTATTTAGTTATGGTTCAGGAGCAGTAGGAGAAATCTTTAGTGGTTCAATCGTTAAAGGATATGACAAA

GCATTAGATAAAGAGAAACACTTAAATATGCTAGAATCTAGAGAGCAATTATCAGTCGAAGAATACGAAACATTCTTTAACAGATTTGATAATCAAGAATTTGATTTCGAACGTGAATTGACACAAGATC

CATATTCAAAAGTATACTTATACAGTATAGAAGACCATATCAGAACATATAAGATAGAGAAATAAACTAGTGGCCGATTGTGCTTGATGAGCTTGGGACATAAATCCTAACTCGAAATAAATAAGCATAT

CACTAAACTGATTTTTTAAAGTTTACAGTGATATGCTTATTTTTTTATCTTACGATTTTGTACGTGCATGCTTGCCTAGGGGTATGGCTCGAGCCATTAGTCTCTCGCACATACTATTCCCTCAGGCGTCAGC

ACTTACAAAATCGGTTGTAATTTTCATTTTTATACGCATTCTTACTGAGATTATACTAATAAGAGGAATAGTAAAAGCAATTCTAAGTAAAATTGCAGATAAGAGGTTTGTTAAAAGCAGTTCTAAGTAAA

ATTGCAGATAAGGGGTAAGTTAAAAGCAGTTCTCAGTAAAATTACAGATAAGAGGTACGTTAAAAGCAGTTCTAAGTAAAATTGCAGATAAGAGGTTTGTTAAAAGCAGTTCTAAGTAAAATTGCAGAT

AAGAGGTACGTTAAAAGCAATTCCATGCAAAATTGCTGATAAGGGGTAAGTTAAAAGCAGTTCTCAGTAAAATTGCAGATAAGAGGTACGTTAAAAGCAGTTCTAGGCAAAATTGCAGATAAGAGGTGC

GTTAAAAGCAGTTCTCAGTAAAATTGCTGATAAGGGGTAAGTTAAAAGCAATCCTAAGTAAAATTGCAGATAAGGGGTACAGAAAAACTAGACTTGATTACAAAATGGAGCTTGGGACATAAATGATTT

TTTAAAAATGAGATGAGACGTAGATTAACTCCATAATCAATACGAATCTATCGACTTCTTTATTTATGATATTCATCTCTTTTTAATGGAAATAAAAGTGCGATTAATGTGATAATACAGTTACGTTAATTA

AAAAAATAAAAATGCAAGGAGAGGTAATATGCTAACTGTATATGGACATAGAGGATTACCTAGTAAAGCTCCGGAAAATACAATTGCATCATTTAAAGCTGCTTCAGAAGTAGAAGGTATAAACTGGTT

GGAGTTAGATGTTGCAATTACAAAAGATGAACAACTGATTATCATTCATGATGATTATTTAGAACGGACTACAAATATGTCCGGGGAAATAACTGAATTGAATTATGATGAAATTAAAGATGCTTCTGCA

GGATCTTGGTTTGGTGAAAAATTCAAAGATGAACATTTGCCAACTTTCGATGATGTAGTAAAAATAGCAAATGAATATAATATGAATTTAAATGTAGAATTAAAAGGTATTACTGGACCGAATGGACTAG

CACTTTCTAAAAGTATGGTTAAGCAAGTGGAAGAACAATTAACAAACTTAAATCAGAATCAAGAAGTGCTCATTTCAAGCTTTAATGTTGTGCTTGTTAAACTTGCAGAAGAAATCATGCCACAATATAA

CAGAGCAGTTATATTCCATACAACTTCGTTTCGTGAAGACTGGAGAACACTTTTAGATTACTGTAATGCTAAAATAGTAAACACTGAAGATGCCAAACTTACTAAAGCAAAAGTAAAAATGGTAAAAGAA

GCGGGTTATGAATTGAACGTATGGACTGTAAACAAACCAGCACGTGCAAACCAACTTGCTAATTGGGGAGTTGATGGTATCTTTACAGACAATGCAGATAAAATGGTGCATTTGTCTCAATAGAAAGTTA

GAGGTGAGTCTTACGTTTCAGTGACGGTAGACTTACCTTTAACATGTTACATACTAAAAAATTAATTTGAATAAGAAAGAGAGACATATATGAAATACGATGATTTTATAGTAGGAGAAACATTCAAAAC

AAAAAGCCTTCATATTACAGAAGAAGAAATTATCCAATTTGCAACAACTTTTGATCCTCAATATATGCATATAGATAAAGAAAAAGCAGAACAAAGTAGATTTAAAGGTATCATTGCATCTGGCATGCAT

ACACTTTCAATATCATTTAAATTATGGGTAGAAGAAGGTAAATACGGAGAAGAAGTTGTAGCAGGAACACAAATGAATAACGTTAAATTTATTAAACCTGTATACCCAGGTAATACATTGTACGTTATCG

CTGAAATTACAAATAAGAAATCCATAAAAAAAGAAAATGGACTCGTTACAGTGTCACTTTCAACATACAATGAAAATGAAGAAATTGTATTTAAGGGAGAAGTAACAGCACTTATTAATAATTCATAATA

AAACAGTGAAGCAACCATCGTTACGGATTGCTTCACTGTTTTGTTATTCATCTATATCGTATTTTTTATTACCGTTCTCATATAGCTCATCATACACTTTACCTGAGATTTTGGCATTGTAGCTAGCCATTCCT

TTATCTTGTACATCTTTAACATTAATAGCCATCATCATGTTTGGATTATCTTTATCATATGATATAAACCACCCAATTTGTCTGCCAGTTTCTCCTTGTTTCATTTTGAGTTCTGCAGTACCGGATTTGCCAAT

TAAGTTTGCATAAGATCTATAAATATCTTCTTTATGTGTTTTATTTACGACTTGTTGCATACCATCAGTTAATAGATTGATATTTTCTTTGGAAATAATATTTTTCTTCCAAACTTTGTTTTTCGTGTCTTTTAA

TAAGTGAGGTGCGTTAATATTGCCATTATTTTCTAATGCGCTATAGATTGAAAGGATCTGTACTGGGTTAATCAGTATTTCACCTTGTCCGTAACCTGAATCAGCTAATAATATTTCATTATCTAAATTTTTG

TTTGAAATTTGAGCATTATAAAATGGATAATCACTTGGTATATCTTCACCAACACCTAGTTTTTCATGCCTTTTTCAAATTTCTTACTGCCTAATTCGAGTGCTACTCTAGCAAAGAAAATGTTATCTGATG
ATTCTATTGCTTGTTTTAAGTCGATATTACCATTTACCACTTCATATCTTGTAACGTTGTAACCACCCCAAGATTTATCTTTTTGCCAACCTTTACCATCGATTTTATAACTTGTTTTATCGTCTAATGTTTTGT
TATTTAACCCAATCATTGCTGTTAATATTTTTTGAGTTGAACCTGGTGAAGTTGTAATCTGGAACTTGTTGAGCAGAGGTTCTTTTTTATCTTCGGTTAATTTATTATATTCTTCGTTACTCATGCCATACATA
AATGGATAGACGTCATATGAAGGTGTGCTTACAAGTGCTAATAATTCACCTGTTTGAGGGTGGATAGCAGTACCTGAGCCATAATCATTTTTCATGTTGTTATAAATACTCTTTTGAACTTTAGCATCAATA
GTTAGTTGAATATCTTTGCCATCTTTTTTCTTTTTCTCTATTAATGTATGTGCGATTGTATTGCTATTATCGTCAACGATTGTGACACGATAGCCATCTTCATGTTGGAGCTTTTTATCGTAAAGTTTTTCGAG
TCCCTTTTTACCAATAACTGCATCATCTTTATAGCCTTTATATTCTTTTTGTTTTAATTCTTCAGAGTTAATGGGACCAACATAACCTAATAGATGTGAAGTCGCTTTTCCTAGAGGATAGTTACGACTTTCT
GTTTCATTAGTTGTAAGATGAAATTTTTTTGCGAAATCACTTAAATATTCATCCATTTTTTTAACGGTTTTAAGTGGAACGAAGGTATCATCTTGTACCCAATTTTGATCCATTTGTTGTTTGATATAGTCTTC
AGAAATACTTAGTTCTTTAGCGATTGCTTTATAATCTTTTTTAGATACATTCTTTGGAACGATGCCTATCTCATATGCTGTTCCTGTATTGGCCAATTCCACATTGTTTCGGTCTAAAATTTTACCACGTTCTG
ATTTTAAATTTTCAATATGTATGCTTTGGTCTTTCTGCATTCCTGGAATAATGACGCTATGATCCCAATCTAACTTCCACATACCATCTTCTTTAACAAAATTAAATTGAACGTTGCGATCAATGTTACCGTA
GTTTGTTTTAATTTTATATTGAGCATCTACTCGTTTTTTATTTTTAGATACTTTTTTTATTTTACGATCCTGAATGTTTATATCTTTAACGCCTAAACTATTATATATATTTTTATCGGACGTTCAGTCATTTCTACT
TCACCATTATCGCTTTTAGAAATATAACTGCTATCTTTATAAACTTGTTTGAAATTTTTATCTTCAATTGCATCAATAGTATTATTAATTTCTTTATCTTTTGAAGCATAAAAATATATACCAAACCCGACAA
CTACAACTATTAAAATAAGTGGAACAATTTTTATCTTTTTCATCAATATACTCCTTATATAAGACTACATTTGTAGTATATTACAAATGTAGTATTTATGTCAAAATAATGTTATAATTTTTGTGATATGGAG
GTGTAGAAGGTGTTATCATCTTTTTTAATGTTAAGTATAATCAGTTCATTGCTCACGATATGTGTAATTTTTTTAGTGAGAATGCTCTATATAAAATATACTCAAAATATTATGTCACATAAGATTTGGTTAT
TAGTGCTCGTCTCCACGTTAATTCCATTAATACCATTTTACAAAATATCGAATTTTACATTTTCAAAAGATATGATGAATCGAAATGTATCTGACACGACTTCTTCGGTTAGTCATATGTTAGATGGTCAAC
AATCATCTGTTACGAAAGACTTAGCAATTAATGTTAATCAGTTTGAGACCTCAAATATAACGTATATGATTCTTTTGATATGGGTATTTGGTAGTTTGTTGTGCTTATTTTATATGATTAAGGCATTCCGACA
AATTGATGTTATTAAAAGTTCGTCATTGGAATCGTCATATCTTAATGAACGACTTAAAGTATGTCAAAGTAAGATGCAGTTCTACAAAAAGCATATAACAATTAGTTATAGTTCAAACATTGATAATCCGA
TGGTATTTGGTTTAGTGAAATCCCAAATTGTACTACCAACTGTCGTAGTCGAAACCATGAATGACAAAGAAATTGAATATATTATTCTACATGAACTATCACATGTGAAAAGTCATGACTTAATATTCAAC
CAGCTTTATGTTGTTTTTAAAATGATATTCTGGTTTAATCCTGCACTATATATAAGTAAAACAATGATGGACAATGACTGTGAAAAAGTATGTGATAGAAACGTTTTAAAAATTTTGAATCGCCATGAACA
TATACGTTATGGTGAATCGATATTAAAATGCTCTATTTTAAAATCTCAGCACATAAATAATGTGGCAGCACAATATTTACTAGGTTTTAATTCAAATATTAAAGAACGTGTTAAGTATATTGCACTTTATGA
TTCAATGCCTAAACCTAATCGAAACAAGCGTATTGTTGCGTATATTGTATGTAGTATATCGAGCTTCACATGAAACAGCTAAAGAAGCTTTGGGCGATAAAGAGTTAAGAGCCATTGCACATGAGTTAACT
AAAACAGTTAAGGATAACATGAGTGTTGATTGGTCTAAACGAGACAGTGCTAAAGCTAAAATGAGAGTTCAAGTTAGACGCCTATTAAAGAAATATGGCTATCCACCAGATCTTCAAAAAATGGCTGTGG
AACAAGTTGTAGAGCAAGCAGAATTAATGGCAAGTCAGCAATAAAAAAATAAATCATAATGAGTCCGGGACATAAAGTTCTTGGATAAGTGAAAAAAGACAATTTCTATTGAAATAATATAGAAATTGT
CTTTTTTATAAATTTTTTGATTATTTTCAGCTCGTTGAGCTACTACTTTTCTTATATTAAGTGCCATTAATACAAAACCAAGTTCTCTTTTGACTTTATTGAGTCCTCGGACAGACATCCGAGTGAAACCCAA
AATAGCCTTCATAAATCCAAAAACAGGTTCCACATCAATTTTTCTTTGACTGTAGATATTTTTTGTTTCTGGTTCTGAAAGCTTTTTGTTAATTTGGGATTTAAAATATTCCCAGTTATAATTCTTCATTATTT
TTTTGTTTGTTTTTGAATTGAAGTTCATACATTGATTTTTCAGAGGACATTCTGAACAATCATCACATTCATATAATTTGAAGTCTCGCTTATAACCATACTTATCATGACGATAGGCATATCTTTTAAAACC
TAGCCGTTTATTATTCGGACAAATGAATTCGTCATTAATTTCGTCATAGTTCCAATTTTGAGTATTAAAGATGTCACTTTTATATTTTTTAGTTTTATCTTTTATAAACATTCCATATGTTATGAGTGGCGTTC
GATTAAAGTCATCTATAATTGCCTTATAATTTGATTCACTACCATAACCTGCATCAGCTACAATATATTCAGGTAAATGACCGTAGGTCTCTTGAATTGAATTTAAAAATGGAATCATCGTTCTAGTATCCG
TTGGATTTTGATACACATTATAAGATAAAACAAATTGGGAATTTGTTGCTATTTGTAAATTATACCCTGGCTTAAGTTGTCCATTTTTCATGTGATCTTCTTTCATTCTCATAAATGTCGCATCATAATCTGT

CTTAGAATAACTATTTCTATCCTTTAAAATAGATTTTTGAAATTCGTATCGATACTTTCGCTCAAAATAATCATTGATTTGCTTTTTGTATTTTTTGATTTTAGTTCTTTTGAGACGTATTTGTTTTCTTGTTTT

AGTACATTTTTCATTGTTGATATGTTGGTTTAAATCTTCGATTTCTTTATCTAAGTGACTACCAATCAAATCTATTTCTTCTTTTGTTAATTCATTATCATGATCTTCTTTAATTTCCGGTATGATTTTATTGGT

TACCAATTCATGGTAGAGGGCTTTAGAATCCTCATTCATCTTTGATTCATGGTTTTGAATACTCTTTTTCCATACAAATGTATATCGATTGGCATTTGCTTCAATTTTTGTACCATCAATAAAAATAGCTTTA

TCATCTATAAGATTTTGTTTTACACACTGACTGTAAAATTGAATAAATAAAGATTCTAATAAAGCATCTACTTTTGGATTTACTCTAAATTGATTAATTGTTTTATAAGAAGGTTTTTGATTTTGTGATAGCC

ACATCATTCGGATGCTATCATTAAGCATTTTTTCTATTTTACGACCTGAGAATACAGATTGTGTGTAGGCATATAGAATCACTTTTAACATCATTTTAGGATGGTACGAAGTTGCACCACGGTGATGTCTGA

ATTCGTCGAATTCATTGTCAGGAATTGTTTCAACAATATCATTTACAGTAAAACGATGTTGATTTGTTTTGTTTCCATATTGACCTCCATGTATTTGCTATGATTTCAAAATCCATTTTTGACGTGCCTTAGG

GTTGAGTGGATGCATAATTTCATTTGTTACTGGATTGATGAGCTTTTTTACTTTCTTTTTATGAGGTTTTAACATTTCCATCACTTGTTCGACACGGTCGATAACAACTGGTCGCTTCGCATAGGCACCATAA

GCAAGAATCACTGTGTCACTTTCACTAATCGCTTTCATCAAATGAATATCAGTGTGCTCATCGTATGGATTTTTGATATGTTTGAGGTTTTCGGGTGTTCTAATATTAGAGAATAGATTTACAAGATATACA

GCACCGTATCGTTCTGAATTGGCTAATTGGTTGAGGATAAGAACAGTTGTGAGATCGAGTGATAATACACCGTCTAAATGAGGATACATCGTTATCACTGTGCATGCAGCTTTCTTTTCATCCCATGTTTTC

TTGAGTAAATAGCGGTGCTGTTCATCATCGCTAAATATGGCTTCTGTGTGTATCGTACTTTTGATTGTATTCATCATCGTCACTTCCTTTAGTATTCTTCTGGTAAAAGCATCACATAATAAAAAGCGTCCAC

GTCATCTTCACGAATGACGTAGACTTTCTTAGGTAATGCATTTTGATTTTTTTCATAGTTTGTATAGTGATATTCCAATTTGTATGTGGGTTGTTCTTGTTCATGTGTGATTGAGAGTATATTCTCATCTTCTT

GTAATTTAAAAATGTGTAGGTAATCTGTATGAGGCTGGTTATCTTTTTTTTCTACCATGTGCCAAAGTAAGATTTGAAGGTCTAGTGGAATACTTTCATTAATTCCTCTTGTGATGTATCGATTGATTTTCAT

GCTATTTCCCTCCCTTCTGCTTTTCTTTCATGATGTCGATGATTTCGTTGATAACTGTGACGGATAATTGAGCAACTATGATCCAATTATTCATGGTCCTGACCTCCTTGTTTTAGTAAATGACGTTCATCAAT

AATGATATTTTGAGTATCTGTAAGGTACAGAAAGTCCATGTCAAAATGGTCTAAGTATCCGACACTGATGAGTTGGTTATTGGCATACATTAGAAATGGATAGATACTTAGCTCATGTAGTTCATCATTAT

AGTAGGTATAAGTCTCGAGTGTGAGATGTACCAGTGGAGAATCATTAATAAAACGTTCCGGTAGAATATTTTCTGCTGCTTCCTCCAGCGCTTCACATTCCCAAGCTTCGTTATTAGATAGTTGGAATAGG

TGGGTTATATATTGTTTGAGTTCTTGAGTGGTTGTTTTCATATCATTGCCTCCTAGATAGTGTGATAGTGATGTAGTTGATATACATCATTGGGATAATATATATTTGATTTATTATTTATTACGAATCCCGG

TGGGAATAAGAGAAAATTCCATTTGAAAAACCGCTACAAACGTTGATATGACAAGGAAATCCCGAAATTCCGCCTATTTTGACGAACAATCAACTCATTATTTATAAGTATTGATGATATGTGTAGGTCTC

TCTGCTTCCTTATATATATTATTTATTTATAAAGAATAACGGGATTTTGGGATTGTGCTTGCACACTCCTTCTGCTTCTTCGAAACTGCAAATCCCATTTCTTTCCCGGTAAAAAATCATTGTGGGATGTTCT

TTAGCAATTTCAATATAAGCATCGTGTAGTCATGAAAAAAATGACGACAATGACTGTTTCATTAGATAAGTGTTATTGAAATTGATAAAGAGAATTCTAAAAATGGTTAGATAAAATAAGTGAAAGAATA

GCAGTGTAGTTATTGTTTATTCAATAGTTATATATAAAGTTTGTGGCAAAAATAAAGACGAAGTGCTAGGGAGCACTTCGTCGAGTGGATGGTTATTAAATAGTTGTTTGATAATATCATTATTCAACTTG

AGCGTAACATAAAATTGATTTTTATGCTGTTCATGTTTTCGGATATCAATACGATCGATAACCGTCAGATACAATGATTTCAACTGTGATTTTTCTAATTTGTCTATATCTTTGAATATTGCTTGTAATACAT

TAGCAATCATATCAGCATCATAATGTGAAGCTTCCGCTTGTTTATCTTGTTCTAGTTGATGGATTTGATTATTTATTTGATTCAATTCATCTTGGTAGCTAAGTATTGTTGGTTTCAATACGCTATCTAAGTC

AGGTGAGTCTTCAATAGTTTTCGTTAGTGTATGCATTTTGGCTTTTATTTCTTCACATTGTGACTGTTTATAGGCAATATCATGATTCAAAGAAGAGACGTCTATTTGACTTCTTTCATTTACTTTTTCAACCA

ATTGTTTCAATACCTTTTTACTTTTGATAATTTCCAATATTTGGTCCATAACATATTTTTCTAATACATTTGCTCTAACGCTATTGGCTGAACAAACTTTTGAACCTTTGTTTCTAAAATTACTACATGAGTAA

TATCTGATTCTTTTCTTAGTGCCATCTTTTAACGTATTGGTCGTATTACTTGCCGCCATTGCTGCACCACATTTCGGACATTTTACAATGCCAGTCAGTAGGTTTGTCCCTTTACCGTGAACTTGAGGTTTCTT

GCGACTCTCTTGGCGTTTAAACTGTACTTTATCCCATAAAGCTTTATCAATAATAGGCGCATGCTTACCATCTGCAATAATCGGTTCTTCATTGAGTCCTTTTCGTCTTTTGTCGCTCCAGTGTCTATACTTCG

CAAACTGTATTTTTCCAATGTAAAAAGGGTTTGAGATGATGTAAGTAATGGACGAAATACTAAAAGGTTTCCCTTTCTTAGTCACATAACCTTTATGATTCAATGCGTTCGCAATCTTACGATAACCATGAC

CTTTAGCATATGACTCAAAAATATACTTAACAATATTCGCTTCATGTTGATTGATCATGAGCTCTTTTTTACTGTCAGGTACTTTATCATAGCCTAGAGGCAAATTACCTTGGTAATAACCTTCAATAGCTCG

TTGTCTTTGGCCATTGTAGACATTCTCTACAATCGTATTTCTTTCAAATTCTGCGAAGCTGGCTAAAATTTGGAGCATCAATTTACCTGTTGAGCTGGCAATTTCTATTTTTTCAGTCAGACTAAAAAATTCG
ACATTGATTTTATATAATTCTTCGACAATATTTAATAAGTCTGAGGTATTTCTAGCTAAACGATTTGTTTTGTAGACCATAATACAATCTAATTTACCTTCGTTGGCATCTTTTAACATACGTTGTAATTCTG
GACGTTGCATTGTTTTACCCGATATACCTCGATCAGTGTATTCATTGACGACTTCATAGCCTTGAAATTGACAATACTCTGTAAGTTGATTCAATTGACCTTGAATACTGTAACCATCGGTTTGCATTTCAGT
CGATACACGTGCATATAATCCAATACGTTTCTTTTTGAGTTGTTTCATATGACTTCATTCCTTTCAAGAGTTATTAAATGCGATTGTTCAACGATATTGAGTGGACTGTTTTTAAAGTAGATCCCTTGTAATT
GTTTAGTTTGAGTTATTGTAATGTCATCAATCAAAGGTGCTATGGTTTCTAATGTCAATTTATCTTTGATGACATGACGGATTGCTTCGGTAATTTGATTAGGTGAATAATTAGAGTAACGTTCTTCACTTTC
AGAGCTAGTAGATAACCGTTTAAATGTTTCTATATCAATTTGATTTTGTGCTAGTTTTTCGATGAGTTGTTCTTGTGTTAGATGTGATTGTCTGTGTTTCATTTGTTGTCGTTTTAGCGCTTTTAGTATTGTCT
CATTTAATTTGTCATGAAATGATTGATTATCACAATATGCTTTACAAGTGTTGAGGACCTCTGTTTCAATCATTTGCGCATTGATACCTTTAAATGGACACGTATGATAAGCTTCATTCATATTTTTAGGACA
GACATAGTAACGTAGAGAATAATGTTCTTTTTTTATCGTTAAGTTTGTTAATGTTGATTGACAGTAAGGACATTTGATTTGTCGCTTTAGCTTATTTCTAGAATTGGATCGATTGTGTTGTTTGTGAATACGA
CGCTCTTGTGCTTCTTCAAACGTATCAACATCAATAATAGATGGAACGATATCATTAAACGTTCCTTATTTATTAATGACACGACCACAATAGTTAGGGTTCAAGAGAATATTTCGAACTTGATAGGGCTT
ACGAGGAATAAGCTTAGGATGGTTATCTAAATGTTGGGAAATCTTTTTGTAGCCTATACCCTGTAAGTACCAGCGATAGACCGCTTTGACTGTATACGCTTCTTCTTCATGTACAATAAAATGACTTTGTCT
ATAACGATAGCCAAACGGAGCATGAGTTGTGATTAGTTTACCTTGTTTGGCTTTTTCTCTTATCCCATTTCGTGTCTGTTCACTGATATTATTAGACTCCATTTCAGCTAGACTCATAAGTATGTTTAGGCGA
AAGCGATCAAATTCTTTAGATAAGTCAAAATAGCCATCATTCACGCTGATGATTGTGACGTGATGCTTTTTACAAATTTCAAAGAATTGTATGGCATTTTTCAAATTACGATGGAGTCGATTCAAGCGATA
GCAACACAATACTTTACATTTTCCAGACGTAATCATTTCTACCATTTTTTGATAACCTGACCGTTTCGTATGTCGACCGCTTTTCTTATCATCATAAAACGTTACATTAGGCCATCCATATTGCTTAGCAGTA
TTTAAAATCAGAGATTGCTGAGTAGCTAAGCTTTGTTGTTTGAGTGTACTTTGACGTACATAACCAATGGCTTCTACCATGTTATACACCTCCAAAAAGATAATATATATTTGTGAGTAAATTAGAATGAA
AGGTCCAACGTGCTTTTAACACGTTGGACCGCAATGATTAATTATCGTTCTGTAACTCTTCAATAACTAAATCAGCTAGTAATTCAATCAATTCATCCATTTTATCTACTCCTGTACGATTGCATCTTTAAGT
TATTAAAAATCAATTAAATCATCATTCTGTTTTTTCCATGATTCAAGCATCTTTTTGTTATCCAAGTTAATTTTAGCTTTAATAGGTTCAGCATCTTTAGTTAGACCAAAAATAGATGCATATTCTGAATCTA
GTTTTAAATGGTAAAAGACAAGTGACTGTTTCTTACCATAGTCATCTTTAACTGTTCGTTTTGTCGTAATTCGATCACGGTCAGATTCAATAAAACCTTTATCTCTTAGTGCATTCACGACATTGTTGACATC
CTGAAAGTGCTGTTCTTTCAGCATATCTTTAAATACAGTCGCAATGATTTTGACTTCGATATAATCATCTTTTAAGGCAATTAGTCCATAATTCTCAATCATATTCTTTAACGCTGTGTCATCAGAAAACTTA
CCACGATTTTGTGCTACAAATTGCGTAACGACTTCAATCGCTTTATCTGCCAGTGATCGTTCAGAGACTGTATGAGTATGATAATCAATAAAGTAATCTCTTATTTTAGCGATATCAATATTTGTAGCTAAA
ACACGACCTAATATTTGCGCAGATGTTGTAATGACTGCATAACGCTTAAACATACGAATACCTGTATTGTTTGCTTCACTCTTCAATTTAGCTTTAAACCAATGATGTTCATCTTTAAACCATTGAATAACT
TCCTCTTCACGATTTATAAGGTATTGAGCTACTAACGGTAAAACATGACCATAGTTTAGTGCCACAGCTTTTTTAATATTGTCAGCATTGGTCGCATTTGTAGTAAATTGCTCATTAATTTCAATGGTTCTTA
CACGTAACCCATCGTTTTGAGCTGAATCAGTAAAAATACTGTATTCTGACGTTGAAATGACAGAAGTTCCCCAATTCTTAGGTGTTTTAACTTCTCCATGAACGTTTGAACGTTGACGACCTTGACCTTCAG
CGATGGAGTACAATAACCCTGTTGTATCTTTAAAAGTTGCTGAGGAGAGCTCGTCAAATACCATAGGTATGCCATAATTGTTACTCAAGTAACCTTCAAGAGCGTTACGTGTGGCATTCCAACTTCTAAAA
AGCGTTTGATTACCTTTGGTTGGATTTCCAGCGACGGATACCGCTAAAGCTGCCGCTGTTGATTTACCGGTTGATGACTGGCCTGTAAAACTAAAGATGATACCAGCAAATTCAACCTCATGTTTATGCTTC
AGAAATGCTGTCACTAAGGCAGAAATTCCAAATACGACCGCTAACTCTAGAAGAAGATGACCTTTGACTTCGTTCATATACATATTTAACCAATCTTTAAATGTCCCTTTAGGCTCTAATAGATAAGCACT
ATCGACAATAGGATCTAAAGATGCTGACCGATCATATTGTTTAGATGTGTAAACGGTATCTATCATTACAATGTAACCGTAGGGTGTTTCTATGATGCCCGAGCCATCATATAAATCAGAAGTGGGCAGTT
CATCACGCATTAATTGTAGTGCATAGCTCAAATCTCTTATATAACTTTCATTGATACTGTGACCATATTTAATCAAAGAAGGTAACTTTTGAGTTGTTAAAATATCAGATTCAAATGTATGTTCTTTATCTTT
ACCGTTAGAAATAATTATCTTTTCTGTATTTTCTGAGGCATGCCAAAATTTAGCTTCAACAACGATACAACTAGATATAGGCACCACTTTTTCCTTATCTCCATCTTTTTTAGGTGGAAAAGTTTTACGCCAC

GCCTTTGAATCTAATTGGTATGGACCTACTTCAAAAATGATGTAATGACTCATTAGCGAACACCTCCTTTCGAAGAGTTGCTATCATTGAGTGGATTAGGTCCTATTTTTTTATACAATAAACTACCATTTTT

TTTACCAATAATAATAAAAGGAATGCTAGGTGTATGTTTTACGAAATAAGCGAACCAACGTCCTACCTCGCGTTGAACATCTCTAGAACAGTTAAGGCCTGATTTTTTAGTTAAATCACCAATTGTAAATT

CAGTGCCTTTCTGTAAATTATTTGCAATAGCTAAATCTGCATTTTTTAAATTTATAAATTCTTGACTTTTATAAGTCATAAAAATCACTCCTTACTTAATTGCTAGTAAAGTGACTAATTACTTAATCAACTT

TACACTTGAATTATTGCAAGGATCAGAATTTTAAGTCATAGAGAGTTTTAATAATACTTTTTATCGATTTAATACATTAATGTATTAACACAGTAAAGTATAAAAGGAGAGATAAAATGAATAATTGGAAA

AATGAGTTGTTCGAAAGATTAGGAGATATTATTGAACTTGAGATTGCAATACATCTAATGATTAGGAAACATGAACGAAATTCGATAGATAAATATAAGAACATTAAAGAGTTTTACCATAATCTAATAC

AGAAGGACATAGTTATTTATGATTATGAGACATTTCATCGATTCTATAAAACGAGTAATATTCACAATTACTTACGGGTAAAACTAAAGCCAAAAAAAGCTTGGGAAGAAGAAAAAGTCATTCATCCAGA

ATCATTTAAAAGTATCTTAGCAATACGTTTAGGTGAAACAAAATTGATGGCAGGGACTAAATACATTGACTCCAATATTAAAAGCTTAAGCCAAACTTATAAACAACTAAATTCTAATATTGAACAGATTT

TAGATAAAATAAGTAAGAATACATTTGACATAAAATTGGAACCAAAGTTGATTGCTTATAAAAATTTATATGAGTTATACAATTATTTTATGGACGAAATAGAAAACTATAGCAACGACTTTGAGCAATT

ACGAGCGCTTCTATATTTAGAATTAGATTACTCATTTGTACTTTATAACGAATTATTAAGGATGATACATTTAATCAAAAAGTGTAAACTAGCTGCTAATAGCAAGATAATAGAAAAAGCAATAGGTGAA

ATTAGTATGAAGTTAGCAAATATAGAATTTCCATGGCTAAGATTAGTAGTTCTAAGAGCTTTTATTGATAATATAAAAAAATATCATGATATAAATTCATTGATTAACGATATTAGTCATATTATCAATTAT

ACGTTACTTGAAATCGAACAGTGGATTGAACGAGCAGAAATTGAAGAAGGAATTAGAGATTTAATGAATGTGAAATATGGTAGGGTTCCTTATCTAGATGCAATTATAGAAAACAGATATAAAAGAAAT

AGTATTAAAGAATATTTTACCTTACTCAAACATGAATTTGGAATTAGATGGTTAAGAGGTCGAGACTTTAATGATCAAGATAGGAACACACTTCATAGAGCTATTAAAGATATATCTCTTAATCTCATTGG

ATACAATAACTAACTTAAATACGTTATCTACGAGTAAAAACTTATAAAAAGAACATGTGTTCTTAATTAGGGTATAATTAGTTTAAATTGATTTTGGGGTGATGTAATGAATGAGAGTAATCAAATTATAT

TCGATCATATAACAAAGGCTATTGAAAATATGAAAAATAAAGGCTGGGAAACAAGGTATGGGCAAGAAGCTTTAATGTATGATGTATTTGATGCTTATGATTCAAGAGAAAATTTAATAGTAGAAGCGC

AAGTAGGTATAGGAAAATCATTTGGATATTTAATTCCTGGTATATTGATTTCAAAAAGTACTAAAAAACCTTTAATTGTAACGTCTTCCTCAATTCAATTAACTGAACAACTAGTCAATGATATACAGCAA

GTTGAAGAAATTTTACATATATCTGTTGATTGTATAGTAGGTAAAGGTGTAACTAACTATCCTTGTTTTAAAGAAATTCATCGTAAAAATTTAAGTAAATTAAATTTAATGGAGGCTTTAGATATAGCAAA

TAAAGGTTTAACAAAACAAACTGTACGTGAGACTAATTTAAAATGGAATCAAATATCTACCAATAATTGTATTATGAGTAAATGTCACTATAAAAATGAATGCGCTTATTTTAAAATGAGAAATAAGTTA

AAAGAAGGAAATAGATATATTAGACTCAATGAATATAAGCCGAAAGTCATAATCGTTAATCAAGATATGTTAATGATGAACTTTAAAAAGTTAACTTTTGGAAAAGAGAGTTTAATCTATGATGATCCTT

GTATGCTAATCATAGATGAAGTTCACAACTTAGAAGAGAAACAACGTGCTAACATGACTAAAACTATTAACTCTAAAACTGTGATAAATAAAATAAAAGAAGGCGCAAACCGAGTTGGTAGTAGATCTC

GGTATTTAAAAAATATAAAAATGATTGAAGATTGGTTTAATCTTCAAAAGGATAGTGCCAAAAGTGAGATATACAAAAATGGTAATTGTTTATCTACTGGACGAGTTGATATAAAACCTGTTAGTCATCAT

CAAATGGCCAAATTAATTAGTATGACAAAGGAAATAGTCGAAGAATTCGATGTTAATAATATTGCTGTATTTAAACAATCTTCGTTAGTTAGTAATGAACAATTAGAAATAGCTAATAATCTGTTAACATT

ATTTAAAAATCTTCAAAATAACAGTGACAAATATATATTTTGGACTGAAATAACTAAGCAAGACCAAATTGATATTTCATTTTGTCCCAATAATATTGCTGAAACACTTAGAAAAACAGTTTTTAGTACAA

GTTACCCAGTTGTATGTCTTTCTGCAACAATTACTAATAAAACTAATAATGAAAATAGCTATGAGTACATTAAAGAAATAATTGGTTTTAAAGGCTATGAGGAAGATATTAAGTATAATGATTTTCCGTAC

AAGGAAAGCAGGCTCTACATACCTCCAAACTTACCAAAATTTGATAAACGTGATGTTAAATATTATGAAGAAATAGGTAAACATATTTTTGAATTGGCAAGCCAAAATAAGGGAGGCACTTTGATATTGT

TTACTGCTAAAGATGACATTGACGGTGTATATAATGATTTATCAAAAAGGAAATTTAATAAAACTATCTATGTAGATGATGGTAGTAAAAGTCAAAATGAAATTATTGAATCATTTAAAAAAACTAAAGG

TGTAATATTGGGTACGGGAGTATTTTGGGAAGGAATAGACTTAAAAAATGAATTATTAACATTATTAGTTATTGTCAGATTACCTTTTCCTACGATTGATCCAATTACAAAATATAAGATTACTAAATTAA

ATGATAGTAATGAAGCAGTTATAGTTCCTGAAATGATAATTAAACTGAAACAAGGTGTAGGAAGATTAGTAAGAACAAAGCAGGATAAAGGTTTATTAGTGTTATTAGATTCAAGAATGAATAAACCCAT

TTATAAGCATAAAGAAGCAGTATTAGATGCATTACCAATTAAAAATATGATTCATAAAGATGAAGTCAAAGATTTCCTTAATAACATTTAAAGTAAGTAGTAATAGTTATCCAATATTTTCTAGTTGATTC

TCTTATAATATGTCCAATTAAGTGTAGACGATTCAAGATGATATTGAGGGAAGGATGAGAATGTGTCTTTCAAGTAGCTCATTTGATGATAACCTTAAACATAATATTTACTTTCATCCTGCTGAAAAAAG

AGGTTACAGTTTCAAGCCGATAGAATACATTGGGCTATATAAAGACAAAGCAATAAAAGCTATCGGAAAAGTAGACAAAGTAATAGTGACGGAAATAAATGAGAGCTCATTATCCTTGAAGACAGTTTA

TCCTGTAGGTACTGAGTTATCGATTGATGAGTGTGAAATAGTTAAAAATAAAATAATGGTGCTTGGGAAAGAAAAGTGGACTAATCTATTGAACGAACCCCATTATTATTACTTAATTGAAGATTTTATAG

AGACAGACTACAAGAAAACATCTAAAGGTGGATCGATGGGAGTTAAGTATTTTAATGTGAATGAAATTTTGAATAGGGGTTGCTTAACCACCGAACAAATCGCTAAAGAATTATGTAATAAAGATTGGG

AATGATAAATAGTGAATGAAGCTATAATGCAATAATGTATGTATAACATAATCAAGTAATGAATTTAATGATAAGAGAGTATGGAGTAAAGAATTGAATTAGTTTGCATGGTCTATGTATCTTAAAGGTG

TTGAAGGAGATACACCGAAGTATGCAGTACCAATTCTTGAAACAGACTTTAGTGATTTACCATCTACACATACGACAGTCGGATCACTTGATCGGTTTCGAGATAAGACAATGAGCATTAACAGAAGCGG

GAGTAACTGTTGAATTCCATTTATATCCAGTTAGATACTAGAGTTTGAAGTGATAAATCTTGATTCGGATGCTGAAATAAGTAAAAGAGCAGTTACTGAATATAATATAAGTGTAAATATGCCACTAGATA

TTTTAAGGAGTATTTATTCACTTTTATAAAATCATCTGCTAAGATAGTATGGAAGATTTTTATAGGAGCTGATTATAATGCGACATATAAATATGTCATACATCATACGCTATAGTACTCTAACTCAAAGAT

AAGAAGTACTTAGTTATGAATAGCATTCATTTCACAAAGTACTTCAACAAAAATGGAGGAATATGAAATGAATAAAATAGAAGTGTATAAGTTTGTTAAAGTAAAGCAGTTAGTATATCAATTGATTAAG

TTATATCGTACAAACGATATGAATTCCCATAAAACACAAAAAGATTTTTTACTAAATGAAATTAATGATATCTTTAAAGAAAAAGATATTGATATCTCGGACTTTATTACATCGATTGACGATGTAAAATT

AACTAAGAAAAAAGCAGAACATCTTTTAAATGAATTAAAAGTGTACATCCAAGATTTTGAAATACCTTCATCAAGTCAACTGGAGAAAATTTTTCGTAAAGTAAAAAAATTAAAAAGACCAGATATAAAT

TTAATTGATACAAAAGAAATTTCATATTTAGGATGGAATGATAATTCTTCTAACCGAAAATATATCGTTTATAAAAATTTAGATGATAAATTCGAAGGTATATATGGCGAAATTTCACCAAATAAAGTAAA

AGGATTCTGTAAAATTTGTAATCAGGAATCTGATACATCACTCTTTCTCAATAAAACTAAACATAATAAGAGTAGTGGAACATATACTAAAAAAGGAGATTACATTTGTTATGACAGTTTTAAATGTAATC

AGAACCTAGATGATATAAATAATCTTTACGAATTTATTGTTAAAATAAAATAGATCCAAACAGCCCTGATCTTTAGAACTAATGATCAGGGTTGTTATATTTTGTACCTCACTCTTAATCGATACGCTGAAA

GTCCCTATAAATAGAACGTACGTTCCGTTTGTGGTATAATGACAAAAATATTAAATTAGAGGATTAAAAATGGAATTATAATTTTTTAATGTACCAAACTGAAAAAGGCTAAATAAAGATAGATATAAGA

TTAGAAAAAACTGTATGGATTAGGCAATATTTATATATAATGAAATGAATTAACAGAAAATCGAATTATCCGGAAAAACCGTACTGTTCGATTGGAAGGGAATAGAGAAGTTGAAAAGAATTAACTTCA

ATTATCATCTTATTTCTGAAAAAAGATATGAAAAACGGACGCAAGACACTTTAAATATTAGTAACAACGTAAAGTGAATAAAATCTATATTAAGGCATCGTGATGAATTTAATATAATTGATAAAAATGA

TTAATTATCCATTTTATTTATAATATATTATTTTTAGCAATTGTATGTATATTTTTCTTGATTTACTCTAATAGTACAGAAACTATAAACAACAATCTTCTAAAATACGCTGAAAGTTATCATAAGTGATGGT

AAAAAAATGAGTAAGTAGATGAGGAGTGAAAATCAGATTAATTAATAATAATGTATCAAATTTAAATAAAGGGGGTCTTTAAGTATGAATTTAAGAGGTCATGAAAATAGACTTAAATTTCTTGAGAAAT

ATGATGTGACACCTATATCACATTTAAAATTATTAGAAGGTCAAAAGAAAGACGGTGAAGGCGGTAGACTGACAGATAGCTATTATTGTTTTTCATACAGTTTAAAAGATAATTCTAAAAAAGTTTTAGGT

ACGTTTAATTGTGGTTATCATATTGCTGAAGATTTACTAAAATTATCAAATCAAGACAAATTACCTTTATTTAACCCGTTTAAAGTAATTAATAAAGGTAATCAATTGCAAGGCGTAACGAATAAAGGTAA

TTTAGATATTAATAGGCAAAGAAAACAGTATAATGAAGTGGCTTTACAGCTTTCAAATGCTATTAATTTAATCATAATTTGTTATGAGGGTAATATTAAAGAACCACTTTCAACGATAAAATACGAAACCG

ATAAATATTATTATAGTGAACCATATGATAGTAAAATTAAAGCAGTAAATACTATTATAGGTAATTTGTTTGATAAGAAATTAGTTGAGAAGATATCTGAAATTAATGTAAATCAGGAAATTAGAAAATTT

GATTTTTCATTACTCACTAATAAAATTAAAAAATATGAGATACAAAATAATTTTGAATAAGTGGAGGTAAGTCAATATTAAATATAGAAGCTAGTATTAAGGATATTCAAGATATTATAAATTATTTTATT

AAATGTGACGTTAATAATAAGTATTATGAAAGTAAGCAAAGAAGCAGTCTCGAAAAGTTAGCTTTAAAATTGGATAAAGTAAGTAATCAGTTAAACAATGAAAACATTGAAGGTATGAAAAAGATAATT

GAAAAAGTAGCAGAAGCAAATAGAGTTATCGCTAAATCTTCTTTAAGAAAAAAATTGAGTGTTTTAGTAAGATGATTGAAGTTGATAATAAATCTACTAATCAAAAGAAATTTTAAATATATATTGAGTTA

AAACTTAAAAAACTTATATCTAAATGAAGAAACGGGAAACCATTCTGAATCCTCACCCTTTCCCGTTCCAAAATCATTGATAATGAAAAACATCGCTTATACGGGGTATTGCATATTTTGCCTTAATTTCA

AATACTATACATATAATAGAATCACAACTAAAAGCACGTCATAACTGTACTGTTCATTCTGTACAGTAGGTATTAAACTATTAAATAAGTATAAGATTAAACGAAAGCCAAGATGCATAATGTCATGGCTT

TTTATATTGAAAAAACATGCGATAATTTGTTCCCAAATGTCCTTATAGTTGTACCAATATCTGTAATAGAACACTAGAAACTTGGTTTAACCTTTCCTTTTCGTAAATAAATGTAAAGATATACCATAAGGA
TCTCTTACATAACCATAACCTTCAGTATAGAACTCTGGGCTGAATGCTTTTAATACTTCACTGCCTTTTTCTATTAACTGGTCATACACACGCTTAGTTTCTTCTACATGGTCAAAAGTAATACAAAGCGATA
TATTATTACCTTGGGTTATAGGTAAACCTTCGGTATCATCCGCAATCATAATTTTTATATCTCCAAATTGAAGTACACATTGATCAATTTTATTTAAATCTTTTTTATCGATATTAAGTTGCTTATCTGTCGGT
CTATCCTTGATACGTTGGATATACAGTGTTTTAGCGCCAAACAGCTCTTCATACAACCTTTTCAAACCATCTGCATTTTCTATAATTAAAAAATAGGGACTTACTTGAAAATTCATAGTTTTTCCTCCTTAAA
TTTGTTATAAATAAGTATATACTGTATTAGTGTCACCTATTGATACTAAAAGGGGAATAATAATGAAAAAATCTGTTAGATTATATCATATGATTGAATACTGTAATGAAAAAAGGAATTTCAAGTTAAAT
GATTTAATGTCAGAATTTAATATTTCTCGTAGTACTGCTTTAAGGGATATAAAGGAAATTGAAGCATTAGGAGTGCCTTTATATAGTAGTACAGGGAAAAATGGCGGTTATATGACCATAGGTAATCGAA
ACCAAACGAAAATAGCAATCACAGACGAAGAATTGAAAGCTTTAGTATTTACACTTTCAAGTATCTCCAATGTTAGTAAGTTACCTTTTCAAACAGAATATCAAGAAATATTAAAAAAATTATATAATAA
TTCGAATAAAAAAGAATTGATAAACCAGTATAATGAGATATTTCAATATTTTAATGAAGATACATATCAGTTCAAAAGTTATAAGATATTTAATGAAATCATTAGATTAACAATTGGTAGTAAGTCATTTA
AAATCTGTTATTCACAAAACTATTTGAAAGAACAATATAAAGGTATTGGTATCTTATATAAAAATCAACAATGGTACTTCGTTGTTGTTAATATAGCATCAAAATTAGTAAAGTTGTTAAACATTTCTAAA
ATAAAAGAAGTATATGAAATGGGAGAAACTAAAGAATGTAATGAAATAACTATGCAGAATTTTCAACAGTTTATGGTTGAAAACGAAAATACGATTGATATACAAATTAGAAGTAATATTATTGGATTA
AATATCTTGAAAGGATACTTGTGGAGTGACTATATGATTGAAAATATTAACGAAGAGACATATTTGTTTAAATCAAAAGTGAACATGCAAGATATAAATTTTATAGCTAAATTAATAGTCACATGTGGTGC
CAATGTCAAAGTAGAATTTCCTATTAGTTTGAAAAAAGCTATTAAAACTGAACTAACTAAAATAATAAACTTATACTAATGGTAATTTAATTATGATTGCAATAACAAATAAAAGATAATCTTAAACATAT
TAAATTGATCCGTAGTATAAAAACCGCATCATTAACCGATACGCAGAAGCGTATCATAAGTAGCGGAGGAGTTTTTTACCTTGTGACTTATCATAAAGTACGATGTTTATGTAAGTGATTATCATTATTTA
AGCAGGTTTTTCAAATTAAATAATAACAAGAATAAAATGCACTTAGCGACATTGAAATTTATTAATCTAGTAAACTAATAGATTTATAGAAAATTTTATTTGCAAGGGGATAATTTTGAAAAGTAGTATTT
TCTATCTTTCCATAATACATTGTAATTACAACGGAGGGGATATTGTGATGAAGTGTATAGATAAAACGTGGGTTAGCTATTATAAAGAATTAGCTGATAAGTTAACAGATTATCAAAATAAACGTTATGAA
TTAATTGAAATAGTGAAGGAAGTATATAAAAAAACGGGAATAAAATTCCCTACTTTAGCAAGTGATAATGTATTGATGGACATAGATCCTTTTACAATATTTGCATTATTTAATAAAAATTCCATGAGAGA
AACTAATAAGGTAAAAATATTAACAGAATTAGCTTCGGAATTGAATATTAAGTCCAAAATTCCGTCAGTTTTTGACAGTATTCCAACAGTCAATAATCTGAATGCTACATATTATAATTTTAAAGATTATG
GAAAAGATAATGATATAGAGACCTTGTGGACCTTATTTACTGCTGCATTAAATTTTGCGAATTATCCTTCAGAAGATAATAAATTAAAATTTATACATAGTTTTGATAAGAATATTACTAAAACAGGAAAT
GGGAATAGTAAGATTACGAGTGGTTTGTACTGGATTGCACCTGAAACTTTTGCTAACTTTGATTCACGTTCAATTTGGTACATTTTTGATACATATAAACTCCCTAAAGAATTGATTAATGAACAACTTAAA
CCAAAAGAAAAACTAACAGGTCGACATTATTTAGAATTGATAAATATGTTAAAGCAAGTTTTGAAGTGCCAGGAAGTAGAGATTAATGATTTTATTGATTTAAGTTATGAGTCTTGGCATTATTCAGAAAT
GATAAATGAAGCTAATAGAAACAAATTTGATTCTAATATCAAAGAAATTGTTGTCAGTGAAAATGACAATATAATACAAAATAGAAGTAGAACAACTAAAGTTATACCATATGGAAAACAAGAATTCTT
AGATGAAGTTTTTATTGATGAAAGTGATTATGATAGGTTAGTACAACTTTTAAGAAGAAAGCGAAATGTCATTCTACAGGGACCGCCTGGTGTAGGAAAAACTTTTCTTGCAAAACGTCTTGCTTATTCAT
TGATTGGCAGTAAGTGTAAAGAACGAATTAAATTAATTCAATTTCATCAAAGTTATTCATATGAAGATTTTATTATGGGATATAGACCTACTAGTTCAGGTTTTGAACTGACTACAGGGACGTTCTATGATT
TTTGTAAGAAAGCTGATAAAGACCGCGATAATGAATATTTTTTAATTATTGATGAAATTAATAGGGGTAATCTGAGTAAAATTTTTGGTGAATTATTTATGTTGATTGAAAGTGATAAAAGAGATGAAGAA
CTAATATTACTTTATAGCGGTGAGAGATTTTCTGTACCTAGAAATATTTATATTGTGGGAATGATGAATACAGCAGATCGTAGTTTAGCAATTTTAGATTATGCATTACGCAGACGCTTTGCGTTTTATAAT
ATGCAACCGGCGTTTCATACAACTAAATTCAAAAAACTTCTTAAAGCAATTAATAATACTGAATTAGAGTCAGTAATTAATTGTATTGAAGGATTAAATAAAGAGATTGTTCAAGACGATATGTTAGGTA
ATGGCTTTGTTATTGGTCATAGTTATTTTAGCAATATTGAATGTATCGACAAAGATGAGCTTTCTAATATTATAGAATTTGAGATTATTCCTATGTTAGAAGAATATTGGTTTGATGAACCTTCAAAAATAA
ATGTATGGTCTGAAAAGTTAAGGAATTCAATAAATAATGATTAATATTAAAAATATTTATTATATGTTATCTTATGCATTTACAGTACTTAACAAAAAAGGCTATCAGAAGTTAGCAACAGAACAATTTGA

AAATATATTTGATTTATATTCAGCTATTCTTATAAAAGGTATATCAAGTCAGTTAAATAGTGGACTTCATCATGAATATATAGAACAGACAGATTCTCTAAAAGTTATTAGGGGAAAAGTTGATGTTAAGA
ATTCTATACAAGGCTTAGGAGTTTTAAGTCAACGTATTAATTGTATTTATGATGAATTTTCATTAAATACATACATGAACAAGATTTTAAAAACTACAATGAAATGTTTAATAAAAACAGATATTTCTAGA
AAGAATAAGATAAAGCTCCGAAAGTTATTAGTTCATTTTAATAATGTTGATACTTTAGATTATAGAAATATTCAATGGTATCATTCGTTTGATCGAAATAATCAGACGTATAAAATGTTAATATCTATATGC
TATTTAATTTTTCAAGGAGTAATACAAACTGAAAGCAAGGGACAAAATGATCTTATGGTATTTGTAGACGAACAACAAATATCTCGGCTATATGAAAAATTTATTCTAGAGTACTATAAAAAAGAATTTCC
AGAGCTTGTTGTAACATCATCAAATATTCAATGGTCATTAGATAATGATGATAACGTGAATATGCTTCCTGTAATGAGAAGTGATATTATGTTGAGATATAAAGATAAATGTCTAATTATCGATGCAAAAT
TTTATAAAAATACATTACACAATTATTATGATACTAAAAAGATCCATTCAACTAACCTGTATCAAATATTTACTTACGTGAAAAACCAACAATTAAATTTAAAAAAGAAAGCAATACAGGTCTCAGGAAT
GTTGTTATATGCTAAAACAGACGAAAATATTGTTTTGAACGATAAATTTCATATGAGTGGTAGTCAAATCATTATTAAGACATTAGATTTGAACTGTAATTTTACTATTATTAAAAAACAGTTGAATGGGA
TAGTTAATGATATATTTTTACTAAAATAA

> SCC*mec* from MRSA18 (ERR10804/SAMEA1317993-0002 len=549596_cov=26.4_gc=0.334

ATGAAAATCACCATTTTAGCTGTAGGGAAACTAAAAGAGAAATATTGGAAGCAAGCCATAGCAGAATATGAAAAACGTTTAGGCCCATACACCAAGATAGACATCATAGAAGTTCCAGACGAAAAAGCA
CCAGAAAATATGAGTGACAAAGAAATTGAGCAAGTAAAAGAAAAAGAAGGCCAACGAATACTAGCCAAAATCAAACCACAATCCACAGTCATTACATTAGAAATACAAGGAAAGATGCTATCTTCCGA
AGGATTGGCCCAAGAATTGAACCAACGCATGACCCAAGGGCAAAGCGACTTTGTTTTCGTCATTGGCGGATCAAACGGCCTGCACAAGGACGTCTTACAACGCAGTAACTACGCACTATCATTCAGCAAA
ATGACATTCCCACATCAAATGATGCGGGTTGTGTTAATTGAACAAGTGTACAGAGCATTTAAGATTATGCGAGGAGAAGCATATCATAAATGATGCGGTTTTTATTAATTAGTTGCTAAAAAATGAAGTAT
GCAATATTAATTATTATTAAATTTTGATATATTTAAAGAAAGATTAAGTTTAGGGTGGATGAATGAATGGCTTATCAGAGTGAATATGCATTAGAAAATGAGGTACTTCAACAACTTGAGGATTTGAACTA
TGAAAGAATAAATATACATAATATTGAATCAGAAATTAATGAATATCTCAAAGAACTAGGAGCGTTAAAACATGTCTGAACAAATAAATACTCCAAAACTGAGATTTCCAGAGTTTAGAGATGAATGGG
AAATGAAAATTATTAAAGAATTGTTTAATGTTGTTTCTGGCTCTACACCTCTTAGATCTAATACTTCTTATTATGAAAATGGAAATATACCTTGGGTAAAAACTACAGACTTAAATAATTCGTTAATTAATG
ATACTTCTGAAAAAGTAACTGATATTGCTTTAAATAACTTGAAGGTTTTACCTAAAGATACTGTACTAATAGCTATGTATGGTGGATTTAATCAAATTGGTAGAACAGGAATTTTGAATATCAAGGCAACC
ACTAACCAAGCAATTTCGGCTCTAATAAAAAAGGGTAATTATAATTCTAAGTTTTTGCAATCTTATCTCAACTTTAATGTAAAACAGTGGAGACGTTTTGCAGCAAGTAGTCGAAAAGATCCTAATATTAC
TAAAAGAGATATAGAAAAATTTAAAATACCATATACATGTCTAGAAGAACAAGAAAAGATTGGGGGTTTCTTCAGTAAAATCGACCGTCAGATTGAGTTAGAGGAAAAGAAATTAGATTTGTTAGAGCA
ACAAAAGCGTGGATATATGCAAAAAATTTTTTCACAAGAATTGCGATTTAAAGATGAGAATGGGAATAAATATCCTAATTGGCAAACAGTTAAAATTGGAAGCATTTTAAAAGAAAGAAAGGAACGTTC
AGGAGATGGAGAAATGCTTTCTGTGACAATTAATCATGGTATTGTAAAATTTGATGAGATAGATCGAAAAGATAACTCTAGTAAAGACAAAAGTAATTATAAAAAAGTTTATAAAAATGATATAGCTTAT
AATTCGATGCGCATGTGGCAGGGAGCTAGTGGAAAAGCTGAGTTTGATGGTATTGTAAGTCCAGCTTATACAGTTGTTACTCCAATTGAAAATATTAATTCAAATTTTATAGCATATTATTTTAAAACTCAT
AATATGATTCACAAATTTAGAATTAATTCACAGGGACTTACTTCAGACACTTGGAATTTAAAGTATAAACAACTAAAAGACATTAAAATTAGTATCTGTTCAAAAGAAGAACAAGATAAAATTGCTGATT
TATTAACGATATTAGACACACGTATTAAAAAGCAAAATCACAAATTAGAAATATTAAACATTAATAAAAAAGGCTTACTACAAAAAATGTTCGTTTGATACTGATAACGACCTATTATGTCAATTAAAAG

GAGAGTTGTATTTATGAACGATGAAAATTTAAACGATGAAAATTTAAACATTGTTATGTCAGTGCTAGTTGACATTTCCTTAAAAGCTAAGCAATTTAGACTTTTAAACCAAGAAAAAGAAAAGATAGAA

AAAATATTGGAATTATTGGTTTGTGAAGAACAAATTGTATTTTTAGGAAACACTGCTGCAAGTAACAAACCTTATTTGTTTCTTATCCCTAAATATTATGCATTAGAAGAAGAAATCATAGATAGAGTGAA

TACAAATTTAGATTTGTATACCATTGAAAATAATCAGTATCACAAAGTTCGAATGAATGATTCAGGTATATTAGTGAAAATTCGTATTGACCAGGATAGTTTTGATATAACTGAAAAAAGTAGATATGTCT

CAAATGAACTGGTAGTATAACAAATCTATTAACTTTCATCTATAATTAAAAAATATAAGAAATTAGAAAAAATAAATTTAAGAGCATCCCTCACCGTAAAAGTGAAGGATGCTTTATTTTTATTGTAAGTA

GTATGATATTCATTTTGATAGATATTGGTTAATAATTGCCTGTAAATCATTATTAAGTACCGTTGTTATAGAGTCATCATCCATAAAATCATCATAGATTTTATCCAATATTTCCTCATCTTTAATTGATGTA

AAATGATGTACTAGTCCTTTTACGATAGATATATAATTTTGAGATGCTAAGCAACTAGCAACTGTTCGCCAGTCTGATTGTGTAGTGTCATGGTTCATAGATAATCCTCCTTTTATTTCAATGTCCATTTTTG

ACGCGCTTTAGGATTGAGTGGATGCATGATTTCATTTGTTGCTGGGTTTATAAGCTTTTTAATTTTCTTTTTGTGAGGCTTCAACATTTCCATCACTTGCTCAACACGTTCGACGACAACAGGTCGTTTCGCA

TAAGCACCATAAGCTAGAATCACTGTGTCACTTTCGCTTATCGCTTTCATCAAGTGGATGTCTGTGTGTTTATCATAAGGTTCTTTAATATGTTTAAGGTTCTCTGGGGTTTTAATATTAGAGAATAGATTTA

CAAGATATACAGCACCATACTGTTCAGAATTCGCTAATTGGTTAAGGATAAGAACAGTTGTAAGATCGAGTGATAATACGCCATCTAAATGAGGGTACATCGTTATCACTGTACAAGCGGGTTTCTTTTCA

TCCCATGTTTTCTTGAGTAAGTAGCGGTGTTGTTCATCATCGCTAAATATGGCTTCTGTGTGTATCGTACTTTTGATTGTATTCATCATCGTCACTCCTTTTAGTATTCTTCTGGTAAAAGCATCACATAATAA

AAAGCGTCCACGTCATCTTCACGAATGACGTAGACTTTCTTAGGTAATGCGTTTTGATTTTTTTCATAGTTTGTATAGTGATATTCCAATTTGTATGCGGGTTGTTCTTGTTCATGCGTAATAGAGAGTATAT

TATCATCTTCTTGTAGTTTAAAAATGTGTAGGTAATCTGTATGAGGTTGATTGTCTCGATCTTTTACCATATTCCAAAGTAGTATTTGAAGGTCTAGAGATAGGTGTTCACTAATGCCTCTTGTGATGTATCG

ATTGATTTTCATATTATTTTACCTCGTCTTGAATTTCTTTCATGATGATAATCGCTTGGCTAATAATCGTAACAGATATTTGTGCCACTTTGATCCAATTATTCACGGTCATTCCCTCCTTGTTTTAGTAAATG

ACGTTCATCGATAATCGTACTTTTAGTATCTGTCAGGTATAGAAAGTCCATATCAAAATGATCCAAATAACCAATACTGATGAGTTGGCTATTGGCGTACATAAGGAATGGATAGATACTTAGCTCATGTA

GCTCATCATTATAGTAGGTATAAGTTTCAAGTGTAAGATGTGCAAGTGGGGTATGGTCTACAAAGCGAGTAGGAAGTATATTTTCTGCTGCGTCTTCTAACGCTTCACATTCCCATGTTTCGTTGTTAGACA

GTTGGAATAGACGAGTTATATATTGTTTGAGTTCTTGAGTGGTTGTTTTCATATCATTGCCTCCTAGATAGTGTGATAGCGATGTATTTCATATACATCACCGAGATAATATATATTTGATTTATCATTTATT

ACGAATCCCGGTGGGAATAAGAGAAAATTCCATATAAAAACCCGCTACAAACGTTGGTATAACAAGGAAATCCCGGAATCCCACTCATTTTGACGAACAATCAACTCATTATTTATAGGTATTGATGATA

TGTGTAGGTCTCTCTCTGCTTCTATATATATTATTTATTTATAAAAAATAACGGGATTTTGGGATTGTGCTTGCACAATCCTTCTGCTTCTTCGAATCTGCAAATCCCATTCATTTCCCGTTAAAAAATCATT

GTGGGATGTTCTTTAGCAATTTCAATATAAACTTCGTGTAGTTATGAAAATAATTACGACAATGACTGTTTTATTAGATAAGTGTTATTGAAATTGATAAAGAGAATTCTAAAAATGGTTAGATAAAATAA

GTGAAAGAATAGTAGTGTAGTTATTGTTTATTCAATAGTTATATATATAGTTTGTGGCAAAAATAAAGACGAAGTGCTAAGGAGCACTTCGTCAAGTGGACGGTTATTAAATAGTTGTTTGATAATTTCAT

TATTCAGTTTAAGCGTAACATAAAATTGCTTCTTATGATGTTCATGGTTTCGGATATCAATACGATCGATAACCGTCAGATAGAGTGATTTCAACTGAGATTTCTCTAATTTGTCTATATCTTTGAATATTGC

TTGTAGTACGTTGGCAATCATATCAGCATCATAATGTGGAGCTTCTGCTTGTTTATCTTGCTCTAGTTGATGGATTTGATTATTTATTTGATTCAATTCATCTTGATAGTTAAGTATTGTTGGTTTCAATACGC

TATCTAAGTCAGGTGAGTCTTCAATGGTTTTCGTTAATGTATGCATTTTGGCTTTTAGTTCTTCACATTGTGACTGTTTATAGGCAATATCATGATTCAAAGAAGATTTATCTATTTGGCTTTGTTCATTTACT

TTTTCAACCAATTGCTTCAATACCTTTTTACTTTTGATAATTTCCAATATCTGGTCCATAACATATTTTTCTAATACATCTGCTCTAACACTATTGGCTGAACATACTTTAGAACCTTTGTTTCTAAAATTGCT

ACATGAATAATATCTGATTCTTTTCTTAGTGCCATCTTTTAATGTATTGGTCGTATTACTTGCCGCCATGGCTGCGCCACATTTTGGGCACTTTACAATCCCAGTAAGTAGATTGGTCCCTTTACCATGAACT

TGTGGTTTCTTGCGACTCTCTTGGCGTTTAAACTGTACTTTATCCCATAAAGCTTTATCAATAATAGGCGCATGCTTACCATCTGCGATAATCGGTTCCTCATTGAGTCCTTTTCGTCTTTTATCGCTCCAGTG

TCTATACTTCGCAAACTGTATCTTTCCAATGTAAAAAGGGTTTGAGATGATATAAGTAATGGACGAAATACTAAACGGTTTCCCTTTCTTAGTCACATAACCTTTATGATTCAATGTGTTCGCAATCTTACG

ATAACCGTGACCTTTGGCGTATGACTCAAAAATATATTTAACAATATTTGCTTCATGTTGATTAATCATAAGCTCTTTTTTACTCTCAGGTACTTTATCATAGCCTAAAGGTAAATTACCTTGGTAATAACCT

TCAATAGCACGTTGTCTTTGGCCATTAAACACGTTTTCTACAATAACGTTTCTTTCAAATTCTGCGAAGCTCGCTAAGATTTGGAGCATCAACTTACCTGTGGAGCTCGCAATTTCTATTTTTTCAGTTAGAC

TAAAAAATTCAACATTAATCTCATACAATTCTTCGACAATATTTAATAAATCTGAGGTATTTCTAGCTAAACGAGTAGTATTATATACCATAATACCATCTAATTTACCTCCTTTAGCATCTTTTAACATAC

GTTGTAGTTCAGGACGTTGCGTTGTTTTACCTGATATACCACGATCGGTATATTCATCAACGACTTCATAACCTTGAAATTGACAATACTCTGTAAGTTGATTTAATTGACCATGGATACTGTAACCATCCG

TTTGCATTTCAGTTGATACACGTGCGTATAATCCAATACGTTTCTTTTTGAGTTGTGTCATATGACTTCATTCCTTTCAGGAGTTATTAAATGCGATTGTTCAACGATATTGAGTGGACTGTTTTTAAAGTAG

ATTCCTTGTAATTGTTTAGTTTGAGTTATTGTAATGTCATCAATCAAAGGTGCTATGATTTCTAATGTCAATTTATCTTTGACTACATGACGGATTGTTTCAGCAATTTGATTGGATGAATAATTAGAGTAAC

GTTCTTCTCTTTCAAAGCCAGCAGATAACCGTTTAAATGTTTCTATATCAATTTGATTTTGTGCTAGTTTTTCGATGAGTTGTTCTTGTGTTAGATGTGATTGTCTGTGTTTCATTTGTTGGTGTTTTAGAGCT

TTTAGTATTGTCTCATTTAATTTGTCATGAAATGATTGATTGCCACAGTATACTTTACAAGTGTTTAGGACCTCTGTTTCAATCATTTGCGCATTGATGCCTTTAAATGGACACGTATGATAGGCTTCATTCA

TATTTTTAGGACAGACATAGTAACGTAGAGAACGATGTTCTTTTTTTATCGTTAAGTTTGTTAATGTTGATTGACAGTAAGGACATTTGATTCGTCGCTTTAACTTATTTCTAGAATTGGATCGATTGTGTTG

TTTATGAATACGACGCTCTTGTGCTTCCTCAAACGTATCAACATTAATAATAGGTGGAACGATATCATTAAACGTTCCATATTTATTAATGACACGACCACAATAGTTAGGGTTCAAGAGAATATTTCTAA

CTTGATAGGGCTTACGAGAAATAAAATTCATATCTTTATCCAGCTTTTGAGAAATTTTCTTGTAACCAAGTCCTTGTAAATACCAACGATAAACGGCTTTGATTGTCGGTGCTTCTTCTGCATTTACAATAA

AGTGACCTTTGCTGTAGTGATAACCAAAAGGGGCGTGTGTTGTAATCAACTTACCTTGTTTGGCTTTTTCTCTTATCCCATTTCGTGTTTGTTCGCTGATGTTGTTCGATTCCATTTCTGCTAGACTCATGAGT

ATGTTTAAGCGAAAGCAATCAAACTCTTTAGACAAATCAAAATATCCATCGTTAACACTGATGATTGTGACGTGATGCTTTTTACAAATTTCAAAGAATTGTATAGCATTTTTGAAATTACGATGGAGCCG

ATTTAGCCGATAGCAACATAATACTTTACACTCTCCAGATGTAATCACTTCTACCATTTTTTGATAGCCTGAACGCTTTGTATGTCCACCTGTTTTTTTATCATCATAAAATGTTACATTGGACCATCCATGT

TGCTTAGCAGTGTCTATAATGAGGGATTTCTGAGTCGCTAAGCTTTGTTGTTTGAGTGTACTTTGACGTACATAAGCAATGGCTTTTTCCATTTTATACACCTCCATAGAGATAATATATGTTTATGAATGA

ATTAAAATGAAAGGTCCAACGTGTTAAAAGCACGTTGGACCGCAATCTCAGTTATCACGATTTAACTCTTCAACAACTAAGTTAGCTAATACTTCAATTAACTCATCCATTTCATTCACTCCTATACAATTT

CATCTTTAAGTTACTAAAAAGGTATTGAGAAGTGGTCATTAAAGATCAGCTAACTCATCATTTTGCTTTTTCCATAACTTAAAATTCTCTTTGTTATCCGTATTAATTTTAGGTTTAATAGGTTCGGCATCTT

TAGTTAGACCAAAAATAGACGCATGGTCTGAATCTAGCTTTAATTGGTAAAAGACAAGCGACTGTTTCTTGCCATTGTTATCTTTGACTGTTCGTTTTTTCGTAATTCGATCACGGTCAGATTCGATAAATC

CCTTATCCTTTAGTGCATTCACGACATTATTAACATCTTGGAAGTGATGCTCTGCCAACATATTTTTAAATACAGACGCAATGATTTTGACTTCGATATAATCATCTTTTAAGGCAATTAGTCCATAGTTCTC

CATCATATTCTTTAACGCTTTGTCATCAGAAAACTTACCACGGTTTTGTGCTACAAATTGCGTAATGACTTCGATCGCTTTATCTGCCAGTGATCGTTCAGAGACCGTATGAGCATGATAATCAATAAAGTA

GTCTCTTATTTTAGCGATATCAATATCTGTAGCTAAAACACGACCTAATATTTTCGCAGATGTTGTAATAACTGCATAACGCTTAAACATACGTTTACCTGTATTGTTTGTTTCATCCTTCAATTTATCTTTA

AACCAATGATGTTCATCTTTAAACCATTGAATGGCTTCATCTTCACGTTTAATAAGATAATCAGCAACGAACGGTAAAACATGCCCGTAGTTTTGTGCAACTGCTTTTTTGATATTATCAGAGTTAGTTGCA

TCTGTAGTAAATTGTTCATTAATCTCAATGGTTCTTACACGTAACCCATCGTTTTGAGCCGAATCAGTAAAAATACTGTATTCTGACGTTGAAATGACAGACGTGCCCCAATTCTTAGGCGTTTTAACTTCT

CTATGAACGTTTGAACGTTGACGACCTTGACCTTCAGCGATCGAATATAATAACCCCGTTGTATCTTTAAAAGTTGCTGATGAGAGTTCATCAAATACTAGTGGTACACCATAGTTGTTGCTTAAATAACTT

TCAAGTGCATTACGTGTAGCATTCCAACTTCTAAAGAGCGTTTCGCTTCCCTTATTAGGGTTGCCAGCAACTGATACAGCTAAAGCAGCCGCTGTTGATTTACCAGTTGATGACTGACCTGTAAAACTAAA

GATGATACCAGCAAATGTAACATCTTGTTTATGCTTCAGAAATGCTGTTACTAAGGCAGAAATTCCGAATGTGGTTGCTAACTCCAAATGAAAATGACCTTTGACCTCATTAAGATACATATTAAACCAAT

CTTCAAACGTACCTTTAGGTTCTAAATCATAAGATGTATCACAAATTGGCTCTGAAGGAGTGTTAGCATTGAATTCTTTGGTCTTATAGACATCATTTAAGGATATAATTGAACCAAATGGAGTTTCTAGTA

TGCCTACACCTTCATACAAGTGGGAAGGTGATAATAGATTGCGCGTTTGTTGCAAGGCATTACTTAAAGACGTGATGCGCTTTTCGTTAATACTATAGCCGTAATTGATTAGTAAATGTAAATTGCGAGAT

GATAAAATATCGGAATATTTTCTTGACCATTAGCATTCGAGATAACTATTTTTTCAGTACCTTTCGAAGGATCTAGAAATTTTTTTGTAATCACAATAAAACTAGCCAAAAAAACTATTCTTTTACTATCTC

CATCTTTTTTAGGTGGTATGTTTTCATACCAACCGTCTGAATTTAAAAAATAGGGTCCTATTTCAAGTATATTTTTTGACATTAGCGAACACCTCCTTTCGAAGTGTTGCTATGATTACGTGGATGTGGACCT

ATTTTTTGATATAACAAACTGCCATTTTTCGTACCTACAGTATCAAAGGGTAATCCTGGTGTGTGCTTAATCCAGTAAGCAAAACGACGTCCTACTTCACGTTGATCATTCGTGCTACAAACCCCACTATAT

TTCTTTCTTAATTCACTATAAGTGAACATGCTTCCAACTGATTGTTGAAAAGCTTTGGCAATTACAAAATTATAAAGCTTCTCCTGTGATTCACTTTTATTTCTTTTCATAACTATTATCTACCTCCTAAAATT

ATCACTTACGTGATATGGACTTTATTTTACTCTCAAAGAAAAATAGAGGTTTGAGATTTCGAAAGAGAATTTTAGGACATTGATTCTATAACGTTTAATAAATAAAAAGCCCAACGATTTAAGAATCGTTG

GGCATATTTATTGAATTAATAATATAAATTTGGTCCTTCTTTAATACTGTCTAAACGTGTCTTGTAAAACTTACTAATTATTTGAATCATTAGTTTATCGAGAAAGTCATTAGGATTATTATTAAAATTTTCT

CTTTTATAAAAGTTGCTAATGTTATATTTTTTAAGCTCAGTGAGTAAAGAGTCTTCTACTTTTTCTGTCATTTTTACATTATTTTTAATGGTTTTATGAATCAAAGCTTTCAAATTAGTATTTCTTGCATAGCC

ATTAATAAGACTCTCAAAGTGAGTTAATTTTTGTTCATGTTTGTAGGTTAAGGCAATTAGATCATTCTTTAAATTAGTAATACTATGCAGAGCAACTAATATATCATATTGAGTTGATTTTTGAATGATATT

ACTTACTAGTGTAACTAATTCATCGATTAAATCGGAAATAGCAGTAAATAATGCAAAGTGGAATGTATCTTCAATGGTCGTTAAATAATATAATTCGTTATTATTAATTAAGTTTTGTTTTTTCATAAATTC

ATTTATATTATTTTGAGACTCAATCAGGCTATCTATTGTTAAATTAACTTTACGTGACTTATTTGTCAGAATACTACGGATAAATTTAAAATACGTATCATTTTGATCATATAAGTTGAGAGAAATACCGAT

AATAGCTTGTCTGAATTGATCTAGTTCGTCAGATTGATTTAAACTATCTTTTTTATGAGTTGAAGGTTTAATACTATTAGCAATTTCAAAGTATTGAGAATTACTTGTTCGTTCTTTAATTAAACCTTCTGTC

ACTAGTTGTTTAAAGTACTTAGATTTATTTACGTTTGAGATGTTTTTCTTAACATAGGAATCTTGTTTTTCTTTGTAAATGAGATATTTTGTTAAAAAAATTACTTGTAACTACATCGTCAAAATTTTCTCTAGA

TAAATCTTGATGGATTTCTATTATTATATCTTTCAAATTTTTAAATGTATTCCAAGAAGATACGTTGTCTACAGTTTTTTCGTAATCTATTTTATATTTAGCTTTTTTTAAATTAATATTTTCTTGAATCTGTTT

TATAGTATTGTTAGCTATCTTTTTAGCAAAATCTGTTGTTTTATTGATTGTGGCTAAGAACTCGGTGAAATATGTATCTAGAATTGTTGATTCTAACACTTCTAGAATATCTACACCTTGGTCAATGATTTCA

TTTGAAAGTTCATCTATGTCCACCATACTTTGACTATCAATAGAATACGTCAGATTATCTTTAGTAAAGCCACTAGGAATTTTATCGATAAATATACCCTCTTCACTACATTTCACAGTATGTTGTTCAATAT

TTTGATTAGAGGTATTTACAAAGTAGCTTATATATATAGTTTCATTAGGTTGTGCTTCTCCCACTACATAATTTGTTTTAATTTCTTCAGGTAAAATATCTTTTGCATACTCTTTTAGTATAAATTCGGAATTA

ATTTTATGTATAAAATAATTAAAACTTTGAAACCACATTTCTATTTTCCATTTATTTTTAATCATTGCATTCACTCGCTTTCATATTTAAAGTTTGTGTATCAACAAATACTAATTTCAATGTTAAAGTTTTTC

AATAGTATTTACTATTTATTCTTTATAATATGGAGAATAAAAAACAAACGTTCTCCTTTGAGCATATATATGAAAAAAGAGTCGAATCTTTTAATGAGTGACATCAAGTTCTTAAAAAATTATATAGATAA

TAGCTTATAATTATTGTTTAAAAACTATAGTAATTTGAATTGGAACTTAATTCTAAAAACTAGTCGGGCATCTAATAATTTTTAAATTAAATCGAAATTTCCGTTTATTTACTCTTGATTACTTCAAAATATA

TTAATCTTACACATTATGAAATGATCGATAAAATGATATAAATATACAAATGAAATAATATGATAAAGTAAATGTAAACAAAATATCAGATTTAATTAACCTAGAATATATGTTATGTTAAATGTATATAA

CAATATATTCACTAGGGGTGCAATTGATTGCTGAGAGAAAGTACTTACTTTCAACCCTTGAACCTGTCGGTTAGTACCATCGTAGGAAAGTGATTTACATATTATTTGATGTTTTTCAGTAATTCACTTTTTT

GCGTAGAAAATTGAGTTATTGGAGGATTTTTTTATGGAGAAATTAAGAGTTGGTTTAGAATGGTTTTTAAATCCTGATCATATGCCATTAATCTTAGGAATAGAAAGAGGATGGTTTAAGGAACAAAATCT

AGAAATTGAGATGATCGAACCTAAAGAACATTTTGATGCTTTAGATGAAATAGAAAAAGGTACAATGGATATCGCAATTACAGAACCTATCCATTTAGTAGAAGATAAAGCATCCAACCAAAATGTTGTA

GGCTTCGCAAGATTCTTACACACCAACGGCGGTATAATGTATAAAAAAGATAAAGGTATTAAAACACCAAAAGATTTAATTGGCAAACGTCTCCAGTACCCAGGAGCTCCAGGTCTTGGGGGTATTGCTA

TTGCAAAAACAATGATTGAAGCTGATGGTGCTCAATATATAGATGGAGATATTAAACCTGTAAATAACAGTTTTTATCATACAGATGCATTATTAAATGATAAAGCAGATGCCGCAACACTTATCTTTGAA

AATTTTGAAATTTTAGAAGCCAAAAGTAAAGGATTAAATGTCGATTATTTCGCATTAAAAGATTACAATGTTCCTGACTTTTGCCAATTAATTTTTATCACTACCCCTGATAAGTTACACTTTGAAGAAGAA

AAGATCAAAACATTTATTAAAGTTATACAAAAAGCAATTCAATATATTAAAACTAATCTTGAAGATGCAATTCATATCTACTCTTCATATACAAATACAGATGTAAGCGATAATTTAAATAAATCTACAAT

ACAAGCCACAGCAAAATGTTTCACTAACGATTTATCAATGAGTTCTGATTTCTACAATGATTTACAATTATGGTTAAAAGAAACAGGTAAGATTAAACAAACAATCGAACCTAAAGAATATTTCACAAAT

CAATTACTATTTAGTAGATATGCTAACTAAATTCAAGTTTATATTCAGAGTCTCTACAATTTTTAAATTGTAAGAGGCTCTTTTACTTCTAAAATATTGCCATTCTCTTCAATATCTAAATATATTTCTTCATT

TTTTGCATTGCCGCCACCTAGTCTTTGCAATAACTGAACTACTTCATTTTTCCTTAATTGAGAAAGAACATGTTTAGAAACATAAAGGTTCTGTTGCAAAGTAAAAAAATATAGCTAACCACTAATTTATCA

TGTCAGTGTTCGCTTAACTTGCTAGCATGATGCTAATTTCGTGGCATGGCGAAAATCCGTAGATCTGAAGAGACCTGCGGTTCTTTTTATATAGAGCGTAAATACATTCAATACCTTTTAAAGTATTCTTTG

CTGTATTGATACTTTGATACCTTGTCTTTCTTACTTTAATATGACGGTGATCTTGCTCAATGAGGTTATTCAGATATTTCGATGTACAATGACAGTCAGGTTTAAGTTTAAAAGCTTTAATTACTTTAGCCAT

TGCTACCTTCGTTGAAGGTGCCTGATCTGTAATTACCTTTTGAGGTTTACCAAATTGTTTAATGAGACGTTTGATAAACGCATATGCTGAATGATTATCTCGTTGCTTACGCAACCAAATATCTAATGTATG

TCCCTCTGCATCAATGGCACGATATAAATAGCTCCATTTTCCTTTTATTTTGATGTACGTCTCATCAATACGCCATTTGTAATAAGCTTTTTTATGCTTTTTCTTCCAAATTTGATATAAAATTGGGGCATATT

CTTGAACCCAACGGTAGACCGTTGAATGATGAACGTTTACACCACGTCCCCTTAATATTTCAGATATATCACGATAACTCAATGCATATCTTAGATAGTAGCCAACGGCTACAGTGATAACATCCTTGTTA

AATTGTTTATATCTGAAATAGTTCATACAGAAGACTCCTTTTTGTTAAAATTATACTATAAATTCAACTTTGCAACAGAACCGTATTATGGAATAGAGATGTTGGTAACATTTATACAGGATCATTATACTT

AAGTTTAATTTCGTTATTACAGAACCACACATTCCAACCAGAAGAGAAAGTATGTCTATTTAGTTATGGTTCAGGAGCAGTAGGAGAAATCTTTAGTGGTTCAATCGTTAAAGGATATGACAAAGCATTAG

ATAAAGAGAAACACTTAAATATGCTAGAATCTAGAGAGCAATTATCAGTCGAAGAATACGAAACATTCTTTAACAGATTTGATAATCAAGAATTTGATTTCGAACGTGAATTGACACAAGATCCATATTC

AAAAGTATACTTATACAGTATAGAAGACCATATCAGAACATATAAGATAGAGAAATAAACTAGTGGCCGATTGTGCTTGATGAGCTTGGGACATAAATCCTAACTCGAAATAAATAAGCATATCACTAAA

CTGATTTTTTAAAGTTTACAGTGATATGCTTATTTTTTTATCTTACGATTTTGTACGTGCATGCTTGCCTAGGGGTATGGCTCGAGCCATTAGTCTCTCGCACATACTATTCCCTCAGGCGTCAGCACTTACA

AAATCGGTTGTAATTTTCATTTTTATACGCATTCTTACTGAGATTATACTAATAAGAGGAATAGTAAAAGCAATTCTAAGTAAAATTGCAGATAAGAGGTTTGTTAAAAGCAGTTCTAAGTAAAATTGCAG

ATAAGAGGTTTGTTAAAAGCAGTTCTAAGTAAAATTACAGATAAGAGGTACGTTAAAAGCAGTTCTAAGTAAAATTGCAGATAAGAGGTTTGTTAAAAGCAGTTCTAAGTAAAATTGCAGATAAGAGGT

ACGTTAAAAGCAATTCCATGCAAAATTGCTGATAAGGGGTAAGTTAAAAGCAGTTCTCAGTAAAATTGCAGATAAGAGGTACGTTAAAAGCAGTTCTAGGCAAAATTGCAGATAAGAGGTGCGTTAAAA

GCAGTTCTCAGTAAAATTGCTGATAAGGGGTAAGTTAAAAGCAATCCTAAGTAAAATTGCAGATAAGGGGTACAGAAAAACTAGACTTGATTACAAAATGGAGCTTGGGACATAAATGATTTTTTAAAA

ATGAGATGAGACGTAGATTAACTCCATAATCAATACGAATCTATCGACTTCTTTATTTATGATATTCATCTCTTTTTAATGGAAATAAAAGTGCGATTAATGTGATAATACAGTTACGTTAATTAAAAAAAT

AAAAATGCAAGGAGAGGTAATATGCTAACTGTATATGGACATAGAGGATTACCTAGTAAAGCTCCGGAAAATACAATTGCATCATTTAAAGCTGCTTCAGAAGTAGAAGGTATAAACTGGTTGGAGTTA

GATGTTGCAATTACAAAAGATGAACAACTGATTATCATTCATGATGATTATTTAGAACGGACTACAAATATGTCCGGGGAAATAACTGAATTGAATTATGATGAAATTAAAGATGCTTCTGCAGGATCTTG

GTTTGGTGAAAAATTCAAAGATGAACATTTGCCAACTTTCGATGATGTAGTAAAAATAGCAAATGAATATAATATGAATTTAAATGTAGAATTAAAAGGTATTACTGGACCGAATGGACTAGCACTTTCT

AAAAGTATGGTTAAGCAAGTGGAAGAACAATTAACAAACTTAAATCAGAATCAAGAAGTGCTCATTTCAAGCTTTAATGTTGTGCTTGTTAAACTTGCAGAAGAAATCATGCCACAATATAACAGAGCAG

TTATATTCCATACAACTTCGTTTCGTGAAGACTGGAGAACACTTTTAGATTACTGTAATGCTAAAATAGTAAACACTGAAGATGCCAAACTTACTAAAGCAAAAGTAAAAATGGTAAAAGAAGCGGGTTA

TGAATTGAACGTATGGACTGTAAACAAACCAGCACGTGCAAACCAACTTGCTAATTGGGGAGTTGATGGTATCTTTACAGACAATGCAGATAAAATGGTGCATTTGTCTCAATAGAAAGTTAGAGGTGAG

TCTTACGTTTCAGTGACGGTAGACTTACCTTTAACATGTTACATACTAAAAAATTAATTTGAATAAGAAAGAGAGACATATATGAAATACGATGATTTTATAGTAGGAGAAACATTCAAAACAAAAAGCC

TTCATATTACAGAAGAAGAAATTATCCAATTTGCAACAACTTTTGATCCTCAATATATGCATATAGATAAAGAAAAAGCAGAACAAAGTAGATTTAAAGGTATCATTGCATCTGGCATGCATACACTTTCA

ATATCATTTAAATTATGGGTAGAAGAAGGTAAATACGGAGAAGAAGTTGTAGCAGGAACACAAATGAATAACGTTAAATTTATTAAACCTGTATACCCAGGTAATACATTGTACGTTATCGCTGAAATTA

CAAATAAGAAATCCATAAAAAAAGAAAATGGACTCGTTACAGTGTCACTTTCAACATACAATGAAAATGAAGAAATTGTATTTAAGGGAGAAGTAACAGCACTTATTAATAATTCATAATAAAACAGTG

AAGCAACCATCGTTACGGATTGCTTCACTGTTTTGTTATTCATCTATATCGTATTTTTTATTACCGTTCTCATATAGCTCATCATACACTTTACCTGAGATTTTGGCATTGTAGCTAGCCATTCCTTTATCTTG

TACATCTTTAACATTAATAGCCATCATCATGTTTGGATTATCTTTATCATATGATATAAACCACCCAATTTGTCTGCCAGTTTCTCCTTGTTTCATTTTGAGTTCTGCAGTACCGGATTTGCCAATTAAGTTTG

CATAAGATCTATAAATATCTTCTTTATGTGTTTTATTTACGACTTGTTGCATACCATCAGTTAATAGATTGATATTTTCTTTGGAAATAATATTTTTCTTCCAAACTTTGTTTTTCGTGTCTTTTAATAAGTGA

GGTGCGTTAATATTGCCATTATTTTCTAATGCGCTATAGATTGAAAGGATCTGTACTGGGTTAATCAGTATTTCACCTTGTCCGTAACCTGAATCAGCTAATAATATTTCATTATCTAAATTTTTGTTTGAAA

TTTGAGCATTATAAAATGGATAATCACTTGGTATATCTTCACCAACACCTAGTTTTTTCATGCCTTTTTCAAATTTCTTACTGCCTAATTCGAGTGCTACTCTAGCAAAGAAAATGTTATCTGATGATTCTAT

TGCTTGTTTTAAGTCGATATTACCATTTACCACTTCATATCTTGTAACGTTGTAACCACCCCAAGATTTATCTTTTTGCCAACCTTTACCATCGATTTTATAACTTGTTTTATCGTCTAATGTTTTGTTATTTAA

CCCAATCATTGCTGTTAATATTTTTTGAGTTGAACCTGGTGAAGTTGTAATCTGGAACTTGTTGAGCAGAGGTTCTTTTTTATCTTCGGTTAATTTATTATATTCTTCGTTACTCATGCCATACATAAATGGA

TAGACGTCATATGAAGGTGTGCTTACAAGTGCTAATAATTCACCTGTTTGAGGGTGGATAGCAGTACCTGAGCCATAATCATTTTTCATGTTGTTATAAATACTCTTTTGAACTTTAGCATCAATAGTTAGT

TGAATATCTTTGCCATCTTTTTTCTTTTTCTCTATTAATGTATGTGCGATTGTATTGCTATTATCGTCAACGATTGTGACACGATAGCCATCTTCATGTTGGAGCTTTTTATCGTAAAGTTTTTCGAGTCCCTTT

TTACCAATAACTGCATCATCTTTATAGCCTTTATATTCTTTTTGTTTTAATTCTTCAGAGTTAATGGGACCAACATAACCTAATAGATGTGAAGTCGCTTTTCCTAGAGGATAGTTACGACTTTCTGTTTCAT

TAGTTGTAAGATGAAATTTTTTTGCGAAATCACTTAAATATTCATCCATTTTTTTAACGGTTTTAAGTGGAACGAAGGTATCATCTTGTACCCAATTTTGATCCATTTGTTGTTTGATATAGTCTTCAGAAAT

ACTTAGTTCTTTAGCGATTGCTTTATAATCTTTTTTAGATACATTCTTTGGAACGATGCCTATCTCATATGCTGTTCCTGTATTGGCCAATTCCACATTGTTTCGGTCTAAAATTTTACCACGTTCTGATTTTA

AATTTTCAATATGTATGCTTTGGTCTTTCTGCATTCCTGGAATAATGACGCTATGATCCCAATCTAACTTCCACATACCATCTTCTTTAACAAAATTAAATTGAACGTTGCGATCAATGTTACCGTAGTTTGT

TTTAATTTTATATTGAGCATCTACTCGTTTTTTATTTTTAGATACTTTTTTTATTTTACGATCCTGAATGTTTATATCTTTAACGCCTAAACTATTATATATTTTTATCGGACGTTCAGTCATTTCTACTTCACC

ATTATCGCTTTTAGAAATATAACTGCTATCTTTATAAACTTGTTTGAAATTTTTATCTTCAATTGCATCAATAGTATTATTAATTTCTTTATCTTTTGAAGCATAAAAATATATACCAAACCCGACAACTACA

ACTATTAAAATAAGTGGAACAATTTTTATCTTTTTCATCAATATCCTCCTTATATAAGACTACATTTGTAATATATTACAAATGTAGTATTTATGTCAAAATAATGTTATAATTTTTGTGATATGGAGGTGTA

GAAGGTGTTATCATCTTTTTTAATGTTAAGTATAATCAGTTCATTGCTCACGATATGTGTAATTTTTTTAGTGAGAATGCTCTATATAAAATATACTCAAAATATTATGTCACATAAGATTTGGTTATTAGTG

CTCGTCTCCACGTTAATTCCATTAATACCATTTTACAAAATATCGAATTTTACATTTTCAAAAGATATGATGAATCGAAATGTATCTGACACGACTTCTTCGGTTAGTCATATGTTAGATGGTCAACAATCA

TCTGTTACGAAAGACTTAGCAATTAATGTTAATCAGTTTGAGACCTCAAATATAACGTATATGATTCTTTTGATATGGGTATTTGGTAGTTTGTTGTGCTTATTTTATATGATTAAGGCATTCCGACAAATTG

ATGTTATTAAAAGTTCGTCATTGGAATCGTCATATCTTAATGAACGACTTAAAGTATGTCAAAGTAAGATGCAGTTCTACAAAAAGCATATAACAATTAGTTATAGTTCAAACATTGATAATCCGATGGTA

TTTGGTTTAGTGAAATCCCAAATTGTACTACCAACTGTCGTAGTCGAAACCATGAATGACAAAGAAATTGAATATATTATTCTACATGAACTATCACATGTGAAAAGTCATGACTTAATATTCAACCAGCT

TTATGTTGTTTTTAAAATGATATTCTGGTTTAATCCTGCACTATATATAAGTAAAACAATGATGGACAATGACTGTGAAAAAGTATGTGATAGAAACGTTTTAAAAATTTTGAATCGCCATGAACATATAC

GTTATGGTGAATCGATATTAAAATGCTCTATTTTAAAATCTCAGCACATAAATAATGTGGCAGCACAATATTTACTAGGTTTTAATTCAAATATTAAAGAACGTGTTAAGTATATTGCACTTTATGATTCAA

TGCCTAAACCTAATCGAAACAAGCGTATTGTTGCGTATATTGTATGTAGTATATCGAGCTTCACATGAAACAGCTAAAGAAGCTTTGGGCGATAAAGAGTTAAGAGCCATTGCACATGAGTTAACTAAAA

CAGTTAAGGATAACATGAGTGTTGATTGGTCTAAACGAGACAGTGCTAAAGCTAAAATGAGAGTTCAAGTTAGACGCCTATTAAAGAAATATGGCTATCCACCAGATCTTCAAAAAATGGCTGTGGAACA

AGTTGTAGAGCAAGCAGAATTAATGGCAAGTCAGCAATAAAAAAATAAATCATAATGAGTCCGGGACATAAAGTTCTTGGATAAGTGAAAAAAGACAATTTCTATTGAAATAATATAGAAATTGTCTTTT

TTATAAATTTTTTGATTATTTTCAGCTCGTTGAGCTACTACTTTTCTTATATTAAGTGCCATTAATACAAAACCAAGTTCTCTTTTGACTTTATTGAGTCCTCGGACAGACATCCGAGTGAAACCCAAAATAG

CCTTCATAAATCCAAAAACAGGTTCCACATCAATTTTTCTTTGACTGTAGATATTTTTTGTTTCTGGTTCTGAAAGCTTTTTGTTAATTTGGGATTTAAAATATTCCCAGTTATAATTCTTCATTATTTTTTTGT

TTGTTTTTGAATTGAAGTTCATACATTGATTTTTCAGAGGACATTCTGAACAATCATCACATTCATATAATTTGAAGTCTCGCTTATAACCATACTTATCATGACGATAGGCATATCTTTTAAAACCTAGCCG

TTTATTATTCGGACAAATGAATTCGTCATTAATTTCGTCATAGTTCCAATTTTGAGTATTAAAGATGTCACTTTTATATTTTTTAGTTTTATCTTTTATAAACATTCCATATGTTATGAGTGGCGTTCGATTAA

AGTCATCTATAATTGCCTTATAATTTGATTCACTACCATAACCTGCATCAGCTACAATATATTCAGGTAAATGACCGTAGGTCTCTTGAATTGAATTTAAAAATGGAATCATCGTTCTAGTATCCGTTGGAT
TTTGATACACATTATAAGATAAAACAAATTGGGAATTTGTTGCTATTTGTAAATTATACCCTGGCTTAAGTTGTCCATTTTTCATGTGATCTTCTTTCATTCTCATAAATGTCGCATCATAATCTGTCTTAGA
ATAACTATTTCTATCCTTTAAAATAGATTTTTGAAATTCGTATCGATACTTTCGCTCAAAATAATCATTGATTTGCTTTTTGTATTTTTTGATTTTAGTTCTTTTGAGACGTATTTGTTTTCTTGTTTTAGTACA
TTTTTCATTGTTGATATGTTGGTTTAAATCTTCGATTTCTTTATCTAAGTGACTACCAATCAAATCTATTTCTTCTTTTGTTAATTCATTATCATGATCTTCTTTAATTTCCGGTATGATTTTATTGGTTACCAA
TTCATGGTAGAGGGCTTTAGAATCCTCATTCATCTTTGATTCATGGTTTTGAATACTCTTTTTCCATACAAATGTATATCGATTGGCATTTGCTTCAATTTTTGTACCATCAATAAAAATAGCTTTATCATCTA
TAAGATTTTGTTTTACACACTGACTGTAAAATTGAATAAATAAAGATTCTAATAAAGCATCTACTTTTGGATTTACTCTAAATTGATTAATTGTTTTATAAGAAGGTTTTTGATTTTGTGATAGCCACATCAT
TCGGATGCTATCATTAAGCATTTTTTCTATTTTACGACCTGAGAATACAGATTGTGTGTAGGCATATAGAATCACTTTTAACATCATTTTAGGATGGTACGAAGTTGCACCACGGTGATGTCTGAATTCGTC
GAATTCATTGTCAGGAATTGTTTCAACAATATCATTTACAGTAAAACGATGTTGATTTGTTTTGTTTCCATATTGACCTCCATGTATTTGCTATGATTTCAAAATCCATTTTTGACGTGCCTTAGGGTTGAGT
GGATGCATAATTTCATTTGTTACTGGATTGATGAGCTTTTTTACTTTCTTTTTATGAGGTTTTAACATTTCCATCACTTGTTCGACACGGTCGATAACAACTGGTCGCTTCGCATAGGCACCATAAGCAAGAA
TCACTGTGTCACTTTCACTAATCGCTTTCATCAAATGAATATCAGTGTGCTCATCGTATGGATTTTTGATATGTTTGAGGTTTTCGGGTGTTCTAATATTTGAAAATAGATTTACAAGATATACAGCACCATA
TTGTTCAGTATTAGCTAATTGATTGAGGATAAGAACAGTTGTGAGATCGAGTGATAATACACCATCTAAATGAGGGTACATCGTTATCACTGTACAAGCGGGTTTCTTTTCATCCCATGTTTTCTTGAGTAA
ATAGCGGTGTTGTTCATCATCGCTAAATATGGCTTCTGTGTTTATCGTACTTTTGATTGTATTCATCATCGTCACTCCTTTTAGTATTCTTCTGGTAAAAGCATCACATAATAAAAAACATCTACATCATCTT
CTCGGATGACGTAGACTTTCTTAGGTAATGCATTTTGATTTTTTATATAGTTTGTATAGTGATATTCCAATTTGTACGTGGGTTGTTCTTGCTCATGTATGATTGAAAGTATATTCTCATCTTCTTTCAGTCTA
AAAATGTGTAGGTAATCTGTATCAGGTTGATTATCTCTTTCTTTTACCATGTTCCAAAGTAAGATTTGAAGATCTAGAGATAGGTGTTCACTAATGCCTCTTGTGATATATCGATTGATGTTCATACTATTTT
CCTCCATTTTGCTTTTCTTTCATGATGTCAATCACTTCGTTAATGACTGTAACAGATATTTGTGCCACTTTGATCCAATTATTCATGGTCCTGACCTCCTTGTTTTAGTAAATGACGTTCATCAATAATGATAT
TTTGAGTATCTGTAAGGTACAGAAAGTCCATATCAAAATGATCCAGATAACCAATACTGATGAGTTGGTTATTGGCATACATTAGAAATGGATAGATACTTAGCTCATGTAGCTCATCATTATAGTAGGTA
TAAGTCTCGAGTGTGAGTTGTACCAGTGGAGAATCATTAATAAAACGTTCCGGTAGAATATTTTCTGCTGTTTCCTCCAGCGCTTCACATTCCCAAGCTTCGTTATTAGATAGTTGGAATAAGTGGGTCATA
TATTGTTTGAGTTCTTGAGTGATGGTTTTCATATTATTGCCTCCTAGATAGTGTAATAGTGATGTAGTTCATATACATCATTGAGATAATATATATTTGATTTGTCATTTATTACGAATCCCGGTGGGAATAA
GAGAAAATTCCATATGAAAAACCGCTTCAAACCTTGGTATGACAAGGAAATCCCGAAATTCCGCCTGTTTTGACGAACAATCAACTCATTATTTATAAGTATTGATGATAGTGGCGGGTCTTTACTTCCTT
ATATATATTATTTATTTATAAAAAATAACGGGATTTTGGGATTGTGCTTGCACAATCCTTCTGCTTCTTTGAATCTGCAAATCCCATTCCTTTCCCGTTAAAAAATCATTGTGGGATGTTCTTTAGCAATTTC
AATATAAGCATCGTGTAGTTATGAAAAAAATTACGATAATGACTATTTCATTAGATAAGTGTTATTGAAATTGATAAAGAGAATTCTTAAACTTACTTAGAAACTTTAAAGAAAGAAAAAACGAAGTGCT
ACAAGGCACTTCGTTAATATTACAATTGGTATTATTAATCTTCTTCACTAGAAATGTTGATATCGAATATACAGATTTAGAGCTATCTACATTTCCACGTGGATTATTTGGATTATAACCTACGAACGCTTG
TTCATCTTTAGATAAATTATCTAATTTAATATACTTAATATTTTTATTGCAGCTTTTATTGTTCTTCCTCACTGTCCTCTTCGCCATATATTTTTAATCTTCCTTGAGACTCTAATAAATCTGTAGTTACTGTTC
GTTCGCTATCTTCAATTTTTCCATCTCCATCTTCATCAAATTGCTCCGGATTTTGAGCTGCTTCATCAAGAGATCTATTTACAGCATCTTGATTAAAATTATTTTTTCCAGGTGGTTCTTCTTGTCCCTCTGTTT
GCTGAGTAGTGTTATTTTCACTAGTGTTATTAGTTTGAGCAGTGTTTTGTTCATTGTTATTATTGTTTTGCATAGTATTTTCTTGTGTATTATTGTTCTGAGAATTATCTTGTTGAGTTGTGTTATTATTTGCAG
ATTCATCGTCAGAAGAGATGTTACTTTCACTATTGTTCATTTCTTGTGAATTACTATTTTCATCTTTATTAGTTTTATTTTCTTTTTTATCTTTTTTTGTACTTTGATGAGTAATATTTTGGTCTTCTTTATTTTC
ATTATGATTAAAAGACTCTTTAGCAAAATTTACTACAAATAGTGCTAAACCAATAAATATGATTGATAGTATTGCAATCAATAGAATATTAATAATTTTTTTCATTCATTTTTCCCCTTAATTTGTAATATTT
AAAATTTAATAAATTAATTTTATCTAATTTTTATAATTGCATCTACTATATTTTTAATTATTTATTATTTTAGTTAATATTTATACTCACTTCTTTTATTAAATAGTAAGATATAATGTTTGGGATAAAAATAA

AGACGAAGTGCTAAGGAGCACTTCGTCAAGTGGATGATTATTAAATAGTTGTTTGATAATATCATTATTTAGTTTGAGCGTAACATAAAATTGCTTCTTATGCTGTTCATCTCTTCGGATATCAATACGATC

GATGACTGTTAGATATAGTGATTTCAATTGAGATTTCTCTAATTTGTCTATATCTTTGAATATTGTTTGTAGTATATTGGCAATCATATCAGCATCATAATGTGGAGCTTCCACTTGTTTATCTTGTTCTAGTT

GATGGATTTGATTGTTTATTTGATTCAACTCATCTTGATAGCTAAGTATTGTTGGTTTCAACACACTATCTAAGTCAGGTGAGTCTTCGATCGTTTTCGTTAGTGTATGCATTTTGGCTTTGATTTCTTCACAT

TGTGACTGTTTATAAGCGATGTCATGATTCAAAGAAGAGACATCTATTTGACTTCTTTCATTTACTTTTTCAACCAATTGTTTCATTACCTTTTTACTTTTGATAATCTCCAATATTTGGTTCATAACATATTT

TTCCAATATATCTGCTCTAACACTATTGGCTGAACAAACTTTAGAACCTTTGTTTCTAAAATTACTACATGAGTAATATCTGATTCTTTTCTTAGTGCCATCTTTTAATGTATTGGTTGTATTACTTGCCGCCA

TTGCTGCACCACATTTCGGACATTTTACAATGCCAGTCAGTAGGTTTGTCCCTTTACCATGGACTTGTGGTTTCTTGCGACTCTCCTGGCGTTTAAACTGTACTTTATCCCATAAGTTTCTATCAATAATAGG

TGCATGCTTACCGTCAGCGATAATAGGTTCCTCATTGAGTCCTTTTCGTCTCTTATCGCTCCAGTGTCTATACTTCGCAAACTGTATTTTTCCAATGTAAAAAGGGTTTGAGATGATGTAGGTAATGGATGA

AATACTAAACGGTTTACCTTTCTTAGTTACATAACCTTTGTGATTTAATGCATTCGCAATCTTACGATAACCATGACCTTTAGCGTATGACTCAAAAATATATTTAACAATATTTGCTTCATGTTGATTGATC

ATGAGCTCTTTTTTACTGTCAGGTACTTTATCATAGCCTAGAGGCAAATTACCTTGGTAATAACCTTCAATGGCACGTTGTCTTTGACCATTAAACACGTTTTCTACAATAACGTTTCTTTCAAATTCTGCGA

AGCTCGCTAAGATTTGGAGCATCAATTTACCTGTGGAGCTCGCAATTTCTATTTTTTCAGTTAGACTAAAAAATTCAACATTAATCTCATACAATTCTTCGACAATATTTAATAAATCTGAGGTATTTCTAG

CTAAACGAGTAGTATTATATACCATAATACCATCTAATTTACCTTCTTTAGCATCTTTTAACATACGTTGTAATTCAGGACGTTGTGTTGTTTTACCTGATATACCACGATCGGTATATTCATCAACGACTTC

ATAACCTTGAAATTGACAATACTCTGTAAGTTGATTTAATTGACCATGAATACTGTAACCATCCGTTTGCATTTCAGTTGATACACGTGCATATAATCCAATACGTTTCTTTTTGAGTTGTTTCATACGACTT

CATTCCTTTCAGATATTATTAAGTGTGATTGTTCAACGATATTGAGTGGACTATTTTTAAAGTAGATTCCTTGTAATTGTTTAGTTTGAGTTATGTTGATGCAATCGATAAAAGGTGCTATGGTTTCTAATGT

CAATTTATCTTTGATTATATGACGGATTGTTTTGGTAATTTGTTTATGTGAATAATTAGAGTAACGTTCTTCACTTTCAGAGCCAGCAGATAAATGTTTAAACGTCTCGATATCAATTTGATTTTGTGCCAGT

TTTTCGATGAGTTGTTCTTGTGTTAGATGTGATTGTCTGTGTTTCATTTGTTGGTGTTTTAGAGCTTTTAGTATTGTCTCATTTAATTTGTCATGAAATGACTGATTGTCACAATATGCTTTACAAGTGTTGAG

GACCTCTGTTTCAATCATTTGCGCATTGATACCTTTAAATGGACACGTATGATAGGCTTCATTCATATTTTTAGGACAAACGTAGTAACGTAGAGAGTGTTTCTCTTTTTTTATCGTTAAGTTTGTTAATGTT

GATTGACAGTAAGGACATTTGATTCGTCGCTTTAGCTTATTTCTAGAATTGGATCGATTGTGTTGTTTGTGAATACGACGCTCTTGTGCTTCTTCAAACGTATCAACATCAATAATAGATGGAACGATATCA

TTAAACGTTCCATATTTATTAATGACACGACCACAATAGTTAGGGTTCAAGAGAATATTTCGAACTTGATAGGGCTTACGAGGAATAAGCTTAGGATGGTTATCTAAATGTTGGGAAATCTTTTTGTAGCC

TATACCCTGTAAGTACCAGCGATAGACCGCTTTAACTGTATACGCTTCTTCTTCATGTACAATAAAATGACTTTGTCTATAACGATAGCCAAACGGAGCATGAGTTGTGATTAGCTTACCTTGTTTGGCTTT

TTCTCTAATCCCATTTTTTGTTTGTTCGCTGATATTGTTTGATTCCATTTCCGCTAGACTCATAAGTATGTTCAAGCGAAAACAATCAAACTCTTTAGCTAAGTTAAAATAGCCATCGTTAACACTGATAATC

GTGATATGATGTGTTTTACAAATTTCAAAGAATTGTATGGCATTTTTCAAATTACGATGGAGCCGATTTAGGCGATAGCAACACAATACTTTACATTTTCCAGAACGAATCGCTTCTACCATTTTTTGATAG

CCCGAACGTTTTGTATGTCGACCTGTTTTCTTATCATCATAAAACGTCACATTAGACCATCCATATTGCTTAGCGGTATCCATAATGAGCGATTTTTGAGTAGCTAAGCTTTGTTGTTTGAGTGTACTTTGAC

GTACATAAGCAATCGCTTCTTCCATGTTATACACCTCCAAAAAGATAATATATATTTGTGAGTAAATTAGAATGAAAGGTCCAACGTGCTTTTAACACGTTGGACCGCAATGATTAATTATCGTTCTGTAA

CTCTTCAACGATTAAATCAGCTAGTAATTCAATCAATTCATCCATTTTATCTACTCCTGTACAATTGCATCTTTAAGTTATTAAAAAGGTATTGAGAAGTGGTCATTTAAAGATCAGCTAACTCATCATTTTG

CTTTTTCCATAACTTAAAATTCTCTTTGTTATCCGTATTAATTTTAGTTTTAACAGGTTCAGCATCTTTAGTTAGACCAAAAATAGACGCATATTCTGAATCTAGCTTTAAATGGTAAAAGACAAGTGACTGT

TTTTTGCCATTGTCATCTTTGACTGTTCGTTTTGTTGTAATTCGATCATGGTCAGATTCGATAAATCCTTTATCCCTTAGTGCATTAACAACATTATTGACATCTTGGAAGTGATTCTCTATCAACATATCTTT

AAATACAGATGCAATGATTTTGACTTCGATATAATCATCTTTTAAGGCAATTAGTCCATGGTTCTCAATCATATTCTTTAACGCTGTATCATCAGAAAACTTACCACGGTTTTGTGCTACAAATTGCGTAAT

GACTTCGATCGCTTTATCTGCCAGTGAGCGTTCAGAGACTGTATGACCATGATAATCAATAAAGTAGTCTCTTATTTTAGCGATATCAATATCTGTAGCTAAAACACGACTTAGAATCTTGGCCGAAGTAG

TGATAGCCGCATAGCGCTTAAACATACGAATACCAGTATTATTTTTTTCATCCTTCAATTTATCTTTAAACCAATGATGCTCATCTTGAAACCATTGAATGACTTCATCTTCACGGTTGATAAGATATTTAGC
TACTAACGGTAAAACATGACCATAGTTTAGTGCCACAGATTTTTTAATATTGTCAGAATTTGTTGCATCTTTAGTAAACCGTTCATTAATTTCAATAGTTCTCACGCGTAAACCATCATTTTGAGCAGAATC
AGTAAAGATACTGTATTCAGAGGTTGAAATCACAGAAGTGCCCCAATTCTTAGGCGTTTTAACTTCTCCATGTACATTGGAACGTTGACGCCCCTGACCTTCAGCGATAGAGTACAATAACCCTGTTGTAT
CTTTAAAAGTTGCTGAGGAGAGCTCGTCAAATACCATAGGTATGCCATAATTGTTACTTAAGTAACCTTCAAGTGCATTACGTGTGGCATTCCAACCTCGAAAAAGTGTTTGATTACCTTTAGTTGGATTTC
CAGCGACGGATACTGCTAAAGCTGCTGCTGTTGATTTACCAGTTGATGACTGGCCTGTAAAACTAAAGATAATTCCTGCAAATTCGATTTCACTTTTGTACTTCAGAAAGCTTGTCACTAAGGCAGAAATT
CCAAATACGACCGCTAACTCTAGAAGAAGATGACCTTTGACTTCGTTAATATACATATTTAACCAATCTTTAAATGTACCTTTAGGCTCTAATGGATAAGCACTATCGACAATAGGATCTAAAGATGATAA
TTTATCATATTGTTTAGATGTATAAATGGTATCTATCATTACAATATAACCGTGGGGTGTTTCTATAATGCCCGAGCCATCATATAAATCAGAAGTAGGCAGTTCATCGCGCATTAACTGTAGTGCATAGCT
CAAATCTTTTATATAACTTTCATTGATACTGTGACCATATTTAATCAAGGAAGGTAACTTGCGAGTTGTTAAAATATCAGATTCAAATGTATGTTCTTTATCCTTACCGTTAGAAATAATTATTTTTTCTGTA
TTTTCTAAGGCATGCCAAAATTTAGCTTCAACAGCCATACAACTAGACATAGTCACTACTTTTTTCTCATCTCCATCTTTTTTAGGTGGGATAGTTTTATGCCAACCCATTGTATCTAAATGGTATAGGCCTA
ATCTAAAAATGTCATGATGACTCATTAGCGAACACCTCCTTTCGAAGGGTTGCTGTTATTGAGTGGATTAGGACCTGTTTTTTTATACAATAAACTACCATTTCTCTTACCAATTATAATAAAAGGAATGCT
AGGTGTATGTTTTACGAAGTATGCGAACCAACGTCCTACCTCGCGTTGAACATCTCTAGAACAGTTAATCCCTGACTTTTTAGTTAAATCCCCAATCGTAAAATCAGTTCCTTCCTGTAAATTCCATGCAAT
AGCTAAATCTACATTTTTTAAATTTATAAATTCATGACTTTTATAAGTCATAAAAATCACTCCTTACTTAATTGCTAGTAAAGTGACTAATTACTTAATCAACTTTACACTTGAATTATTGCAAGGATCAGA
ATTTTAAGTCATAGAGAGTTTTAATAACACTTTTTATCGATTTAATACATTAATGTATTAACTCAGTAAAGTATAAAAGGAGAGATAAAATGAATAATTGGAAAAATGAGTTGTTCGAAAGATTAAGAGAT
ATTATTGAACTTGAGATTGCAATACATCTAATGATCAGGAAACATGAACGTAATTCGATAGATAAATATAAGAACATTAAGGAGTTTTACCATAATCTAATACAGAAGGATATAGTTATTTATGATTATGA
GACATTTCATCGATTCTATAAAACAAGCAATATTCACAATTACTTACGGGTAAAACTAAAGCCAAAAAAAGCTTGGGAAGAAGAAAAAGTCATTCATCCAGAATCTTTTAAAAGTATCTTAGCAATACGT
TTAGGTGAAACAAAATTGATGGCTGGGACTAAATACATTGACCCCAATATTATTAAAAGCTTACGCCAAACTTATAAACAACTAAATTCTAATATTGAACAAATTTTAGATAAAATAAGTAAGAATACAT
TTGACATAAAATTGGAGCCAAAGTTGATTGCTTATAAAAATTTATATGAATTATATAGTTATTTTATAGATGAAATAGATAACTATAGCAATGACTTTGAACAATTACGAGCACTTCTATATTTAGAATTA
GATTACTCATTTGTACTTTATAATGAGTTACTAAGGATGATACATTTAATCAAAAAGTATAAACTAGCTACTAGTAGCAAGATAATTGAAAAAGAAATAGGCGAAATTAGTATGAAGTTAGCAAATATAG
AATTTCCGTGGCTAAGATTAGTAGTTCTAAGAGCTTTTATTGATAATATAAAAAAATATCATAATATAAATTCATTGATTAACGATATTAGTCATATTACTAATTATATATTATTCGAAATCGAACAGTGGG
TTAAACGAGCAGAAATTGAAGAAGGAATTAGAGATTTAATGAATGTGAAATATGGTAGGATCCCTTATCTAGATGCAATTATAGAAAACAGATATAAAAGAAATAGTATTAAAGAATATTTTATCTTACT
CAAACATGAATTTGAAATTAGATGGTTAAGAGGTCGAGACTTTAATGATCAAGATAGGAATAAACTTCATAGAGCTATTAAAGATATATCTCTTAATCTTATTGGATACAATAACTAACTTAAACACATTA
TCTACAAGTAAAAACTTGTGAAAAGAACATGTGTTCTTAATTGGTGTATAATTAGTTTAAATTAATCTTGGGGTGATGTAATGAATGAGAGTAATCAAATTATATTCGATCATATAACAAAGGCTATTGAA
AATATGAAAAATAAAGGCTGGGAAGCAAGGGATGGGCAAGAAACTTTAATGTATGATGTATTTGATGCTTATGATTCAAGAGAAAATTTAATAGTAGAAGCGCAAGTAGGTATAGGAAAATCATTTGGA
TATTTAATTCCTGGTATATTGATTTCAAAAAATACTAAAAAGCCTTTAATTGTAACGACTTCCTCAATTCAATTAACTGAGCAACTAGTCAATGATATACAGAAAGTTGAAGAAATTTTACATATATCTGTT
GATTGTATAGTAGGTAAAGGTGTAACTAACTATCCTTGTTTTAAAGAAATTCACCGTAAAAATTTAAGTAAATTAAATTTAATGGAAGCTTTAGATATAGCAAATAAAGGTCTAACAAAACAAACTGTAC
GTGAGACTAATTTAAAATGGAATCAAATATCTACCAATAATTGTATTATGAGTAAATGTCATTATAAAAATGAATGCGCTTATTTTAAAATGAGAAATAAGTTAAAAGAGGGAAATAGATATATTAGACT
CAATGAATATAAGCCGAAAGTCATAATCGTTAATCAAGATATGTTAATGATGAATTTTAAAAAGTTAACCTTTGGAAAAGAGAGTTTAATCTATGATGATCCTTGTATGCTAATCGTAGATGAAGTTCACA
ACTTAGAAGAGAAACAACGTGCTAACATGACTAAAACTATTAACTCTAAAACTGTGATAAATAAAATAAAAGAAGGCGCAAACCGAGTTGGTAGTAGATCTCGGTATTTAAAAAAATATAAAAATGATTG

AAGATTGGTTTAATCTTCAAAAGGATAGTGCCAAAAGTGAGATATACAAAAATGGTAATTGTTTATCTACTGGACGAGTTGATATAAAACCTGTTAGTCATCATCAAATGGCCAAATTAATTAGTATGACA
AAGGAAATAGTCGAAGAATTCGATGTTAATAATATTGTTGTATTTAAACAATCTTCGTTAGTTAGTAATGAACAATTAGAAATAGCTAATAATCTGTTAACATTATTTAAAAATCTTCAAAATAACAGCGA
CAAATATATATTTTGGACTGAAATAACTAAGCAAGACCAAATTGATATTTCATTTTGTCCCAATAATATTGCTGAAACACTTAGAAAAACAGTTTTTAGTACAAGTTACCCAGTTGTATGTCTTTCTGCAAC
AATTACTAATAAAACTAATAATGAAAATAGCTATGAGTACATTAAAGAAATAATTGGTTTTAAAGGCTATGAGGAAGATATTAAGTATAATGATTTTCCGTACAAGGAAAGCAGGCTCTACATACCTCCA
AACTTACCAAAATTTGATAAACGTGATGTTAAATATTATGAAGAAATAGGTAAACATATTTTTGAATTGGCAAGCCAAAATAAGGGAGGCACTTTGATATTGTTTACTGCTAAAGATGACATTGACGGTGT
ATATAATGATTTATCAAAAAGGAAATTTAATAAAACTATCTATGTAGATGATGGTAGTAAAAGTCAAAATGAAATTATTGAATCATTTAAAAAAACTAAAGGTGTAATATTGGGTACGGGAGTATTTTGG
GAAGGAATAGACTTAAAAAATGAATTATTAACATTATTAGTTATTGTCAGATTACCTTTTCCTACGATTGATCCAATTACAAAATATAAGATTACTAAATTAAATGATAGTAATGAAGCAGTTATAGTTCC
TGAAATGATAATTAAACTGAAACAAGGTGTAGGAAGATTAGTAAGAACAAAGCAGGATAAAGGTTTATTAGTGTTATTAGATTCAAGAATGAATAAACCCATTTATAAGCATAAAGAAGCAGTATTAGA
TGCATTACCAATTAAAAATATGATTCATAAAGATGAAGTCAAAGATTTCCTTAATAACATTTAAAGTAAGTAGTAATAGTTATCCAATATTTTCTAGTTGATTCTCTTATAATATGTCCAATTAAGTGTAGA
CGATTCAAGATGATATTGAGGAAAGGATGAGAATGTGTCTTTCAGGTAGCTCATTTGATGATAACCTTAAACATAATATTTACTTTCATCCTGCTGAAAAAAGAGGTTACAGTTTCAAGCCGATAGAATAC
ATTGGGCTATATAAAGACAAAGCAATAAAAGCTATCGGAAAAGTAGACAAAGTAATAGTGACGGAAATAAATGAGAGCTCATTATCCTTGAAGACAGTTTATCCTGTAGGTACTGAGTTATCGATTGATG
AGTATGAAATAGTTAAAAATAAAATAATGGTGCTTGGGAAAGAAAAGTGGACTAATCTATTGAACGAACCCCATTATTATTACTTAATTGAAGATTTTATAGAGACAGACTACAAGAAAACATCTAAAGG
TGGATTGATGGGAGTTAAGTATTTTAATGTGAATGAAATTTTGAATAGGGATTGCTTAACCACCGAACAAATCGCTAAAGAATTATGTAATAAAGATTGGGAATGATAAATAGTGAATGAAGCTATAATG
CAATAATGTATGTATAACATAATCAAGTAATGAATTTAATGATAAGAGAGTATGGAGTAAAGAATTGAATTAGTTTGCATGGTCTATGTATCTTAAAGGTGTTGAAGGAGATACACCGAAGTATGCAGTA
CCAATTCTTGAAACAGACTTTAGTGATTTACCATCTACACATACGACAGTCGGATCACTTGATCGGTTTCGAGATAAGACAATGAGCATTAACAGAAGCGGGAGTAACTGTTGAATTCCATTTATATCCAG
TTAGATACTAGAGTTTGAAGTGATAAATCTTGATTCGGATGCTGAAATAAGTAAAAGAGCAGTTACTGAATATAATATAAGTATAAATATGCCACTAGATATTTTAAAGGGTATTTATTCACTTTTATAAA
ATCATCTGCTAAGATAGTATGGAAGATTTTTATAGGAGCTGATTATAATGCGACATATAAATATGTCATACATCATACGCTATAGTACTCTAACTCAAAGATAAGAAGTACTTAGTTATGAATAGCATTCA
TTTCACAAAGTACTTCAACAAAAATGGAGGAATATGAAATGAATAAAATAGAAGTGTATAAGTTTGTTAAAGTAAAGCAGTTAGTATATCAAATGATTAAGTTATATCGTACAAACGATATGAATTCCCA
TAAAACACAAAAAGATTTTTTACTAAATGAAATTAATGATATCTTTAAAGAAAAAGATATTGATATCTCGGACTTTATTACATCGATTGACGATGTAAAATTAACTAAGAAAAAAGCAGAACATCTTTTAA
ATGAATTAAAAGTGTACATCCAAGATTTTGAAATACCTTCATCAAGTCAACTGGAGAAAATTTTTCGTAAAGTAAAAAAATTAAAGAGACCAGATATAAATTTAATTGATACAAAAGAAATTTCATATTT
AGGATGGAATGATAATTCTTCTAACCGAAAATATATCGTTTATAAAAATTTAGATGATAAATTCGAAGGTATATATGGCGAAATTTCACCAAATAAAGTAAAAGGATTCTGTAAAATTTGTAATCAGGAA
TCTGATACATCACTCTTTCTCAATAAAACTAAACATAATAAGAGTAGTGGAACATATACTAAAAAAGGAGATTACATTTGTTATGACAGTTTTAAATGTAATCAGAACCTAGATGATATAAATAATCTTTA
CGAATTTATTGTTAAAATAAAATAGATCCAAACAGCCCTGATCTTTAGAACTAATGATCAGGGTTGTTATATTTTGTACCTCACTCTTAATCGATACGCTGAAAGTCCCTATAAATAGAACGTACGTTCCGT
TTGTAGTATAATGACAAAAATATTAAATTAGAGGATTAAAAATGGGATTATAATTTTTTAATGTACCAAACTGAAAAAGGCTAAATAAAGATAAATATAAGATTAGAAAAAACTGTATGGATTAGGCAAT
ATTTATATATAATGAAATGAATTAACAGAAAATTGAATTATCCGGAAAAACCGTACTGTTCGATTGGAAGGGAATAGAGAAGGTGAAAAGAATTAACTTCAATTATCATCTTATTTCTGAAAAAAGATAT
GAAAAACGGACGCAAGACACTTTTGGACTGACCCCAATGAGTGGGAATGATATAAAAACACTTTCGTTGAATTCATATTAAAATGAATCATGCGAAGGTGTTTTTTCTATGAAAAGAGTTTCTTATTCAGT
AGAAACAAAGTATAAAACTGTTGAAATGAAAGCAGCAGGATTTTCAACAAAAGAAATTATGGAAGAATTAAATATTAGAAATAGAACACAAGTGGAAACTTGGTGGCGATGGTATCGAAATGGGGAAA
GTTATAGATTTTCACAACACGTTGGTAAACAATATACCTACGGTAAAGGATTAGAAGAGCTGTCAAAAGTAGAACAATTAAAATTAGAAAATAAGAGAAAAGATATAGAATTGGATATTTTAAAAAAGT

ACAAGGCATTGGAAAGGAAGTGGTACCAACAGTAGTCATAGATTTAGTAGATCAATTAAAAGGAAACTATTCAATCAAATTGATACTAGAAGTATTAAACATACCTAAATCAACATATTACCGATGGAA

AAACAAAACCTATAAAAATGATACTGTAACACAAAAAGTTATTGAATTATGTGAAGCTAACCACTATACCTACGGTTATCGTAAGATTACAGCACTGATTAATCAATGTTATACATCATCAATTAATCATA

AGAGCGTACAGAGAATGATGCAGAAGCATCATTTGAACCGCCGAGTTAGACCTAAAAAGACGACAAGGATAGGTAAACCGTATTATAAAACAGACAATTTATTACAAAGACAATTTAAAGCGAGTTGTC

CCATGGAAGTATTAACAACCGATATTACTTATTTACCATTTGGTCATTCTATGTTGTATTTATCTTCGATAATGGATATTTATAACGGAGAAATTGTGGCGTATAAAATAGATAATAAACAAGACCAACGTT

TAGTTAATGATACATTAAATCAAATCGATATACCTGAGGGGTGTATATTACATAGTGATCAAGGCAGCGTTTATACATCTTATGCTTATTATCAATTATGCGAAGAAAAAAGCATTATCAGAAGTATGTCC

CGAAAGGGAACACCTGCCGATAACGCCCCGATAGAAAGTTTCCATTCCTTGCTAAAGTCTGAAACTTTTTACATCAATAATCAGCTTAATAGCTCTAATCATATTGTAATAGATATTGTCGAAAAATACAT

TAAAAACTATAATAATAATCGAATTCAACAAAAGCTAGGCTACTTATCCCCTGTAAAATACAGAGAATTAGCAGCCTAGAACATAGTGTTTTTATTAAGTTCCCATTTTAAGGGTTCAGTGCCCATAATGT

CATGGCTTTTTATATTGAAAAAACATGCGATAATTTGTTCCCAAATGTCCTTATAGTTGTACTAATATCTGTAATAGAACACTAGAAACTTGGTTTAACCTTTCCTTTTCGTAAATAAATGTAAAGATATAC

CATAAGGATCTCTTACATAACCATAACCTTCAGTATAGAACTCTGGGCTGAATGCTTTTAATACTTCACTGCCTTTTTCTATTAACTGGTCATACACACGCTTAGTTTCTTCTACATGATCAAAAGTAATACA

AAGCGATATATTATTACCTTGGGTTATAGGTAAACCTTCGGTATCATCCGCAATCATAATTTTTATATCTCCAAATTGAAGTACACATTGATCAATTTTATTTAAATCTTTTTTATCGATATTAAGTTGCTTA

TCTGTCGGTCTATCCTTGATACGTTGGATATACAGTGTTTTAGCGCCAAACAGCTCTTCATACAACCTTTTCAAACCATCTGCATTTTCTATAATTAAAAAAATAGGGACTTACTTGAAAATTCATAGTTTTTC

CTCCTTAAATTTGTTATAAATAAGTATATACTGTATTAGTGTCACCTATTGATACTAAAAGGGGAATAATAATGAAAAAATCTGTTAGATTATATCATATGATTGAATACTGTAATGAAAAAAGGAATTTC

AAGTTAAATGATTTAATGTCAGAATTTAATATTTCTCGTAGTACTGCTTTAAGGGATATAAAGGAAATTGAAGCATTAGGAGTGCCTTTATATAGTAGTACAGGGAAAAATGGCGGTTATATGACCATAGG

TAATCGAAACCAAACGAAAATAGCAATCACAGACGAAGAATTGAAAGCTTTAGTATTTACACTTTCAAGTATCTCCAATGTTAGTAAGTTACCTTTTCAAACAGAATATCAAGAAATATTAAAAAAATTA

TATAATAATTCGAATAAAAAAGAATTGATAAACCAGTATAATGAGATATTTCAATATTTTAATGAAGATACATATCAGTTCAAAAGTTATAAGATATTTAATGAAATCATTAGATTAACAATTGGTAGTAA

GTCATTTAAAATCTGTTATTCACAAAACTACTTGAAAGAACAATATAAAGGTATTGGTATCTTATATAAAAATCAACAATGGTACTTCGTTGTTGTTAATATAGCATCAAAATTAGTAAAGTTGTTAAACA

TTTCTAAAATAAAAGAAGTATATGAAATGGGAGAAACTAAAGAATGTAATGAAATAACTATGCAGAATTTTCAACAGTTTATGGTTGAAAACGAAAATACGATTGATATACAAATTAGAAGTAATATTAT

TGGATTAAATATCTTGAAAGGATACTTATGGAGTGACTATATGATTGAAAATATTAACGAAGAGACATATTTGTTTAAATCAAAAGTGAACATGCAAGATATAAATTTTATAGCTAAATTAATAGTCACAT

GTGGTGCCAATGTCAAAGTAGAATTTCCTATTAGTTTGAAAAAAGCTATTAAAACTGAACTAACCAAAATAATAAACTTATACTAATGGTAATTTAATTATGATTGCAATAACAAATAAAAGATAATCTTA

AACATATTAAATTGATCCGTAGTATAAAAACCGCATCATTAACCGATACGCAGAGGCGTATCATAAGTAAAACTAAAAAATTCTGTATGAGGAGATAATAATTTGGAGGGTGTTAAATGGTGGACATTAA

ATCCACGTTCATTCAATATATAAGATATATCACGATAATTGCGCATATAACTTAAGTAGTAGCTAACAGTTGAAATTAGGCCCTATCAAATTGGTTTATATCTAAAATGATTAATATAGAATGCTTCTTTTT

GTCCTTATTAAATTATAAAAGTAACTTTGCAATAGAAACAGTTATTTCATAATCAACAGTCATTGACGTAGCTAAGTAATGATAAATAATCATAAATAAAATTACAGATATTGACAAAAAATAGTAAATAT

ACCAATGAAGTTTCAAAAGAACAATTCCAAGAAATTGAGAATGTAAATAATAAGGTCAAAGAATTTTATTAAGATTTGAAAGAGTATCAATCAAGAAAGATGTAGTTTTTTAATAAACTATTTGGAAAAT

AATTATCATAATTTAAAAACTGACAATTTGCGAGACTCATAAAATGTAATAATGGAAATAGATGTAAAATATAATTAAGGGGTGTAATATGAAGATTAATATTTATAAATCTATTTATAATTTTCAGGAAA

CAAATACAAATTTTTTAGAGAATCTAGAATCTTTAAATGATGACAATTATGAACTGCTTAATGATAAAGAACTTGTTAGTGATTCAAATGAATTAAAATTAATTAGTAAAGTTTATATACGTAAAAAAGAC

AAAAAACTATTAGATTGGCAATTATTAATAAAGAATGTATACCTAGATACTGAAGAAGATGACAATTTATTTTCAGAATCCGGTCATCATTTTGATGCAATATTATTTCTCAAAGAAGATACAACATTACA

AAATAATGTATATATTATACCTTTTGGACAAGCATATCATGATATAAATAATTTGATTGATTATGACTTCGGAATTGATTTTGCAGAAAGAGCAATCAAAAATGAAGACATAGTTAATAAAAATGTTAATT

TTTTTCAACAAAACAGGCTTAAAGAGATTGTTAATTATAGAAGGAATAGTGTAGATTACGTTAGACCTTCAGAATCTTATATATCAGTCCAAGGACATCCACAGAATCCTCAAATTTTTGGAAAAACAATG

ACTTGTGGTACAAGTATTTCATTGCGTGTACCGAATAGAAAGCAGCAATTCATAGATAAAATTAGTGTGATAATCAAAGAAATAAACGCTATTATTAATCTTCCTCAAAAAATTAGTGAATTTCCTAGAAT

AGTAACTTTAAAAGACTTGAATAAAATAGAAGTATTAGATACTTTATTGCTAAAAAAACTATCGAATTCTTCAACTACAGAAAATATATCTATAGATATATCAAGATTTTTAGAACTAAGTAATATGATAC

TCTTGTTAGATGATATGCTCGACGTCAATATATATATATAAATTCTTTTAAAAATAATACATTAGAGACATTTGATGCGACTGATCCTGAAGTTGATTACATCACTGAAATAGGAGATTACTTATTAAAATA

TGACGTTAATTCTATAAATGATGTCAGAATTGAAGTGATAGATAATTTGGGGCATTCAAATAATATGCTACTAAAAACGATACTACATGCAGAAGTTGAAATGGAAGATGGAAAGAAATATTTATTACAA

AATGGGAAATGGGGTTATTTTAATAGAGAATTTTTTGACCTTTTGAATGATCATTTGAATGAGATAGAAATTAGGTATAACACACTAACTCCCACAGGTTTAGTATTTAAAGAAGGAGAAGAAGGATATA

TAAAAGAAATAGTAGGAAGATTGCCAGAAGAATATTTAATGTTACATAAAAAATTCATAAAACCAATAAATAAGAATTTTATAGTAAAAGGAAATGGAATAGAGTTGGCAGACTTATACAATATTAAAA

ACAAAGAGCTTTTCACGATTAAAAGAGGAATTAATACATCTTTATCTCTTTATAGTCTAGAACAGAATATAATAGCAATTAACGCCTTAAAATATCCAGAATCATATAATTTTGAAGAATTAATAGAAGCT

ATTCCTGATAACTCGGAAAATATATTTAATGATATACAACGGAGTACAAATTTTAGTATAGTATGGATTTTACCGATATCATCTATTGATAATATGCCCATAAAAGATATGGTTCATACTAGTAACGTAAT

AAATAAAAACTTTCAATTAACTAATTTAGGTTCAGTATTACTTAAAAATAAATTAGTAGAGTGGTCTCTCTATTTAAAAGATCAAAGAATTAATCCAATTATTTATATGGAAACGCCAACTGAAGATAGAA

ATTAAACTTTTATTTTAAATCACCCTAGTTCATACCTAAAAGACTTTCACACAAACAAAGGAGGAAACTTAAATTCCTCCTTTCCCTTATTACTCATACTATAATTCAATTTTAACGTCTTCGTCCATTTGGG

CTTCAAATTCATCTAGTAGTGCTCGTACTTCTGCAATTGATTGTGTGTTCATCAATTGATGGCGAAGTTCGCTAGCGCCTCTTATGCCACGCACATAGATTTTAAAGAATCTACGCAAACTCTTGAATTGTC

GTATTTCATCTTTCTCATATTTGTTAAACAATGATAGATGCAATCTCAACAAATCTAATAGTTCTTTGCTTGTGTGTTCGCGTGGTTCTTTTTCAAAAGTGAATGGATTGTGGAAAATGCCTCTACCAATCAT

GATGCCATCAATACCATATTTTTCTGCAAGTTCAAGTCCTGTTTTTCTATCGGGAATATCATCGTTAATTGTTAACAATGTGTTTGGTGCAATTTCGTCACGTAAATTTTTAATAGCTTCGATTAATTCCCAA

TGTGCATCTACTTTACTCATGCGTTTGATAAAAACTTAAATAATATTAATTCGGTCATCAGTGGCGTTAAATCTTTTATCATTTTTAGTTATAGTTGATAAATTTATATTTATAAGCATATATGGATATTTCA

TCAAAAATTTTTATTTATATAAATCCGAACTGCATACATATTTGTTTAAATAAGAGGTATTATTTTTCGGGAAATTGCTGTCTGAGTTAAAAGGATTAGTTTTATAAAATGAGTTGAACTATAGCAAAAACG

ATTAAAATACTGATAATCCATTTTTGTATTATGTTAGGGACTTTTTTACTTAATTTTAACCCTATTGGAGCAAATATAATACTCCCTATTATAAGGAATAAGGCGTCATATAAAGGGATATAACCTTGAATA

AGTTTGATGACAAAAGCACCAATTGAAGATATAAAAGCAATTACTATACTATTAGCGACTACAGTATTCATTGGTAATTTGAATAAAACCAATAATATAGGAATAATAATGAAGGCACCACCTGCACCTA

CTATACCTGAAATAATACCAATGAAAAGGCCAATGATAACTAATAAATATTTATTAAATGAAGACTTTTCGGAACTAGGTTTCACTTTAATAAACATTAATGTTAATGCAAGTAAAGCAATAATGATATAT

ACCGTATTTACAAATGTAGCATCAAATAAATTTGCTAGAAATGCACCTAACATACTCCCTATAATCATGCCGCCACCCATATAAAGAACTAATTGTGGCGAGAACTCTGTTTTTTTTCGAGCTTTTAATGAG

CCACTTAATGTACTGAAAAAGACTTGGCTAGAAGTAAGACCTGATGCGATATATGCGCTATATGCAGGGGCTCCGAATAATGGTGGTAATAATAAAATAGCTGGATAAATAATGATAGCACCACCTACGC

CTACTAGACCAGATATGAACCCACCGAATACCCCAATGAGTAACATGATAACTATATTAACAATATCCATTACTTACTTTTCACCAATAAGTTTACAGCTTCATTAATTAACTCTTGGGAGCTTTCTTCATC

ATCCGCAGCTGCTTTTACACATTCTATTAAATTCTCACTAATAATGATACCCATCAAGCGTTGGAGTGAACTCTTTGATGCACTTATTTGTGTAATGACATCTTTACAGTCTTTTCCTTCCTCCATCATTTTAA

TAATTCCATTTAGTTGCCCTTGTATTCTATTAATACGATTAATCATTTTTTTATCATAATTCATAGTCATACCTCCACTTTTAATTGAATAAAAATATATTAATAGATAAACACAAATGTGTCAAATACCCCT

AGAGGTATTTGACAAGTTCCATCCAACTGTTTAAAATACCCCTACAGGTATTTTTAGGGAGGTTATTATGAAACAATACGGAGAAAAGTTTATCGATGAATTTAGTAAAGCAGAATTGGAAAAACTAGCC

AAGCAAGGGCAATTAATTGACGTTAGAACAGAAGAGGAGTATGCATTAGGACATATCAATGGTTCCATACTTCATCCTGTTGATGAGATTGAGTCATTCAATAAAGAAAAAAATAAAACCTATTATGTAA

TCTGTAGAAGTGGTAACAGAAGTGCTAATGCTAGTAAATATTTAGCTAAACAAGGTTATAACGTTATAAATCTTGATGGTGGTTATAAAGCTTATGAAGAAGAAAACGATAGTTATGATACACAAGAAGA

ATATAAAAGTATAGAAATTAAAGCAGATCGTAAACAATTTAACTATCGTGGTCTTCAATGTCCAGGGCCAATTGTAAAAATTAGTCAAGAAATGAAGAATATTGAAGTAGGTGACCAAATTGAAGTCAAA

GTCACAGACCCTGGATTCCCTAGTGACATTAAAAGTTGGGTGAAACAAACAAGGCATACTTTAGTTAAGCTTGATGAAAATAACAATGGAATTAATGCGATTATTCAAAAAGAAAAAGCAAAAGATTTA

GATATAAATTATTCTGCTAAAGGTACTACAATTGTATTATTTAGTGGAGAATTAGACAAAGCTGTAGCAGCGTTGATTATTGCAAATGGTGCTAGAGCTGCTGGAAAAGATGTAACTATCTTCTTTACTTTT

TGGGGGCTTAATGCATTAAAAAAAGTGCAAACAGTTAATGTTAAAAAGCAAGGTATTGCAAAAATGTTTGATTTAATGTTGCCCAAAAAGAATATACGAATGCCTCTTTCCAAAATGAATATGTTTGGTTT

AGGAAATATGATGATGCGCTACGTAATGAAAAAGAAAAATGTTGATTCATTACCAACACTTATCAATCAAGCTATTGAGCAAAATATCAAATTAATCGCTTGTACGATGAGTATGGATGTCATGGGTATT

CAGAAAGAAGAACTTAGAGATGAAGTTGAGTACGGTGGTGTAGGCACTTATATTGGTGCTACTGAAAATGCGAATCATAATTTATTTATCTAATTAAATCTATTAATAAAAGGAGTTGTTATCATGTTTTT

TAAACAGTTTTACGATAATCATTTATCTCAAGCATCATATTTAGTGGGTTGTCAACGTACAGGAGAGGCAATAATAATAGACCCTGTTCGTGATTTATCGAAATATATAGAAGTTGCAGATTCTGAAGGTT

TAACAATTACACAAGCTACAGAAACACATATTCATGCTGATTTTGCTTCAGGAATTCGTGATGTGGCTAAACGCTTAAATGCAAATATATATGTGTCTGGCGAAGGTGAAGATGCATTAGGGTATAAAAA

TATGCCATCAAAAACACAATTTGTTAAACATGGAGATATCATTCAAGTAGGCAATGTTAAATTAGAAGTTCTGCATACTCCAGGACACACGCCTGAAAGTATTAGCTTTTTACTCACTGATTTAGGTGGTG

GTTCAAGTGTTCCGATGGGATTATTTAGTGGTGACTTTATTTTTGTTGGTGATATAGGTAGACCTGATTTATTAGAAAAAATCTGTTCAAATAAAGGGTTCTACAGAAATTAGCGCGAAACAAATGTATGAG

TCCGTTCAAAATATTAAAAATTTACCAGACTATGTTCAAATCTGGCCGGGTCATGGTGCTGGAAGCCCTTGTGGTAAAGCATTAGGTGCCATACCTATATCTACAATAGGTTATGAGAAAATTAATAACTG

GGCATTTAATGAAATTGATGAGACTAAATTTATTGAATCATTAACATCAAATCAACCAGCACCACCGCATCATTTTGCACAAATGAAACAAGTTAATCAGTTTGGTATGAATTTATATCAATCATATGATG

TTTATCCTAGTTTAGATAATAAGAGAGTAGCATTTGATCTTCGTAGCAAAGAGGCCTTTCACGGTGGCCACACAAAAGGAACAATCAATATACCATACAACAAAAACTTTATTAATCAAATTGGTTGGTAC

TTAGATTTTGAAAAAGATATAGATTTAATTGGAGATAAATCTACTGTTGAGAAAGCGAAACACACTTTACAATTAATTGGGTTTGATAAGGTAGCAGGCTATCGTTTGCCAAAATCAGGCATTTCAACCCA

GTCCGTTCATAGCGCTGATATGACAGGTAAAGAAGAACATGTATTAGACGTACGTAATGATGAAGAGTGGAATAATGGACACTTAGATCAAGCAGTTAATATTCCGCATGGTAAATTATTAAATGAAAAT

ATTCCTTTTAATAAAGAGGATAAAATATATGTACATTGTCAGTCAGGTGTTAGAAGTTCAATTGCAGTGGGTATATTGGAAAGCAAAGGTTTTGAAAATGTGGTGAATATTAGAGAAGGCTATCAAGATTT

TCCAGAATCATTAAAATAATTTAAGGATGTGGAAAAAAATGAATAAGCATTATCAAATTGTTATTATTGGTGGCGGTACAGCAGGTGTTACCGTAGCATCAAGACTATTAAGAAAAAAATCAAAACTTAAAA

GAGAAAATAGCAATTATAGATCCAGCAGACCATCATTACTATCAACCATTATGGACGTTGGTTGGTGCAGGGGTATCTAGTTTGAAAAGTTCTCGTAAAGATATGGAAAGTGTTATACCTGAAGGTGCTA

ACTGGATAAAACAGGCTGTTTCAAGTTTTCAACCTGAAAATAATAGCGTTATTTTAGGAGATAATACAGTCGTTTATTATGATTTTTTAGTAGTAGCTCCAGGATTACAGATTAATTGGTCTTCAATTAAAG

GACTAAAAGAAAATATAGGTAAAAATGGTGTTTGCTCTAACTATTCACCTGACTATGTTAACGAAACTTGGAACCAAATTTCTAATTTTAAACAAGGAAATGCCATTTTTACGCATCCAAACACTCCTATA

AAGTGTGGAGGTGCGCCTATGAAAATTATGTATTTAGCTGAAGATTATTTTAGGAAACATAAAATCCGTTCTAACGCTAATGTGATATATGCAACGCCAAAAGATGCTTTATTTGACGTAGGAAAATATAA

TAAAGAATTAGAAAGGATTGTTGAAGAAAGAAATATAACAGTCAATTATAATTATAACCTTGTTGAAATCGACGGTGACAAAAAAGTGGCTACATTCGAACATATCAAAGCATACGATAGAAAAACAAT

AAGTTATGATATGTTACATGTAACACCACCTATGGGTCCCTTAGATGTAGTAAAAGAAAGTACACTTTCAGATAGTGAGGGTTGGGTAGATGTTAACCCAACCACATTACAGCATAAAAGCTACTCTAAT

GTATTTGCACTTGGTGATGCTTCAAATGTACCTACTTCAAAAACAGGCGCAGCTATTCGTAAGCAAGCACCTATCGTCGCTAATAATTTATTGCAAGTGATGAATAATCAAATGTTAACGCATCATTATGA

TGGTTATACTTCATGCCCTATTGTTACTGGATATAATAGGTTAATACTTGCAGAGTTTGATTATAATAAAAATACTAAAGAAACAATGCCGTTTAATCAGGCCAAAGAACGTAGAAGTATGTATATATTTA

AGAAAGATTTATTACCTAAAATGTATTGGTACGGCATGCTAAAAGGATTAATATAATAAAGTACAGAAAACAATAAATTTTTAATGAAAAATCTTTTACTATAAAAGATTAAGTATTTAAATGACGTGTC

AGTGTTGTGTTTATATGTCGTGAATTTTTAGCTCTAAATAGTATAAGATTGAAAAAGTTGTTACTGTTTTAAATGATCACGATGAAGTCATTCAATAAGAATGATTATGAAAATAGAAACAGCAGTAAGAT

ATTTTCTAATTGAAAATCATCTCACTGCTGTTTTTTAAAGGTTTATACCTCATCCTCTAAATTATTTAAAAATAATTAATGGTATTTGAGCACGTTTAGCGACTTTATGACTGACATTACCAATTTCCATTTCT

TGCCAGATATTCAAACCACGTGTACTCAAAATGATAGCTTGGTATGTACCTCCAATAGTAATTTCAATAACTTTGTCTGTTGAACACTAAGAGCAATTTTAATTTCATAATGTGTTGTAAACATTTTTTTTG

ATTGGAGTTTTTTTCTGAGTTAAACGATATCCTGATGTATTTTTAATTTTGCACCATTTCCAAAAGGATAAGTGACATAAGTAAAAAGGCATCATCGGGAGTTATCCTATCAGGAAAACCAAGATAATACC

TAAGTAGAAAGTGTTCAATCCGTGTTAAATTGGGAAATATCATCCATAAACTTTATTACTCATACTATAATTCAATTTTAACGTCTTCGTCCATTTGGGCTTCAAATTCATCGAGTAGTGCTCGTGCTTCTGC

AATTGATTGTGTGTTCATCAATTGATGTCGAAGTTCGCTAGCGCCTCTTATGCCACGCACATAGATTTTAAAGAATCTACGCAAGCTCTTGAATTGTCGTATTTCATCTTTTTCATATTTGTTAAACAATGAT

AAATGCAATCTCAATAGATCTAATAGTTCCTTGCTTGTGTGTTCGCGTGGTTCTTTTTCAAAAGCGAATGGATTGTGGAAAATGCCTCTACCAATCATGACGCCATCAATGCCATATTTTTCTGCCAGTTCA

AGTCCTGTTTTTCTATCGGGAATATCACCGTTAATTGTTAACAATGTATTTGGTGCAATTTCGTCACGTAAATTTTTAATAGCTTCGATTAATTCCCAATGTGCATCTACTTTACTCATTTCTTTACGTGTACG

AAGATGAATAGATAAATTGGCAATGTCTTGTTCGAAGACGTGCTTCAACCAATCTTTCCATTCATCGATTTCATAGTAGCCAAGGCGTGTTTTAACACTTACCGGAAGCCCACCTGCTTTAGTCGCTTGAAT

AATTTCGGCAGCAACGTCAGGTCTTAAGATTAAGCCGGAACCCTTACCCTTTTTAGCAACATTTGCTACAGGACATCCCATATTTAAGTCTATGCCTTTAAAGCCCATTTTAGCTAATTGAATACTCGTTTC

ACGGAACTGTTCTGGCTTATCTCCCCATATATGAGCGACCATCGGCTGTTCATCTTCACTAAAAGTTAAGCGTCCGCGCACACTATGTATGCCTTCAGGGTGGCAAAAGCTTTCAGTATTTGTAAATTCAGT

GAAAAACACATCCGGTCTAGCTGCTTCACTTACAACGTGTCGAAAGACGATATCTGTAACGTCTTCCATTGGCGCCAAAATAAAAAATGGACGTGGTAATTCACTCCAAAAATTTTCTTTCAT

> SCC*mec* from Strain 20121643 (ERR1595888/SAMEA3924203-0001 len=1012623_cov=33.8_gc=0.331

ATGAAAATCACCATTTTAGCTGTAGGGAAACTAAAAGAGAAATATTGGAAGCAAGCCATAGCAGAATATGAAAAACGTTTAGGCCCATACACCAAGATAGACATCATAGAAGTTCCAGACGAAAAAGCA

CCAGAAAATATGAGTGACAAAGAAATTGAGCAAGTAAAAGAAAAAGAAGGCCAACGAATACTAGCCAAAATCAAACCACAATCCACAGTCATTACATTAGAAATACAAGGAAAGATGCTATCTTCCGA

AGGATTGGCCCAAGAATTGAACCAACGCATGACCCAAGGGCAAAGCGACTTTGTTTTCGTCATTGGCGGATCAAACGGCCTGCACAAGGACGTCTTACAACGCAGTAACTACGCACTATCATTCAGCAAA

ATGACATTCCCACATCAAATGATGCGGGTTGTGTTAATTGAACAAGTGTACAGAGCATTTAAGATTATGCGAGGAGAAGCATATCATAAATGATGCGGTTTTTATTAATTAGTTGCTAAAAAATGAAGTAT

GCAATATTAATTATTATTAAATTTTGATATATTTAAAGAAAGATTAAGTTTAGGGTGGATGAATGAATGGCTTATCAGAGTGAATATGCATTAGAAAATGAGGTACTTCAACAACTTGAGGATTTGAACTA

TGAAAGAATAAATATACATAATATTGAATCAGAAATTAATGAATATCTCAAAGAACTAGGAGCGTTAAAACATGTCTGAACAAATAAATACTCCAAAACTGAGATTTCCAGAGTTTAGAGATGAATGGG

AAATGAAAATTATTAAAGAATTGTTTAATGTTGTTTCTGGCTCTACACCTCTTAGATCTAATACTTCTTATTATGAAAATGGAAATATACCTTGGGTAAAAACTACAGACTTAAATAATTCGTTAATTAATG

ATACTTCTGAAAAAGTAACTGATATTGCTTTAAATAACTTGAAGGTTTTACCTAAAGATACTGTACTAATAGCTATGTATGGTGGATTTAATCAAATTGGTAGAACAGGAATTTTGAATATCAAGGCAACC

ACTAACCAAGCAATTTCGGCTCTAATAAAAAAGGGTAATTATAATTCTAAGTTTTTGCAATCTTATCTCAACTTTAATGTAAAACAGTGGAGACGTTTTGCAGCAAGTAGTCGAAAAGATCCTAATATTAC

TAAAAGAGATATAGAAAAATTTAAAATACCATATACATGTCTAGAAGAACAAGAAAAGATTGGGGGTTTCTTCAGTAAAATCGACCGTCAGATTGAGTTAGAGGAAAAGAAATTAGATTTGTTAGAGCA

ACAAAAGCGTGGATATATGCAAAAAATTTTTTCACAAGAATTGCGATTTAAAGATGAGAATGGGAATAAATATCCTAATTGGCAAACAGTTAAAATTGGAAGCATTTTAAAAGAAAGAAAGGAACGTTC

AGGAGATGGAGAAATGCTTTCTGTGACAATTAATCATGGTATTGTAAAATTTGATGAGATAGATCGAAAAGATAACTCTAGTAAAGACAAAAGTAATTATAAAAAAGTTTATAAAAATGATATAGCTTAT

AATTCGATGCGCATGTGGCAGGGAGCTAGTGGAAAAGCTGAGTTTGATGGTATTGTAAGTCCAGCTTATACAGTTGTTACTCCAATTGAAAATATTAATTCAAATTTTATAGCATATTATTTTAAAACTCAT

AATATGATTCACAAATTTAGAATTAATTCACAGGGACTTACTTCAGACACTTGGAATTTAAAGTATAAACAACTAAAAGACATTAAAATTAGTATCTGTTCAAAAGAAGAACAAGATAAAATTGCTGATT

TATTAACGATATTAGACACACGTATTAAAAAGCAAAATCACAAATTAGAAATATTAAACATTAATAAAAAAGGCTTACTACAAAAAATGTTCGTTTGATACTGATAACGACCTATTATGTCAATTAAAAG

GAGAGTTGTATTTATGAACGATGAAAATTTAAACGATGAAAATTTAAACATTGTTATGTCAGTGCTAGTTGACATTTCCTTAAAAGCTAAGCAATTTAGACTTTTAAACCAAGAAAAAGAAAAGATAGAA

AAAATATTGGAATTATTGGTTTGTGAAGAACAAATTGTATTTTTAGGAAACACTGCTGCAAGTAACAAACCTTATTTGTTTCTTATCCCTAAATATTATGCATTAGAAGAAGAAATCATAGATATAGAGTGAA

TACAAATTTAGATTTGTATACCATTGAAAATAATCAGTATCACAAAGTTCGAATGAATGATTCAGGTATATTAGTGAAAATTCGTATTGACCAGGATAGTTTTGATATAACTGAAAAAAGTAGATATGTCT

CAAATGAACTGGTAGTATAACAAATCTATTAACTTTCATCTATAATTAAAAAATATAAGAAATTAGAAAAAATAAATTTAAGAGCATCCCTCACCGTAAAAGTGAAGGATGCTTTATTTTTATTGTAAGTA

GTATGATATTCATTTTGATAGATATTGGTTAATAATTGCCTGTAAATCATTATTAAGTACCGTTGTTATAGAGTCATCATCCATAAAATCATCATAGATTTTATCCAATATTTCCTCATCTTTAATTGATGTA

AAATGATGTACTAGTCCTTTTACGATAGATATATAATTTTGAGATGCTAAGCAACTAGCAACTGTTCGCCAGTCTGATTGTGTAGTGTCATGGTTCATAGATAATCCTCCTTTTATTTCAATGTCCATTTTTG

ACGCGCTTTAGGATTGAGTGGATGCATGATTTCATTTGTTGCTGGGTTTATAAGCTTTTTAATTTTCTTTTTGTGAGGCTTCAACATTTCCATCACTTGCTCAACACGTTCGACGACAACAGGTCGTTTCGCA

TAAGCACCATAAGCTAGAATCACTGTGTCACTTTCGCTTATCGCTTTCATCAAGTGGATGTCTGTGTGTTTATCATAAGGTTCTTTAATATGTTTAAGGTTCTCTGGGGTTTTAATATTAGAGAATAGATTTA

CAAGATATACAGCACCATACTGTTCAGAATTCGCTAATTGGTTAAGGATAAGAACAGTTGTAAGATCGAGTGATAATACGCCATCTAAATGAGGGTACATCGTTATCACTGTACAAGCGGGTTTCTTTTCA

TCCCATGTTTTCTTGAGTAAGTAGCGGTGTTGTTCATCATCGCTAAATATGGCTTCTGTGTGTATCGTACTTTTGATTGTATTCATCATCGTCACTCCTTTTAGTATTCTTCTGGTAAAAGCATCACATAATAA

AAAGCGTCCACGTCATCTTCACGAATGACGTAGACTTTCTTAGGTAATGCGTTTTGATTTTTTTCATAGTTTGTATAGTGATATTCCAATTTGTATGCGGGTTGTTCTTGTTCATGCGTAATAGAGAGTATAT

TATCATCTTCTTGTAGTTTAAAAATGTGTAGGTAATCTGTATGAGGTTGATTGTCTCGATCTTTTACCATATTCCAAAGTAGTATTTGAAGGTCTAGAGATAGGTGTTCACTAATGCCTCTTGTGATGTATCG

ATTGATTTTCATATTATTTTACCTCGTCTTGAATTTCTTTCATGATGATAATCGCTTGGCTAATAATCGTAACAGATATTTGTGCCACTTTGATCCAATTATTCACGGTCATTCCCTCCTTGTTTTAGTAAATG

ACGTTCATCGATAATCGTACTTTTAGTATCTGTCAGGTATAGAAAGTCCATATCAAAATGATCCAAATAACCAATACTGATGAGTTGGCTATTGGCGTACATAAGGAATGGATAGATACTTAGCTCATGTA

GCTCATCATTATAGTAGGTATAAGTTTCAAGTGTAAGATGTGCAAGTGGGGTATGGTCTACAAAGCGAGTAGGAAGTATATTTTCTGCTGCGTCTTCTAACGCTTCACATTCCCATGTTTCGTTGTTAGACA

GTTGGAATAGACGAGTTATATATTGTTTGAGTTCTTGAGTGGTTGTTTTCATATCATTGCCTCCTAGATAGTGTGATAGCGATGTATTTCATATACATCACCGAGATAATATATATTTGATTTATCATTTATT

ACGAATCCCGGTGGGAATAAGAGAAAATTCCATATAAAAACCCGCTACAAACGTTGGTATAACAAGGAAATCCCGGAATCCCACTCATTTTGACGAACAATCAACTCATTATTTATAGGTATTGATGATA

TGTGTAGGTCTCTCTCTGCTTCTATATATATATTATTTATTTATAAAAAATAACGGGATTTTGGGATTGTGCTTGCACAATCCTTCTGCTTCTTCGAATCTGCAAATCCCATTCATTTCCCGTTAAAAAATCA

TTGTGGGATGTTCTTTAGCAATTTCAATATAAACTTCGTGTAGTTATGAAAATAATTACGACAATGACTGTTTTATTAGATAAGTGTTATTGAAATTGATAAAGAGAATTCTAAAAATGGTTAGATAAAAT

AAGTGAAAGAATAGTAGTGTAGTTATTGTTTATTCAATAGTTATATATATAGTTTGTGGCAAAAATAAAGACGAAGTGCTAAGGAGCACTTCGTCAAGTGGACGGTTATTAAATAGTTGTTTGATAATTTC

ATTATTCAGTTTAAGCGTAACATAAAATTGCTTCTTATGATGTTCATGGTTTCGGATATCAATACGATCGATAACCGTCAGATAGAGTGATTTCAACTGAGATTTCTCTAATTTGTCTATATCTTTGAATATT

GCTTGTAGTACGTTGGCAATCATATCAGCATCATAATGTGGAGCTTCTGCTTGTTTATCTTGCTCTAGTTGATGGATTTGATTATTTATTTGATTCAATTCATCTTGATAGTTAAGTATTGTTGGTTTCAATAC

GCTATCTAAGTCAGGTGAGTCTTCAATGGTTTTCGTTAATGTATGCATTTTGGCTTTTAGTTCTTCACATTGTGACTGTTTATAGGCAATATCATGATTCAAAGAAGATTTATCTATTTGGCTTTGTTCATTTA

CTTTTTCAACCAATTGCTTCAATACCTTTTTACTTTTGATAATTTCCAATATCTGGTCCATAACATATTTTTCTAATACATCTGCTCTAACACTATTGGCTGAACATACTTTAGAACCTTTGTTTCTAAAATTG

CTACATGAATAATATCTGATTCTTTTCTTAGTGCCATCTTTTAATGTATTGGTCGTATTACTTGCCGCCATGGCTGCGCCACATTTTGGGCACTTTACAATCCCAGTAAGTAGATTGGTCCCTTTACCATGAA

CTTGTGGTTTCTTGCGACTCTCTTGGCGTTTAAACTGTACTTTATCCCATAAAGCTTTATCAATAATAGGCGCATGCTTACCATCTGCGATAATCGGTTCCTCATTGAGTCCTTTTCGTCTTTTATCGCTCCAG

TGTCTATACTTCGCAAACTGTATCTTTCCAATGTAAAAAGGGTTTGAGATGATATAAGTAATGGACGAAATACTAAACGGTTTCCCTTTCTTAGTCACATAACCTTTATGATTCAATGTGTTCGCAATCTTA

CGATAACCGTGACCTTTGGCGTATGACTCAAAAATATATTTAACAATATTTGCTTCATGTTGATTAATCATAAGCTCTTTTTTACTCTCAGGTACTTTATCATAGCCTAAAGGTAAATTACCTTGGTAATAAC

CTTCAATAGCACGTTGTCTTTGGCCATTAAACACGTTTTCTACAATAACGTTTCTTTCAAATTCTGCGAAGCTCGCTAAGATTTGGAGCATCAACTTACCTGTGGAGCTCGCAATTTCTATTTTTTCAGTTAG

ACTAAAAAATTCAACATTAATCTCATACAATTCTTCGACAATATTTAATAAATCTGAGGTATTTCTAGCTAAACGAGTAGTATTATATACCATAATACCATCTAATTTACCTCCTTTAGCATCTTTTAACAT

ACGTTGTAGTTCAGGACGTTGCGTTGTTTTACCTGATATACCACGATCGGTATATTCATCAACGACTTCATAACCTTGAAATTGACAATACTCTGTAAGTTGATTTAATTGACCATGGATACTGTAACCATC

CGTTTGCATTTCAGTTGATACACGTGCGTATAATCCAATACGTTTCTTTTTGAGTTGTGTCATATGACTTCATTCCTTTCAGGAGTTATTAAATGCGATTGTTCAACGATATTGAGTGGACTGTTTTTAAAGT

AGATTCCTTGTAATTGTTTAGTTTGAGTTATTGTAATGTCATCAATCAAAGGTGCTATGATTTCTAATGTCAATTTATCTTTGACTACATGACGGATTGTTTCAGCAATTTGATTGGATGAATAATTAGAGTA

ACGTTCTTCTCTTTCAAAGCCAGCAGATAACCGTTTAAATGTTTCTATATCAATTTGATTTTGTGCTAGTTTTTCGATGAGTTGTTCTTGTGTTAGATGTGATTGTCTGTGTTTCATTTGTTGGTGTTTTAGAG

CTTTTAGTATTGTCTCATTTAATTTGTCATGAAATGATTGATTGCCACAGTATACTTTACAAGTGTTTAGGACCTCTGTTTCAATCATTTGCGCATTGATGCCTTTAAATGGACACGTATGATAGGCTTCATT

CATATTTTTAGGACAGACATAGTAACGTAGAGAACGATGTTCTTTTTTTATCGTTAAGTTTGTTAATGTTGATTGACAGTAAGGACATTTGATTCGTCGCTTTAACTTATTTCTAGAATTGGATCGATTGTGT

TGTTTATGAATACGACGCTCTTGTGCTTCCTCAAACGTATCAACATTAATAATAGGTGGAACGATATCATTAAACGTTCCATATTTATTAATGACACGACCACAATAGTTAGGGTTCAAGAGAATATTTCTA

ACTTGATAGGGCTTACGAGAAATAAAATTCATATCTTTATCCAGCTTTTGAGAAATTTTCTTGTAACCAAGTCCTTGTAAATACCAACGATAAACGGCTTTGATTGTCGGTGCTTCTTCTGCATTTACAATA

AAGTGACCTTTGCTGTAGTGATAACCAAAAGGGGCGTGTGTTGTAATCAACTTACCTTGTTTGGCTTTTTCTCTTATCCCATTTCGTGTTTGTTCGCTGATGTTGTTCGATTCCATTTCTGCTAGACTCATGA

GTATGTTTAAGCGAAAGCAATCAAACTCTTTAGACAAATCAAAATATCCATCGTTAACACTGATGATTGTGACGTGATGCTTTTTACAAATTTCAAAGAATTGTATAGCATTTTTGAAATTACGATGGAGC

CGATTTAGCCGATAGCAACATAATACTTTACACTCTCCAGATGTAATCACTTCTACCATTTTTTGATAGCCTGAACGCTTTGTATGTCCACCTGTTTTTTTATCATCATAAAATGTTACATTGGACCATCCAT

GTTGCTTAGCAGTGTCTATAATGAGGGATTTCTGAGTCGCTAAGCTTTGTTGTTTGAGTGTACTTTGACGTACATAAGCAATGGCTTTTTCCATTTTATACACCTCCATAGAGATAATATATGTTTATGAATG

AATTAAAATGAAAGGTCCAACGTGTTAAAAGCACGTTGGACCGCAATCTCAGTTATCACGATTTAACTCTTCAACAACTAAGTTAGCTAATACTTCAATTAACTCATCCATTTCATTCACTCCTATACAATT

TCATCTTTAAGTTACTAAAAAGGTATTGAGAAGTGGTCATTAAAGATCAGCTAACTCATCATTTTGCTTTTTCCATAACTTAAAATTCTCTTTGTTATCCGTATTAATTTTAGGTTTAATAGGTTCGGCATCT

TTAGTTAGACCAAAAATAGACGCATGGTCTGAATCTAGCTTTAATTGGTAAAAGACAAGCGACTGTTTCTTGCCATTGTTATCTTTGACTGTTCGTTTTTTCGTAATTCGATCACGGTCAGATTCGATAAAT

CCCTTATCCTTTAGTGCATTCACGACATTATTAACATCTTGGAAGTGATGCTCTGCCAACATATTTTTAAATACAGACGCAATGATTTTGACTTCGATATAATCATCTTTTAAGGCAATTAGTCCATAGTTCT

CCATCATATTCTTTAACGCTTTGTCATCAGAAAACTTACCACGGTTTTGTGCTACAAATTGCGTAATGACTTCGATCGCTTTATCTGCCAGTGATCGTTCAGAGACCGTATGAGCATGATAATCAATAAAGT

AGTCTCTTATTTTAGCGATATCAATATCTGTAGCTAAAACACGACCTAATATTTTCGCAGATGTTGTAATAACTGCATAACGCTTAAACATACGTTTACCTGTATTGTTTGTTTCATCCTTCAATTTATCTTT

AAACCAATGATGTTCATCTTTAAACCATTGAATGGCTTCATCTTCACGTTTAATAAGATAATCAGCAACGAACGGTAAAACATGCCCGTAGTTTTGTGCAACTGCTTTTTTGATATTATCAGAGTTAGTTGC

ATCTGTAGTAAATTGTTCATTAATCTCAATGGTTCTTACACGTAACCCATCGTTTTGAGCCGAATCAGTAAAAATACTGTATTCTGACGTTGAAATGACAGACGTGCCCCAATTCTTAGGCGTTTTAACTTC

TCCATGAACGTTTGAACGTTGACGACCTTGACCTTCAGCGATCGAATATAATAACCCCGTTGTATCTTTAAAAGTTGCTGATGAGAGTTCATCAAATACTAGTGGTACACCATAGTTGTTGCTTAAATAACT

TTCAAGTGCATTACGTGTAGCATTCCAACTTCTAAAGAGCGTTTCGCTTCCCTTATTAGGGTTGCCAGCAACTGATACAGCTAAAGCAGCCGCTGTTGATTTACCAGTTGATGACTGACCTGTAAAACTAA

AGATGATACCAGCAAATGTAACATCTTGTTTATGCTTCAGAAATGCTGTTACTAAGGCAGAAATTCCGAATGTGGTTGCTAACTCCAAATGAAAATGACCTTTGACCTCATTAAGATACATATTAAACCAA

TCTTCAAACGTACCTTTAGGTTCTAAATCATAAGATGTATCACAAATTGGCTCTGAAGGAGTGTTAGCATTGAATTCTTTGGTCTTATAGACATCATTTAAGGATATAATTGAACCAAATGGAGTTTCTAGT

ATGCCTACACCTTCATACAAGTGGGAAGGTGATAATAGATTGCGCGTTTGTTGCAAGGCATTACTTAAAGACGTGATGCGCTTTTCGTTAATACTATAGCCGTAATTGATTAGTAAATGTAAATTGCGAGA

TGATAAAATATCGGACGCAAAGATATTTTCTTGACCATTAGCATTCGAGATAACTATTTTTTCAGTACCTTTCGAAGGATCTAGAAATTTTTTTGTAATCACAATAAAACTAGCCAAAAAAACTATTCTTTT

ACTATCTCCATCTTTTTTAGGTGGTATGTTTTCATACCAACCGTCTGAATTTAAAAAATAGGGTCCTATTTCAAGTATATTTTTTGACATTAGCGAACACCTCCTTTCGAAGTGTTGCTATGATTACGTGGAT

GTGGACCTATTTTTTGATATAACAAACTGCCATTTTTCGTACCTACAGTATCAAAGGGTAATCCTGGTGTGTGCTTAATCCAGTAAGCAAAACGACGTCCTACTTCACGTTGATCATTCGTGCTACAAACCC

CACTATATTTCTTTCTTAATTCACTATAAGTGAACATGCTTCCAACTGATTGTTGAAAAGCTTTGGCAATTACAAAATTATAAAGCTTCTCCTGTGATTCACTTTTATTTCTTTTCATAACTATTATCTACCTC
CTAAAATTATCACTTACGTGATATGGACTTTATTTTACTCTCAAAGAAAAATAGAGGTTTGAGATTTCGAAAGAGAATTTTAGGACATTGATTCTATAACGTTTAATAAATAAAAAGCCCAACGATTTAAG
AATCGTTGGGCATATTTATTGAATTAATAATATAAATTTGGTCCTTCTTTAATACTGTCTAAACGTGTCTTGTAAAACTTACTAATTATTTGAATCATTAGTTTATCGAGAAAGTCATTAGGATTATTATTAA
AATTTTCTCTTTTATAAAAGTTGCTAATGTTATATTTTTTAAGCTCAGTGAGTAAAGAGTCTTCTACTTTTTCTGTCATTTTTACATTATTTTTAATGGTTTTATGAATCAAAGCTTTCAAATTAGTATTTCTTG
CATAGCCATTAATAAGACTCTCAAAGTGAGTTAATTTTTGTTCATGTTTGTAGGTTAAGGCAATTAGATCATTCTTTAAATTAGTAATACTATGCAGAGCAACTAATATATCATATTGAGTTGATTTTTGAA
TGATATTACTTACTAGTGTAACTAATTCATCGATTAAATCGGAAATAGCAGTAAATAATGCAAAGTGGAATGTATCTTCAATGGTCGTTAAATAATATAATTCGTTATTATTAATTAAGTTTTGTTTTTTCA
TAAATTCATTTATATTATTTTGAGACTCAATCAGGCTATCTATTGTTAAATTAACTTTACGTGACTTATTTGTCAGAATACTACGGATAAATTTAAAATACGTATCATTTTGATCATATAAGTTGAGAGAAA
TACCGATAATAGCTTGTCTGAATTGATCTAGTTCGTCAGATTGATTTAAACTATCTTTTTTATGAGTTGAAGGTTTAATACTATTAGCAATTTCAAAGTATTGAGAATTACTTGTTCGTTCTTTAATTAAACC
TTCTGTCACTAGTTGTTTAAAGTACTTAGATTTATTTACGTTTGAGATGTTTTTCTTAACATAGGAATCTTGTTTTTCTTTGTAAATGAGATATTTTGTTAAAAAATTACTTGTAACTACATCGTCAAAATTTT
CTCTAGATAAATCTTGATGGATTTCTATTATTATATCTTTCAAATTTTTAAATGTATTCCAAGAAGATACGTTGTCTACAGTTTTTTCGTAATCTATTTTATATTTAGCTTTTTTTAAATTAATATTTTCTTGAA
TCTGTTTTATAGTATTGTTAGCTATCTTTTTAGCAAAATCTGTTGTTTTATTGATTGTGGCTAAGAACTCGGTGAAATATGTATCTAGAATTGTTGATTCTAACACTTCTAGAATATCTACACCTTGGTCAAT
GATTTCATTTGAAAGTTCATCTATGTCCACCATACTTTGACTATCAATAGAATACGTCAGATTATCTTTAGTAAAGCCACTAGGAATTTTATCGATAAATATACCCTCTTCACTACATTTCACAGTATGTTGT
TCAATATTTTGATTAGAGGTATTTACAAAGTAGCTTATATATATAGTTTCATTAGGTTGTGCTTCTCCCACTACATAATTTGTTTTAATTTCTTCAGGTAAAATATCTTTTGCATACTCTTTTAGTATAAATTC
GGAATTAATTTTATGTATAAAATAATTAAAACTTTGAAACCACATTTCTATTTTCCATTTATTTTTAATCATTGCATTCACTCGCTTTCATATTTAAAGTTTGTGTATCAACAAATACTAATTTCAATGTTAA
AGTTTTTCAATAGTATTTACTATTTATTCTTTATAATATGGAGAATAAAAAACAAACGTTCTCCTTTGAGCATATATATGAAAAAAGAGTCGAATCTTTTAATGAGTGACATCAAGTTCTTAAAAAATTATA
TAGATAATAGCTTATAATTATTGTTTAAAAACTATAGTAATTTGAATTGGAACTTAATTCTAAAAACTAGTCGGGCATCTAATAATTTTTAAATTAAATCGAAATTTCCGTTTATTTACTCTTGATTACTTCA
AAATATATTAATCTTACACATTATGAAATGATCGATAAAATGATATAAATATACAAATGAAATAATATGATAAAGTAAATGTAAACAAAATATCAGATTTAATTAACCTAGAATATATGTTATGTTAAAT
GTATATAACAATATATTCACTAGGGGTGCAATTGATTGCTGAGAGAAAGTACTTACTTTCAACCCTTGAACCTGTCGGTTAGTACCATCGTAGGAAAGTGATTTACATATTATTTGATGTTTTTCAGTAATT
CACTTTTTTGCGTAGAAAATTGAGTTATTGGAGGATTTTTTTATGGAGAAATTAAGAGTTGGTTTAGAATGGTTTTTAAATCCTGATCATATGCCATTAATCTTAGGAATAGAAAGAGGATGGTTTAAGGA
ACAAAATCTAGAAATTGAGATGATCGAACCTAAAGAACATTTTGATGCTTTAGATGAAATAGAAAAAGGTACAATGGATATCGCAATTACAGAACCTATCCATTTAGTAGAAGATAAAGCATCCAACCA
AAATGTTGTAGGCTTCGCAAGATTCTTACACACCAACGGCGGTATAATGTATAAAAAAGATAAAGGTATTAAAACACCAAAAGATTTAATTGGCAAACGTCTCCAGTACCCAGGAGCTCCAGGTCTTGGG
GGTATTGCTATTGCAAAAACAATGATTGAAGCTGATGGTGCTCAATATATAGATGGAGATATTAAACCTGTAAATAACAGTTTTTATCATACAGATGCATTATTAAATGATAAAGCAGATGCCGCAACACT
TATCTTTGAAAATTTTGAAATTTTAGAAGCCAAAAGTAAAGGATTAAATGTCGATTATTTCGCATTAAAAGATTACAATGTTCCTGACTTTTGCCAATTAATTTTTATCACTACCCCTGATAAGTTACACTTT
GAAGAAGAAAAGATCAAAACATTTATTAAAGTTATACAAAAAGCAATTCAATATATTAAAACTAATCTTGAAGATGCAATTCATATCTACTCTTCATATACAAATACAGATGTAAGCGATAATTTAAATA
AATCTACAATACAAGCCACAGCAAAATGTTTCACTAACGATTTATCAATGAGTTCTGATTTCTACAATGATTTACAATTATGGTTAAAAGAAACAGGTAAGATTAAACAAACAATCGAACCTAAAGAATA
TTTCACAAATCAATTACTATTTAGTAGATATGCTAACTAAATTCAAGTTTATATTCAGAGTCTCTACAATTTTTAAATTGTAAGAGGCTCTTTTACTTCTAAAATATTGCCATTCTCTTCAATATCTAAATAT
ATTTCTTCATTTTTTGCATTGCCGCCACCTAGTCTTTGCAATAACTGAACTACTTCATTTTTCCTTAATTGAGAAAGAACATGTTTAGAAACATAAAGGTTCTGTTGCAAAGTAAAAAAATATAGCTAACCA
CTAATTTATCATGTCAGTGTTCGCTTAACTTGCTAGCATGATGCTAATTTCGTGGCATGGCGAAAATCCGTAGATCTGAAGAGACCTGCGGTTCTTTTTATATAGAGCGTAAATACATTCAATACCTTTTAA

AGTATTCTTTGCTGTATTGATACTTTGATACCTTGTCTTTCTTACTTTAATATGACGGTGATCTTGCTCAATGAGGTTATTCAGATATTTCGATGTACAATGACAGTCAGGTTTAAGTTTAAAAGCTTTAATT

ACTTTAGCCATTGCTACCTTCGTTGAAGGTGCCTGATCTGTAATTACCTTTTGAGGTTTACCAAATTGTTTAATGAGACGTTTAATAAACGCATATGCTGAATGATTATCTCGTTGCTTACGCATGTATGTCC

CTCTGTATCAATGGCACGATATAAATAGCGCCATTTTCCTTTTATTTTGATGTACGTCTCATCAATACGCCATTTGTAATAAGCTTTTTTATGCTTTTTCCTCCAAATTTGATATAAAATTGAGGCATATTCTT

GAACCCAACGGTAGACCGTTGAATGATGAACGTTTACACCACGTTCCCTTAATATTTCAGATATATCACGATAACTCAATGCATATCTTAGATAGTAGCCAACGGCTACAGTGATAACATCCTTGTTAAAT

TGTTTATATCTGAAATAGTTCATACAAAAGACTCCATTTTGTTAAAATCATATTATAAATTCAACTTTGCAACAGAACCTCTTTTCTAGTATAATGTTTATTAATATAGTACATATTTTTATAAGGAGTTGTA

CGCATGTATAATCCATTCGACGAAGCTTATCATGGCTTATGCGAAGAGATATTAGAAATAGGGAATAGACGCGATGATCGCACACATACCGGGACAATTTCCAAATTTGGGCATCAATTGCGTTTCGATTT

AACCAAAGGGTTTCCTTTACTTACAACCAAAAAGGTTTCTTTTAAATTAGTAGCAACTGAATTATTATGGTTTATTAAAGGAGATACAAACATTCAATACCTACTTAAATATAATAACAATATATGGAATG

AATGGGCATTTGAAAATTATGTTCAGTCTGATGATTATCATGGACCAGACATGACAGATTTTGGACATCGTTCCCAGCAAGACCCTGAATTTAACGAGCAGTATAAAGAAGAAATGAAAAAATTTAAAGA

ACGTATTCTAAACGATGATGCATTTGCTAAAAAATATGGCAACTTAGGGAATGTTTATGGTAAACAATGGAGAGATTGGGAAGATAAAAATGGAAATCATTACGATCAATTAAAATCAGTTATACAACAA

ATTAAAACCAATCCAAACTCTCGACGTCACATTGTTTCAGCATGGAATCCTACTGAAATCGATTCAATGGCATTACCACCTTGCCACACAATGTTTCAATTTTACGTACAAGAGGGTAAATTAAATTGTCA

ATTATATCAAAGAAGTGCTGATATATTTTTAGGTGTTCCTTTCAATATTGCAAGTTATGCTTTACTGACACATCTTGTTGCAAAAGAATGTGGACTTGAGGTGGGTGAATTCATTCATACATTTGGAGATGC

ACATATTTATTCCAATCATATGGATGCAATACATACACAATTATCAAGAGATAGTTATCTACCACCTCAATTAAAAATTAATACAGATAAATCAATTTTTGATATTAATTATGAAGATTTAGAATTGATAA

ATTATGAATCACACCCTGCTATTAAAGCACCTATTGCGGTATAAAAAGATGCATTAATATTTCGCATACAGAAAGGAGGTATACCATGACATTATCAATAATTGTCGCTCACGATAAACAAAGAGTCATTG

GGTACCAAAATCAATTACCTTGGCACTTACCAAATGATTTAAAGCATATTAAACAACTGACCACTGGGAATACACTTGTAATGGCACGGAAAACTTTTAATTCTATAGGGAAGCCATTGCCAAATAGACG

TAACGTCGTACTCACTAACCAAGCTTCATTTCACCATGAAGGGGTAGATGTTATAAACTCTCTTGATGAAATTAAAGAGTTATCTGGTCATGTTTTTATATTTGGAGGACAAACGTTATACGAAGCAATGA

TTGACCAGGTAGATGATATGTATATCACAGTAATAGATGGAAAGTTTCAAGGAGACACATTCTTTCCACCATACACATTCGAAAACTGGGAAGTCGAATCTTCAGTAGAAGGTCAACTAGATGAAAAAAA

TACTATACCGCATACATTCTTACATTTAGTGCGTAGAAAAGGGAAATAGGAGGCAATTATGGCTAAACAAATTATCGTCACGGATTCAACCTCTGATTTATCACATGAATATTTAAAACAACATAACATTC

ATGTTATACCATTAAGCCTGACAATCGACGGGAAATCTTACACTGATCAAGTTGATATCTCTTCAAGTGAGTATATCGATCATATTGAAAATGATGCAGACGTCAAAACAAGTCAACCACCTATTGGTCGA

TTTATTGAAACATATGAGCAATTAGCTCAAGATGACGTTGAAATTATAAGTATTCATCTTTCGTCAGGCTTAAGTGGTACTTATAATACTGCTGTTCAAGCGAGCCATATGGTAGATGGTAATATTACAGT

GATTGATTCTAAATCTATTTCGTTTGGGTTAGGTTATCAAATTAAGCAAATTGTTGAATTGGTTCTGTTGCAAAGTAAAAAAATATAGCTAACCACTAATTTATCATGTCAGTGTTCGCTTAACTTGCTAGC

ATGATGCTAATTTCGTGGCATGGCGAAAATCCGTAGATCTGAAGAGACCTGCGGTTCTTTTTATATAGAGCGTAAATACATTCAATACCTTTTAAAGTATTCTTTGCTGTATTGATACTTTGATACCTTGTCT

TTCTTACTTTAATATGACGGTGATCTTGCTCAATGAGGTTATTCAGATATTTCGATGTACAATGACAGTCAGGTTTAAGTTTAAAAGCTTTAATTACTTTAGCCATTGCTACCTTCGTTGAAGGTGCCTGATC

TGTAATTACCTTTTGAGGTTTACCAAATTGTTTAATGAGACGTTTGATAAACGCATATGCTGAATGATTATCTCGTTGCTTACGCAACCAAATATCTAATGTATGTCCCTCTGCATCAATGGCACGATATAA

ATAGCTCCATTTTCCTTTTATTTTGATGTACGTCTCATCAATACGCCATTTGTAATAAGCTTTTTTATGCTTTTTCTTCCAAATTTGATATAAAATTGGGGCATATTCTTGAACCCAACGGTAGACCGTTGAAT

GATGAACGTTTACACCACGTCCCCTTAATATTTCAGATATATCACGATAACTCAATGCATATCTTAGATAGTAGCCAACGGCTACAGTGATAACATCCTTGTTAAATTGTTTATATCTGAAATAGTTCATAC

AGAAGACTCCTTTTTGTTAAAATTATACTATAAATTCAACTTTGCAACAGAACCGTATTATGGAATAGAGATGTTGGTAACATTTATACAGGATCATTATACTTAAGTTTAATTTCGTTATTACAGAACCAC

ACATTCCAACCAGAAGAGAAAGTATGTCTATTTAGTTATGGTTCAGGAGCAGTAGGAGAAATCTTTAGTGGTTCAATCGTTAAAGGATATGACAAAGCATTAGATAAAGAGAAACACTTAAATATGCTAG

AATCTAGAGAGCAATTATCAGTCGAAGAATACGAAACATTCTTTAACAGATTTGATAATCAAGAATTTGATTTCGAACGTGAATTGACACAAGATCCATATTCAAAAGTATACTTATACAGTATAGAAGA

CCATATCAGAACATATAAGATAGAGAAATAAACTAGTGGCCGATTGTGCTTGATGAGCTTGGGACATAAATCCTAACTCGAAATAAATAAGCATATCACTAAACTGATTTTTTAAAGTTTACAGTGATAT
GCTTATTTTTTTATCTTACGATTTTGTACGTGCATGCTTGCCTAGGGGTATGGCTCGAGCCATTAGTCTCTCGCACATACTATTCCCTCAGGCGTCAGCACTTACAAAATCGGTTGTAATTTTCATTTTTATA
CGCATTCTTACTGAGATTATACTAATAAGAGGAATAGTAAAAGCAATTCTAAGTAAAATTGCAGATAAGAGGTTTGTTAAAAGCAGTTCTAAGTAAAATTGCAGATAAGAGGTTTGTTAAAAGCAGTTCT
AAGTAAAATTACAGATAAGAGGTACGTTAAAAGCAGTTCTAAGTAAAATTGCAGATAAGAGGTTTGTTAAAAGCAGTTCTAAGTAAAATTGCAGATAAGAGGTACGTTAAAAGCAATTCCATGCAAAAT
TGCTGATAAGGGGTAAGTTAAAAGCAGTTCTCAGTAAAATTGCAGATAAGAGGTACGTTAAAAGCAGTTCTAGGCAAAATTGCAGATAAGAGGTGCGTTAAAAGCAGTTCTCAGTAAAATTGCTGATAA
GGGGTAAGTTAAAAGCAATCCTAAGTAAAATTGCAGATAAGGGGTACAGAAAAACTAGACTTGATTACAAAATGGAGCTTGGGACATAAATGATTTTTTAAAAATGAGATGAGACGTAGATTAACTCCA
TAATCAATACGAATCTATCGACTTCTTTATTTATGATATTCATCTCTTTTTAATGGAAATAAAAGTGCGATTAATGTGATAATACAGTTACGTTAATTAAAAAAATAAAAATGCAAGGAGAGGTAATATGC
TAACTGTATATGGACATAGAGGATTACCTAGTAAAGCTCCGGAAAATACAATTGCATCATTTAAAGCTGCTTCAGAAGTAGAAGGTATAAACTGGTTGGAGTTAGATGTTGCAATTACAAAAGATGAACA
ACTGATTATCATTCATGATGATTATTTAGAACGGACTACAAATATGTCCGGGGAAATAACTGAATTGAATTATGATGAAATTAAAGATGCTTCTGCAGGATCTTGGTTTGGTGAAAAATTCAAAGATGAAC
ATTTGCCAACTTTCGATGATGTAGTAAAAATAGCAAATGAATATAATATGAATTTAAATGTAGAATTAAAAGGTATTACTGGACCGAATGGACTAGCACTTTCTAAAAGTATGGTTAAGCAAGTGGAAGA
ACAATTAACAAACTTAAATCAGAATCAAGAAGTGCTCATTTCAAGCTTTAATGTTGTGCTTGTTAAACTTGCAGAAGAAATCATGCCACAATATAACAGAGCAGTTATATTCCATACAACTTCGTTTCGTG
AAGACTGGAGAACACTTTTAGATTACTGTAATGCTAAAATAGTAAACACTGAAGATGCCAAACTTACTAAAGCAAAAGTAAAAATGGTAAAAGAAGCGGGTTATGAATTGAACGTATGGACTGTAAACA
AACCAGCACGTGCAAACCAACTTGCTAATTGGGGAGTTGATGGTATCTTTACAGACAATGCAGATAAAATGGTGCATTTGTCTCAATAGAAAGTTAGAGGTGAGTCTTACGTTTCAGTGACGGTAGACTT
ACCTTTAACATGTTACATACTAAAAAATTAATTTGAATAAGAAAGAGAGACATATATGAAATACGATGATTTTATAGTAGGAGAAACATTCAAAACAAAAAGCCTTCATATTACAGAAGAAGAAATTATC
CAATTTGCAACAACTTTTGATCCTCAATATATGCATATAGATAAAGAAAAAGCAGAACAAAGTAGATTTAAAGGTATCATTGCATCTGGCATGCATACACTTTCAATATCATTTAAATTATGGGTAGAAGA
AGGTAAATACGGAGAAGAAGTTGTAGCAGGAACACAAATGAATAACGTTAAATTTATTAAACCTGTATACCCAGGTAATACATTGTACGTTATCGCTGAAATTACAAATAAGAAATCCATAAAAAAAGA
AAATGGACTCGTTACAGTGTCACTTTCAACATACAATGAAAATGAAGAAATTGTATTTAAGGGAGAAGTAACAGCACTTATTAATAATTCATAATAAAACAGTGAAGCAACCATCGTTACGGATTGCTTC
ACTGTTTTGTTATTCATCTATATCGTATTTTTTATTACCGTTCTCATATAGCTCATCATACACTTTACCTGAGATTTTGGCATTGTAGCTAGCCATTCCTTTATCTTGTACATCTTTAACATTAATAGCCATCAT
CATGTTTGGATTATCTTTATCATATGATATAAACCACCCAATTTGTCTGCCAGTTTCTCCTTGTTTCATTTTGAGTTCTGCAGTACCGGATTTGCCAATTAAGTTTGCATAAGATCTATAAATATCTTCTTTAT
GTGTTTTATTTACGACTTGTTGCATACCATCAGTTAATAGATTGATATTTTCTTTGGAAATAATATTTTTCTTCCAAACTTTGTTTTTCGTGTCTTTTAATAAGTGAGGTGCGTTAATATTGCCATTATTTTCT
AATGCGCTATAGATTGAAAGGATCTGTACTGGGTTAATCAGTATTTCACCTTGTCCGTAACCTGAATCAGCTAATAATATTTCATTATCTAAATTTTTGTTTGAAATTTGAGCATTATAAAATGGATAATCA
CTTGGTATATCTTCACCAACACCTAGTTTTTTCATGCCTTTTTCAAATTTCTTACTGCCTAATTCGAGTGCTACTCTAGCAAAGAAAATGTTATCTGATGATTCTATTGCTTGTTTTAAGTCGATATTACCATT
TACCACTTCATATCTTGTAACGTTGTAACCACCCCAAGATTTATCTTTTTGCCAACCTTTACCATCGATTTTATAACTTGTTTTATCGTCTAATGTTTTGTTATTTAACCCAATCATTGCTGTTAATATTTTTTG
AGTTGAACCTGGTGAAGTTGTAATCTGGAACTTGTTGAGCAGAGGTTCTTTTTTATCTTCGGTTAATTTATTATATTCTTCGTTACTCATGCCATACATAAATGGATAGACGTCATATGAAGGTGTGCTTAC
AAGTGCTAATAATTCACCTGTTTGAGGGTGGATAGCAGTACCTGAGCCATAATCATTTTTCATGTTGTTATAAATACTCTTTTGAACTTTAGCATCAATAGTTAGTTGAATATCTTTGCCATCTTTTTTCTTTT
TCTCTATTAATGTATGTGCGATTGTATTGCTATTATCGTCAACGATTGTGACACGATAGCCATCTTCATGTTGGAGCTTTTTATCGTAAAGTTTTTCGAGTCCCTTTTTACCAATAACTGCATCATCTTTATAG
CCTTTATATTCTTTTTGTTTTAATTCTTCAGAGTTAATGGGACCAACATAACCTAATAGATGTGAAGTCGCTTTTCCTAGAGGATAGTTACGACTTTCTGTTTCATTAGTTGTAAGATGAAATTTTTTTGCGA
AATCACTTAAATATTCATCCATTTTTTTAACGGTTTTAAGTGGAACGAAGGTATCATCTTGTACCCAATTTTGATCCATTTGTTGTTTGATATAGTCTTCAGAAATACTTAGTTCTTTAGCGATTGCTTTATA

ATCTTTTTTAGATACATTCTTTGGAACGATGCCTATCTCATATGCTGTTCCTGTATTGGCCAATTCCACATTGTTTCGGTCTAAAATTTTACCACGTTCTGATTTTAAATTTTCAATATGTATGCTTTGGTCTT

TCTGCATTCCTGGAATAATGACGCTATGATCCCAATCTAACTTCCACATACCATCTTCTTTAACAAAATTAAATTGAACGTTGCGATCAATGTTACCGTAGTTTGTTTTAATTTTATATTGAGCATCTACTCG

TTTTTTATTTTTAGATACTTTTTTTATTTTACGATCCTGAATGTTTATATCTTTAACGCCTAAACTATTATATATTTTTATCGGACGTTCAGTCATTTCTACTTCACCATTATCGCTTTTAGAAATATAACTGCT

ATCTTTATAAACTTGTTTGAAATTTTTATCTTCAATTGCATCAATAGTATTATTAATTTCTTTATCTTTTGAAGCATAAAAATATATACCAAACCCGACAACTACAACTATTAAAATAAGTGGAACAATTTTT

ATCTTTTTCATCAATATCCTCCTTATATAAGACTACATTTGTAATATATTACAAATGTAGTATTTATGTCAAAATAATGTTATAATTTTTGTGATATGGAGGTGTAGAAGGTGTTATCATCTTTTTTAATGTT

AAGTATAATCAGTTCATTGCTCACGATATGTGTAATTTTTTTAGTGAGAATGCTCTATATAAAATATACTCAAAATATTATGTCACATAAGATTTGGTTATTAGTGCTCGTCTCCACGTTAATTCCATTAATA

CCATTTTACAAAATATCGAATTTTACATTTTCAAAAGATATGATGAATCGAAATGTATCTGACACGACTTCTTCGGTTAGTCATATGTTAGATGGTCAACAATCATCTGTTACGAAAGACTTAGCAATTAAT

GTTAATCAGTTTGAGACCTCAAATATAACGTATATGATTCTTTTGATATGGGTATTTGGTAGTTTGTTGTGCTTATTTTATATGATTAAGGCATTCCGACAAATTGATGTTATTAAAAGTTCGTCATTGGAAT

CGTCATATCTTAATGAACGACTTAAAGTATGTCAAAGTAAGATGCAGTTCTACAAAAAGCATATAACAATTAGTTATAGTTCAAACATTGATAATCCGATGGTATTTGGTTTAGTGAAATCCCAAATTGTA

CTACCAACTGTCGTAGTCGAAACCATGAATGACAAAGAAATTGAATATATTATTCTACATGAACTATCACATGTGAAAAGTCATGACTTAATATTCAACCAGCTTTATGTTGTTTTTAAAATGATATTCTGG

TTTAATCCTGCACTATATATAAGTAAAACAATGATGGACAATGACTGTGAAAAAGTATGTGATAGAAACGTTTTAAAAATTTTGAATCGCCATGAACATATACGTTATGGTGAATCGATATTAAAATGCTC

TATTTTAAAATCTCAGCACATAAATAATGTGGCAGCACAATATTTACTAGGTTTTAATTCAAATATTAAAGAACGTGTTAAGTATATTGCACTTTATGATTCAATGCCTAAACCTAATCGAAACAAGCGTA

TTGTTGCGTATATTGTATGTAGTATATCGAGCTTCACATGAAACAGCTAAAGAAGCTTTGGGCGATAAAGAGTTAAGAGCCATTGCACATGAGTTAACTAAAACAGTTAAGGATAACATGAGTGTTGATT

GGTCTAAACGAGACAGTGCTAAAGCTAAAATGAGAGTTCAAGTTAGACGCCTATTAAAGAAATATGGCTATCCACCAGATCTTCAAAAAATGGCTGTGGAACAAGTTGTAGAGCAAGCAGAATTAATGG

CAAGTCAGCAATAAAAAAATAAATCATAATGAGTCCGGGACATAAAGTTCTTGGATAAGTGAAAAAAGACAATTTCTATTGAAATAATATAGAAATTGTCTTTTTTATAAATTTTTTGATTATTTTCAGCT

CGTTGAGCTACTACTTTTCTTATATTAAGTGCCATTAATACAAAACCAAGTTCTCTTTTGACTTTATTGAGTCCTCGGACAGACATCCGAGTGAAACCCAAAATAGCCTTCATAAATCCAAAAACAGGTTCC

ACATCAATTTTTCTTTGACTGTAGATATTTTTTGTTTCTGGTTCTGAAAGCTTTTTGTTAATTTGGGATTTAAAATATTCCCAGTTATAATTCTTCATTATTTTTTTGTTTGTTTTTGAATTGAAGTTCATACAT

TGATTTTTCAGAGGACATTCTGAACAATCATCACATTCATATAATTTGAAGTCTCGCTTATAACCATACTTATCATGACGATAGGCATATCTTTTAAAACCTAGCCGTTTATTATTCGGACAAATGAATTCG

TCATTAATTTCGTCATAGTTCCAATTTTGAGTATTAAAGATGTCACTTTTATATTTTTTAGTTTTATCTTTTATAAACATTCCATATGTTATGAGTGGCGTTCGATTAAAGTCATCTATAATTGCCTTATAATT

TGATTCACTACCATAACCTGCATCAGCTACAATATATTCAGGTAAATGACCGTAGGTCTCTTGAATTGAATTTAAAAATGGAATCATCGTTCTAGTATCCGTTGGATTTTGATACACATTATAAGATAAAAC

AAATTGGGAATTTGTTGCTATTTGTAAATTATACCCTGGCTTAAGTTGTCCATTTTTCATGTGATCTTCTTTCATTCTCATAAATGTCGCATCATAATCTGTCTTAGAATAACTATTTCTATCCTTTAAAATAG

ATTTTTGAAATTCGTATCGATACTTTCGCTCAAAATAATCATTGATTTGCTTTTTGTATTTTTTGATTTTAGTTCTTTTGAGACGTATTTGTTTTCTTGTTTTAGTACATTTTTCATTGTTGATATGTTGGTTTAA

ATCTTCGATTTCTTTATCTAAGTGACTACCAATCAAATCTATTTCTTCTTTTGTTAATTCATTATCATGATCTTCTTTAATTTCCGGTATGATTTTATTGGTTACCAATTCATGGTAGAGGGCTTTAGAATCCT

CATTCATCTTTGATTCATGGTTTTGAATACTCTTTTTCCATACAAATGTATATCGATTGGCATTTGCTTCAATTTTTGTACCATCAATAAAAATAGCTTTATCATCTATAAGATTTTGTTTTACACACTGACTG

TAAAATTGAATAAATAAAGATTCTAATAAAGCATCTACTTTTGGATTTACTCTAAATTGATTAATTGTTTTATAAGAAGGTTTTTGATTTTGTGATAGCCACATCATTCGGATGCTATCATTAAGCATTTTTT

CTATTTTACGACCTGAGAATACAGATTGTGTGTAGGCATATAGAATCACTTTTAACATCATTTTAGGATGGTACGAAGTTGCACCACGGTGATGTCTGAATTCGTCGAATTCATTGTCAGGAATTGTTTCAA

CAATATCATTTACAGTAAAACGATGTTGATTTGTTTTGTTTCCATATTGACCTCCATGTATTTGCTATGATTTCAAAATCCATTTTTGACGTGCCTTAGGGTTGAGTGGATGCATAATTTCATTTGTTACTGG

ATTGATGAGCTTTTTTACTTTCTTTTTATGAGGTTTTAACATTTCCATCACTTGTTCGACACGGTCGATAACAACTGGTCGCTTCGCATAGGCACCATAAGCAAGAATCACTGTGTCACTTTCACTAATCGCT

TTCATCAAATGAATATCAGTGTGCTCATCGTATGGATTTTTGATATGTTTGAGGTTTTCGGGTGTTCTAATATTTGAAAATAGATTTACAAGATATACAGCACCATATTGTTCAGTATTAGCTAATTGATTG

AGGATAAGAACAGTTGTGAGATCGAGTGATAATACACCATCTAAATGAGGGTACATCGTTATCACTGTACAAGCGGGTTTCTTTTCATCCCATGTTTTCTTGAGTAAATAGCGGTGTTGTTCATCATCGCTA

AATATGGCTTCTGTGTTTATCGTACTTTTGATTGTATTCATCATCGTCACTCCTTTTAGTATTCTTCTGGTAAAAGCATCACATAATAAAAAACATCTACATCATCTTCTCGGATGACGTAGACTTTCTTAGG

TAATGCATTTTGATTTTTTATATAGTTTGTATAGTGATATTCCAATTTGTACGTGGGTTGTTCTTGCTCATGTATGATTGAAAGTATATTCTCATCTTCTTTCAGTCTAAAAATGTGTAGGTAATCTGTATCAG

GTTGATTATCTCTTTCTTTTACCATGTTCCAAAGTAAGATTTGAAGATCTAGAGATAGGTGTTCACTAATGCCTCTTGTGATATATCGATTGATGTTCATACTATTTTCCTCCATTTTGCTTTTCTTTCATGAT

GTCAATCACTTCGTTAATGACTGTAACAGATATTTGTGCCACTTTGATCCAATTATTCATGGTCCTGACCTCCTTGTTTTAGTAAATGACGTTCATCAATAATGATATTTTGAGTATCTGTAAGGTACAGAA

AGTCCATATCAAAATGATCCAGATAACCAATACTGATGAGTTGGTTATTGGCATACATTAGAAATGGATAGATACTTAGCTCATGTAGCTCATCATTATAGTAGGTATAAGTCTCGAGTGTGAGTTGTACC

AGTGGAGAATCATTAATAAAACGTTCCGGTAGAATATTTTCTGCTGTTTCCTCCAGCGCTTCACATTCCCAAGCTTCGTTATTAGATAGTTGGAATAAGTGGGTCATATATTGTTTGAGTTCTTGAGTGATG

GTTTTCATATTATTGCCTCCTAGATAGTGTAATAGTGATGTAGTTCATATACATCATTGAGATAATATATATTTGATTTGTCATTTATTACGAATCCCGGTGGGAATAAGAGAAAATTCCATATGAAAAACC

GCTTCAAACCTTGGTATGACAAGGAAATCCCGAAATTCCGCCTGTTTTGACGAACAATCAACTCATTATTTATAAGTATTGATGATAGTGGCGGGTCTTTACTTCCTTATATATATTATTTATTTATAAAAA

ATAACGGGATTTTGGGATTGTGCTTGCACAATCCTTCTGCTTCTTTGAATCTGCAAATCCCATTCCTTTCCCGTTAAAAAATCATTGTGGGATGTTCTTTAGCAATTTCAATATAAGCATCGTGTAGTTATGA

AAAAAATTACGATAATGACTATTTCATTAGATAAGTGTTATTGAAATTGATAAAGAGAATTCTTAAACTTACTTAGAAACTTTAAAGAAAGAAAAAACGAAGTGCTACAAGGCACTTCGTTAATATTACA

ATTGGTATTATTAATCTTCTTCACTAGAAATGTTGATATCGAATATACAGATTTAGAGCTATCTACATTTCCACGTGGATTATTTGGATTATAACCTACGAACGCTTGTTCATCTTTAGATAAATTATCTAAT

TTAATATACTTAATATTTTTATTGCAGCTTTTATTGTTCTTCCTCACTGTCCTCTTCGCCATATATTTTTAATCTTCCTTGAGACTCTAATAAATCTGTAGTTACTGTTCGTTCGCTATCTTCAATTTTTCCATCT

CCATCTTCATCAAATTGCTCCGGATTTTGAGCTGCTTCATCAAGAGATCTATTTACAGCATCTTGATTAAAATTATTTTTTCCAGGTGGTTCTTCTTGTCCCTCTGTTTGCTGAGTAGTGTTATTTTCACTAGT

GTTATTAGTTTGAGCAGTGTTTTGTTCATTGTTATTATTGTTTTGCATAGTATTTTCTTGTGTATTATTGTTCTGAGAATTATCTTGTTGAGTTGTGTTATTATTTGCAGATTCATCGTCAGAAGAGATGTTAC

TTTCACTATTGTTCATTTCTTGTGAATTACTATTTTCATCTTTATTAGTTTTATTTTCTTTTTTATCTTTTTTTGTACTTTGATGAGTAATATTTTGGTCTTCTTTATTTTCATTATGATTAAAAGACTCTTTAGCA

AAATTTACTACAAATAGTGCTAAACCAATAAATATGATTGATAGTATTGCAATCAATAGAATATTAATAATTTTTTTCATTCATTTTTCCCCTTAATTTGTAATATTTAAAATTTAATAAATTAATTTTATCT

AATTTTTATAATTGCATCTACTATATTTTTAATTATTTATTATTTTAGTTAATATTTATACTCACTTCTTTTATTAAATAGTAAGATATAATGTTTGGGATAAAAATAAAGACGAAGTGCTAAGGAGCACTTC

GTCAAGTGGATGATTATTAAATAGTTGTTTGATAATATCATTATTTAGTTTGAGCGTAACATAAAATTGCTTCTTATGCTGTTCATCTCTTCGGATATCAATACGATCGATGACTGTTAGATATAGTGATTTC

AATTGAGATTTCTCTAATTTGTCTATATCTTTGAATATTGTTTGTAGTATATTGGCAATCATATCAGCATCATAATGTGGAGCTTCCACTTGTTTATCTTGTTCTAGTTGATGGATTTGATTGTTTATTTGATT

CAACTCATCTTGATAGCTAAGTATTGTTGGTTTCAACACACTATCTAAGTCAGGTGAGTCTTCGATCGTTTTCGTTAGTGTATGCATTTTGGCTTTGATTTCTTCACATTGTGACTGTTTATAAGCGATGTCA

TGATTCAAAGAAGAGACATCTATTTGACTTCTTTCATTTACTTTTTCAACCAATTGTTTCATTACCTTTTTACTTTTGATAATCTCCAATATTTGGTTCATAACATATTTTTCCAATATATCTGCTCTAACACT

ATTGGCTGAACAAACTTTAGAACCTTTGTTTCTAAAATTACTACATGAGTAATATCTGATTCTTTTCTTAGTGCCATCTTTTAATGTATTGGTTGTATTACTTGCCGCCATTGCTGCACCACATTTCGGACAT

TTTACAATGCCAGTCAGTAGGTTTGTCCCTTTACCATGGACTTGTGGTTTCTTGCGACTCTCCTGGCGTTTAAACTGTACTTTATCCCATAAGTTTCTATCAATAATAGGTGCATGCTTACCGTCAGCGATAA

TAGGTTCCTCATTGAGTCCTTTTCGTCTCTTATCGCTCCAGTGTCTATACTTCGCAAACTGTATTTTTCCAATGTAAAAAGGGTTTGAGATGATGTAGGTAATGGATGAAATACTAAACGGTTTACCTTTCTT

AGTTACATAACCTTTGTGATTTAATGCATTCGCAATCTTACGATAACCATGACCTTTAGCGTATGACTCAAAAATATATTTAACAATATTTGCTTCATGTTGATTGATCATGAGCTCTTTTTTACTGTCAGGT

ACTTTATCATAGCCTAGAGGCAAATTACCTTGGTAATAACCTTCAATGGCACGTTGTCTTTGACCATTAAACACGTTTTCTACAATAACGTTTCTTTCAAATTCTGCGAAGCTCGCTAAGATTTGGAGCATC

AATTTACCTGTGGAGCTCGCAATTTCTATTTTTTCAGTTAGACTAAAAAATTCAACATTAATCTCATACAATTCTTCGACAATATTTAATAAATCTGAGGTATTTCTAGCTAAACGAGTAGTATTATATACC
ATAATACCATCTAATTTACCTTCTTTAGCATCTTTTAACATACGTTGTAATTCAGGACGTTGTGTTGTTTTACCTGATATACCACGATCGGTATATTCATCAACGACTTCATAACCTTGAAATTGACAATACT
CTGTAAGTTGATTTAATTGACCATGAATACTGTAACCATCCGTTTGCATTTCAGTTGATACACGTGCATATAATCCAATACGTTTCTTTTTGAGTTGTTTCATACGACTTCATTCCTTTCAGATATTATTAAG
TGTGATTGTTCAACGATATTGAGTGGACTATTTTTAAAGTAGATTCCTTGTAATTGTTTAGTTTGAGTTATGTTGATGCAATCGATAAAAGGTGCTATGGTTTCTAATGTCAATTTATCTTTGATTATATGAC
GGATTGTTTTGGTAATTTGTTTATGTGAATAATTAGAGTAACGTTCTTCACTTTCAGAGCCAGCAGATAAATGTTTAAACGTCTCGATATCAATTTGATTTTGTGCCAGTTTTTCGATGAGTTGTTCTTGTGT
TAGATGTGATTGTCTGTGTTTCATTTGTTGGTGTTTTAGAGCTTTTAGTATTGTCTCATTTAATTTGTCATGAAATGACTGATTGTCACAATATGCTTTACAAGTGTTGAGGACCTCTGTTTCAATCATTTGCG
CATTGATACCTTTAAATGGACACGTATGATAGGCTTCATTCATATTTTTAGGACAAACGTAGTAACGTAGAGAGTGTTTCTCTTTTTTTATCGTTAAGTTTGTTAATGTTGATTGACAGTAAGGACATTTGAT
TCGTCGCTTTAGCTTATTTCTAGAATTGGATCGATTGTGTTGTTTGTGAATACGACGCTCTTGTGCTTCTTCAAACGTATCAACATCAATAATAGATGGAACGATATCATTAAACGTTCCATATTTATTAATG
ACACGACCACAATAGTTAGGGTTCAAGAGAATATTTCGAACTTGATAGGGCTTACGAGGAATAAGCTTAGGATGGTTATCTAAATGTTGGGAAATCTTTTTGTAGCCTATACCCTGTAAGTACCAGCGATA
GACCGCTTTAACTGTATACGCTTCTTCTTCATGTACAATAAAATGACTTTGTCTATAACGATAGCCAAACGGAGCATGAGTTGTGATTAGCTTACCTTGTTTGGCTTTTTCTCTAATCCCATTTTTTGTTTGTT
CGCTGATATTGTTTGATTCCATTTCCGCTAGACTCATAAGTATGTTCAAGCGAAAACAATCAAACTCTTTAGCTAAGTTAAAATAGCCATCGTTAACACTGATAATCGTGATATGATGTGTTTTACAAATTT
CAAAGAATTGTATGGCATTTTTCAAATTACGATGGAGCCGATTTAGGCGATAGCAACACAATACTTTACATTTTCCAGAACGAATCGCTTCTACCATTTTTTGATAGCCCGAACGTTTTGTATGTCGACCTG
TTTTCTTATCATCATAAAACGTCACATTAGACCATCCATATTGCTTAGCGGTATCCATAATGAGCGATTTTTGAGTAGCTAAGCTTTGTTGTTTGAGTGTACTTTGACGTACATAAGCAATCGCTTCTTCCAT
GTTATACACCTCCAAAAAGATAATATATATTTGTGAGTAAATTAGAATGAAAGGTCCAACGTGCTTTTAACACGTTGGACCGCAATGATTAATTATCGTTCTGTAACTCTTCAACGATTAAATCAGCTAGT
AATTCAATCAATTCATCCATTTTATCTACTCCTGTACAATTGCATCTTTAAGTTATTAAAAAGGTATTGAGAAGTGGTCATTTAAAGATCAGCTAACTCATCATTTTGCTTTTTCCATAACTTAAAATTCTCT
TTGTTATCCGTATTAATTTTAGTTTTAACAGGTTCAGCATCTTTAGTTAGACCAAAAATAGACGCATATTCTGAATCTAGCTTTAAATGGTAAAAGACAAGTGACTGTTTTTTGCCATTGTCATCTTTGACTG
TTCGTTTTGTTGTAATTCGATCATGGTCAGATTCGATAAATCCTTTATCCCTTAGTGCATTAACAACATTATTGACATCTTGGAAGTGATTCTCTATCAACATATCTTTAAATACAGATGCAATGATTTTGAC
TTCGATATAATCATCTTTTAAGGCAATTAGTCCATGGTTCTCAATCATATTCTTTAACGCTGTATCATCAGAAAACTTACCACGGTTTTGTGCTACAAATTGCGTAATGACTTCGATCGCTTTATCTGCCAGT
GAGCGTTCAGAGACTGTATGACCATGATAATCAATAAAGTAGTCTCTTATTTTAGCGATATCAATATCTGTAGCTAAAACACGACTTAGAATCTTGGCCGAAGTAGTGATAGCCGCATAGCGCTTAAACAT
ACGAATACCAGTATTATTTTTTTCATCCTTCAATTTATCTTTAAACCAATGATGCTCATCTTGAAACCATTGAATGACTTCATCTTCACGGTTGATAAGATATTTAGCTACTAACGGTAAAACATGACCATA
GTTTAGTGCCACAGATTTTTTAATATTGTCAGAATTTGTTGCATCTTTAGTAAACCGTTCATTAATTTCAATAGTTCTCACGCGTAAACCATCATTTTGAGCAGAATCAGTAAAGATACTGTATTCAGAGGT
TGAAATCACAGAAGTGCCCCAATTCTTAGGCGTTTTAACTTCTCCATGTACATTGGAACGTTGACGCCCCTGACCTTCAGCGATAGAGTACAATAACCCTGTTGTATCTTTAAAAGTTGCTGAGGAGAGCT
CGTCAAATACCATAGGTATGCCATAATTGTTACTTAAGTAACCTTCAAGTGCATTACGTGTGGCATTCCAACCTCGAAAAAGTGTTTGATTACCTTTAGTTGGATTTCCAGCGACGGATACTGCTAAAGCTG
CTGCTGTTGATTTACCAGTTGATGACTGGCCTGTAAAACTAAAGATAATTCCTGCAAATTCGATTTCACTTTTGTACTTCAGAAAGCTTGTCACTAAGGCAGAAATTCCAAATACGACCGCTAACTCTAGA
AGAAGATGACCTTTGACTTCGTTAATATACATATTTAACCAATCTTTAAATGTACCTTTAGGCTCTAATGGATAAGCACTATCGACAATAGGATCTAAAGATGATAATTTATCATATTGTTTAGATGTATAA
ATGGTATCTATCATTACAATATAACCGTGGGGTGTTTCTATAATGCCCGAGCCATCATATAAATCAGAAGTAGGCAGTTCATCGCGCATTAACTGTAGTGCATAGCTCAAATCTTTTATATAACTTTCATTG
ATACTGTGACCATATTTAATCAAGGAAGGTAACTTGCGAGTTGTTAAAATATCAGATTCAAATGTATGTTCTTTATCCTTACCGTTAGAAATAATTATTTTTTCTGTATTTTCTAAGGCATGCCAAAATTTAG
CTTCAACAGCCATACAACTAGACATAGTCACTACTTTTTTCTCATCTCCATCTTTTTTAGGTGGGATAGTTTTATGCCAACCCATTGTATCTAAATGGTATAGGCCTAATCTAAAAATGTCATGATGACTCAT

TAGCGAACACCTCCTTTCGAAGGGTTGCTGTTATTGAGTGGATTAGGACCTGTTTTTTTATACAATAAACTACCATTTCTCTTACCAATTATAATAAAAGGAATGCTAGGTGTATGTTTTACGAAGTATGCG

AACCAACGTCCTACCTCGCGTTGAACATCTCTAGAACAGTTAATCCCTGACTTTTTAGTTAAATCCCCAATCGTAAAATCAGTTCCTTCCTGTAAATTCCATGCAATAGCTAAATCTACATTTTTTAAATTTA

TAAATTCATGACTTTTATAAGTCATAAAAATCACTCCTTACTTAATTGCTAGTAAAGTGACTAATTACTTAATCAACTTTACACTTGAATTATTGCAAGGATCAGAATTTTAAGTCATAGAGAGTTTTAATA

ACACTTTTTATCGATTTAATACATTAATGTATTAACTCAGTAAAGTATAAAAGGAGAGATAAAATGAATAATTGGAAAAATGAGTTGTTCGAAAGATTAAGAGATATTATTGAACTTGAGATTGCAATAC

ATCTAATGATCAGGAAACATGAACGTAATTCGATAGATAAATATAAGAACATTAAGGAGTTTTACCATAATCTAATACAGAAGGATATAGTTATTTATGATTATGAGACATTTCATCGATTCTATAAAACA

AGCAATATTCACAATTACTTACGGGTAAAACTAAAGCCAAAAAAAGCTTGGGAAGAAGAAAAAGTCATTCATCCAGAATCTTTTAAAAGTATCTTAGCAATACGTTTAGGTGAAACAAAATTGATGGCTG

GGACTAAATACATTGACCCCAATATTATTAAAAGCTTACGCCAAACTTATAAACAACTAAATTCTAATATTGAACAAATTTTAGATAAAATAAGTAAGAATACATTTGACATAAAATTGGAGCCAAAGTT

GATTGCTTATAAAAATTTATATGAATTATATAGTTATTTTATAGATGAAATAGATAACTATAGCAATGACTTTGAACAATTACGAGCACTTCTATATTTAGAATTAGATTACTCATTTGTACTTTATAATGA

GTTACTAAGGATGATACATTTAATCAAAAAGTATAAACTAGCTACTAGTAGCAAGATAATTGAAAAAGAAATAGGCGAAATTAGTATGAAGTTAGCAAATATAGAATTTCCGTGGCTAAGATTAGTAGTT

CTAAGAGCTTTTATTGATAATATAAAAAAATATCATAATATAAATTCATTGATTAACGATATTAGTCATATTACTAATTATATATTATTCGAAATCGAACAGTGGGTTAAACGAGCAGAAATTGAAGAAGG

AATTAGAGATTTAATGAATGTGAAATATGGTAGGATCCCTTATCTAGATGCAATTATAGAAAACAGATATAAAAGAAATAGTATTAAAGAATATTTTATCTTACTCAAACATGAATTTGAAATTAGATGGT

TAAGAGGTCGAGACTTTAATGATCAAGATAGGAATAAACTTCATAGAGCTATTAAAGATATATCTCTTAATCTTATTGGATACAATAACTAACTTAAACACATTATCTACAAGTAAAAACTTGTGAAAAG

AACATGTGTTCTTAATTGGTGTATAATTAGTTTAAATTAATCTTGGGGTGATGTAATGAATGAGAGTAATCAAATTATATTCGATCATATAACAAAGGCTATTGAAAATATGAAAAATAAAGGCTGGGAA

GCAAGGGATGGGCAAGAAACTTTAATGTATGATGTATTTGATGCTTATGATTCAAGAGAAAATTTAATAGTAGAAGCGCAAGTAGGTATAGGAAAATCATTTGGATATTTAATTCCTGGTATATTGATTTC

AAAAAATACTAAAAAGCCTTTAATTGTAACGACTTCCTCAATTCAATTAACTGAGCAACTAGTCAATGATATACAGAAAGTTGAAGAAATTTTACATATATCTGTTGATTGTATAGTAGGTAAAGGTGTAA

CTAACTATCCTTGTTTTAAAGAAATTCACCGTAAAAATTTAAGTAAATTAAATTTAATGGAAGCTTTAGATATAGCAAATAAAGGTCTAACAAAACAAACTGTACGTGAGACTAATTTAAAATGGAATCA

AATATCTACCAATAATTGTATTATGAGTAAATGTCATTATAAAAATGAATGCGCTTATTTTAAAATGAGAAATAAGTTAAAAGAGGGAAATAGATATATTAGACTCAATGAATATAAGCCGAAAGTCATA

ATCGTTAATCAAGATATGTTAATGATGAATTTTAAAAAGTTAACCTTTGGAAAAGAGAGTTTAATCTATGATGATCCTTGTATGCTAATCGTAGATGAAGTTCACAACTTAGAAGAGAAACAACGTGCTAA

CATGACTAAAACTATTAACTCTAAAACTGTGATAAATAAAATAAAAGAAGGCGCAAACCGAGTTGGTAGTAGATCTCGGTATTTAAAAAAATATAAAAATGATTGAAGATTGGTTTAATCTTCAAAAGGAT

AGTGCCAAAAGTGAGATATACAAAAATGGTAATTGTTTATCTACTGGACGAGTTGATATAAAACCTGTTAGTCATCATCAAATGGCCAAATTAATTAGTATGACAAAGGAAATAGTCGAAGAATTCGATG

TTAATAATATTGTTGTATTTAAACAATCTTCGTTAGTTAGTAATGAACAATTAGAAATAGCTAATAATCTGTTAACATTATTTAAAAATCTTCAAAATAACAGCGACAAATATATATTTTGGACTGAAATAA

CTAAGCAAGACCAAATTGATATTTCATTTTGTCCCAATAATATTGCTGAAACACTTAGAAAAACAGTTTTTAGTACAAGTTACCCAGTTGTATGTCTTTCTGCAACAATTACTAATAAAACTAATAATGAA

AATAGCTATGAGTACATTAAAGAAATAATTGGTTTTAAAGGCTATGAGGAAGATATTAAGTATAATGATTTTCCGTACAAGGAAAGCAGGCTCTACATACCTCCAAACTTACCAAAATTTGATAAACGTG

ATGTTAAATATTATGAAGAAATAGGTAAACATATTTTTGAATTGGCAAGCCAAAATAAGGGAGGCACTTTGATATTGTTTACTGCTAAAGATGACATTGACGGTGTATATAATGATTTATCAAAAAGGAA

ATTTAATAAAACTATCTATGTAGATGATGGTAGTAAAAGTCAAAATGAAATTATTGAATCATTTAAAAAAACTAAAGGTGTAATATTGGGTACGGGAGTATTTTGGGAAGGAATAGACTTAAAAAATGAA

TTATTAACATTATTAGTTATTGTCAGATTACCTTTTCCTACGATTGATCCAATTACAAAATATAAGATTACTAAATTAAATGATAGTAATGAAGCAGTTATAGTTCCTGAAATGATAATTAAACTGAAACAA

GGTGTAGGAAGATTAGTAAGAACAAAGCAGGATAAAGGTTTATTAGTGTTATTAGATTCAAGAATGAATAAACCCATTTATAAGCATAAAGAAGCAGTATTAGATGCATTACCAATTAAAAATATGATTC

ATAAAGATGAAGTCAAAGATTTCCTTAATAACATTTAAAGTAAGTAGTAATAGTTATCCAATATTTTCTAGTTGATTCTCTTATAATATGTCCAATTAAGTGTAGACGATTCAAGATGATATTGAGGAAAG

GATGAGAATGTGTCTTTCAGGTAGCTCATTTGATGATAACCTTAAACATAATATTTACTTTCATCCTGCTGAAAAAAGAGGTTACAGTTTCAAGCCGATAGAATACATTGGGCTATATAAAGACAAAGCAA

TAAAAGCTATCGGAAAAGTAGACAAAGTAATAGTGACGGAAATAAATGAGAGCTCATTATCCTTGAAGACAGTTTATCCTGTAGGTACTGAGTTATCGATTGATGAGTATGAAATAGTTAAAAATAAAAT

AATGGTGCTTGGGAAAGAAAAGTGGACTAATCTATTGAACGAACCCCATTATTATTACTTAATTGAAGATTTTATAGAGACAGACTACAAGAAAACATCTAAAGGTGGATTGATGGGAGTTAAGTATTTT

AATGTGAATGAAATTTTGAATAGGGATTGCTTAACCACCGAACAAATCGCTAAAGAATTATGTAATAAAGATTGGGAATGATAAATAGTGAATGAAGCTATAATGCAATAATGTATGTATAACATAATCA

AGTAATGAATTTAATGATAAGAGAGTATGGAGTAAAGAATTGAATTAGTTTGCATGGTCTATGTATCTTAAAGGTGTTGAAGGAGATACACCGAAGTATGCAGTACCAATTCTTGAAACAGACTTTAGTG

ATTTACCATCTACACATACGACAGTCGGATCACTTGATCGGTTTCGAGATAAGACAATGAGCATTAACAGAAGCGGGAGTAACTGTTGAATTCCATTTATATCCAGTTAGATACTAGAGTTTGAAGTGATA

AATCTTGATTCGGATGCTGAAATAAGTAAAAGAGCAGTTACTGAATATAATATAAGTATAAATATGCCACTAGATATTTTAAAGGGTATTTATTCACTTTTATAAAATCATCTGCTAAGATAGTATGGAAG

ATTTTTATAGGAGCTGATTATAATGCGACATATAAATATGTCATACATCATACGCTATAGTACTCTAACTCAAAGATAAGAAGTACTTAGTTATGAATAGCATTCATTTCACAAAGTACTTCAACAAAAAT

GGAGGAATATGAAATGAATAAAATAGAAGTGTATAAGTTTGTTAAAGTAAAGCAGTTAGTATATCAATTGATTAAGTTATATCGTACAAACGATATGAATTCCCATAAAACACAAAAAGATTTTTTACTA

AATGAAATTAATGATATCTTTAAAGAAAAAGATATTGATATCTCGGACTTTATTACATCGATTGACGATGTAAAATTAACTAAGAAAAAAGCAGAACATCTTTTAAATGAATTAAAAGTGTACATCCAAG

ATTTTGAAATACCTTCATCAAGTCAACTGGAGAAAATTTTTCGTAAAGTAAAAAAATTAAAGAGACCAGATATAAATTTAATTGATACAAAAGAAATTTCATATTTAGGATGGAATGATAATTCTTCTAAC

CGAAAATATATCGTTTATAAAAATTTAGATGATAAATTCGAAGGTATATATGGCGAAATTTCACCAAATAAAGTAAAAGGATTCTGTAAAATTTGTAATCAGGAATCTGATACATCACTCTTTCTCAATAA

AACTAAACATAATAAGAGTAGTGGAACATATACTAAAAAAGGAGATTACATTTGTTATGACAGTTTTAAATGTAATCAGAACCTAGATGATATAAATAATCTTTACGAATTTATTGTTAAAATAAAATAG

ATCCAAACAGCCCTGATCTTTAGAACTAATGATCAGGGTTGTTATATTTTGTACCTCACTCTTAATCGATACGCTGAAAGTCCCTATAAATAGAACGTACGTTCCGTTTGTAGTATAATGACAAAAATATTA

AATTAGAGGATTAAAAATGGGATTATAATTTTTTAATGTACCAAACTGAAAAAGGCTAAATAAAGATAAATATAAGATTAGAAAAAACTGTATGGATTAGGCAATATTTATATATAATGAAATGAATTAA

CAGAAAATTGAATTATCCGGAAAAACCGTACTGTTCGATTGGAAGGGAATAGAGAAGGTGAAAAGAATTAACTTCAATTATCATCTTATTTCTGAAAAAAGATATGAAAAACGGACGCAAGACACTTTTG

GACTGACCCCAATGAGTGGGAATGATATAAAAACACTTTCGTTGAATTCATATTAAAATGAATCATGCGAAGGTGTTTTTTCTATGAAAAGAGTTTCTTATTCAGTAGAAACAAAGTATAAAACTGTTGAA

ATGAAAGCAGCAGGATTTTCAACAAAAGAAATTATGGAAGAATTAAATATTAGAAATAGAACACAAGTGGAAACTTGGTGGCGATGGTATCGAAATGGGGAAAGTTATAGATTTTCACAACACGTTGGT

AAACAATATACCTACGGTAAAGGATTAGAAGAGCTGTCAAAAGTAGAACAATTAAAATTAGAAAATAAGAGAAAAGATATAGAATTGGATATTTTAAAAAAGTACAAGGCATTGGAAAGGAAGTGGTA

CCAACAGTAGTCATAGATTTAGTAGATCAATTAAAAGGAAACTATTCAATCAAATTGATACTAGAAGTATTAAACATACCTAAATCAACATATTACCGATGGAAAAACAAAACCTATAAAAATGATACTG

TAACACAAAAAGTTATTGAATTATGTGAAGCTAACCACTATACCTACGGTTATCGTAAGATTACAGCACTGATTAATCAATGTTATACATCATCAATTAATCATAAGAGCGTACAGAGAATGATGCAGAA

GCATCATTTGAACCGCCGAGTTAGACCTAAAAAGACGACAAGGATAGGTAAACCGTATTATAAAACAGACAATTTATTACAAAGACAATTTATTACAAAGACAATTTAAAGCGAGTTGTCCCATGGAAGT

ATTAACAACCGATATTACTTATTTACCATTTGGTCATTCTATGTTGTATTTATCTTCGATAATGGATATTTATAACGGAGAAATTGTGGCGTATAAAATAGATAATAAACAAGACCAACGTTTAGTTAATGA

TACATTAAATCAAATCGATATACCTGAGGGGTGTATATTACATAGTGATCAAGGCAGCGTTTATACATCTTATGCTTATTATCAATTATGCGAAGAAAAAAGCATTATCAGAAGTATGTCCCGAAAGGGA

ACACCTGCCGATAACGCCCCGATAGAAAGTTTCCATTCCTTGCTAAAGTCTGAAACTTTTTACATCAATAATCAGCTTAATAGCTCTAATCATATTGTAATAGATATTGTCGAAAAATACATTAAAAACTAT

AATAATAATCGAATTCAACAAAAGCTAGGCTACTTATCCCCTGTAAAATACAGAGAATTAGCAGCCTAGAACATAGTGTTTTTATTAAGTTCCCATTTTAAGGGTTCAGTGCCCATAATGTCATGGCTTTTT

ATATTGAAAAAACATGCGATAATTTGTTCCCAAATGTCCTTATAGTTGTACTAATATCTGTAATAGAACACTAGAAACTTGGTTTAACCTTTCCTTTTCGTAAATAAATGTAAAGATATACCATAAGGATCT

CTTACATAACCATAACCTTCAGTATAGAACTCTGGGCTGAATGCTTTTAATACTTCACTGCCTTTTTCTATTAACTGGTCATACACACGCTTAGTTTCTTCTACATGATCAAAAGTAATACAAAGCGATATA

TTATTACCTTGGGTTATAGGTAAACCTTCGGTATCATCCGCAATCATAATTTTTATATCTCCAAATTGAAGTACACATTGATCAATTTTATTTAAATCTTTTTTATCGATATTAAGTTGCTTATCTGTCGGTCT

ATCCTTGATACGTTGGATATACAGTGTTTTAGCGCCAAACAGCTCTTCATACAACCTTTTCAAACCATCTGCATTTTCTATAATTAAAAAATAGGGACTTACTTGAAAATTCATAGTTTTTCCTCCTTAAATT

TGTTATAAATAAGTATATACTGTATTAGTGTCACCTATTGATACTAAAAGGGGAATAATAATGAAAAAATCTGTTAGATTATATCATATGATTGAATACTGTAATGAAAAAAGGAATTTCAAGTTAAATGA

TTTAATGTCAGAATTTAATATTTCTCGTAGTACTGCTTTAAGGGATATAAAGGAAATTGAAGCATTAGGAGTGCCTTTATATAGTAGTACAGGGAAAAATGGCGGTTATATGACCATAGGTAATCGAAACC

AAACGAAAATAGCAATCACAGACGAAGAATTGAAAGCTTTAGTATTTACACTTTCAAGTATCTCCAATGTTAGTAAGTTACCTTTTCAAACAGAATATCAAGAAATATTAAAAAAATTATATAATAATTCG

AATAAAAAAGAATTGATAAACCAGTATAATGAGATATTTCAATATTTTAATGAAGATACATATCAGTTCAAAAGTTATAAGATATTTAATGAAATCATTAGATTAACAATTGGTAGTAAGTCATTTAAAAT

CTGTTATTCACAAAACTACTTGAAAGAACAATATAAAGGTATTGGTATCTTATATAAAAATCAACAATGGTACTTCGTTGTTGTTAATATAGCATCAAAATTAGTAAAGTTGTTAAACATTTCTAAAATAA

AAGAAGTATATGAAATGGGAGAAACTAAAGAATGTAATGAAATAACTATGCAGAATTTTCAACAGTTTATGGTTGAAAACGAAAATACGATTGATATACAAATTAGAAGTAATATTATTGGATTAAATAT

CTTGAAAGGATACTTATGGAGTGACTATATGATTGAAAATATTAACGAAGAGACATATTTGTTTAAATCAAAAGTGAACATGCAAGATATAAATTTTATAGCTAAATTAATAGTCACATGTGGTGCCAAT

GTCAAAGTAGAATTTCCTATTAGTTTGAAAAAAGCTATTAAAACTGAACTAACCAAAATAATAAACTTATACTAATGGTAATTTAATTATGATTGCAATAACAAATAAAAGATAATCTTAAACATATTAAA

TTGATCCGTAGTATAAAAACCGCATCATTAACCGATACGCAGAAGCGTACCACAAGTGATGTGGTTTTTTAGTTTTATATAAGTATTTTAAACTTATAATACTTTCTGATATGTATGATAGTAGTTATATTT

GAGTGAAGTTTATCATAAGTTACAATGATCAATTTATTTTTTAAAAATTAATCAAAAAAATAATAAATTCAATTTGTATACAATAACTAAAAAACAAAAAATAATATATGCATATAATTGGGTTTTGTAGT

ATTATATTATTATATTAAATCAGTTTCATCTATAGATAATTCATATAGGAGAGTGATGGGTTGAACAAAGTTAAAGTAAAGTCTATTAAAAATGAAAAATCGGGATGGTTTTTTACAACAAATAAAGAAG

AATTAATTTTTAATGCGCAGAAAATTTTTAATTTTAGTTTCCCTGCTATCCCTTTTACAGACTTAAAATTGCACAAATATAAAGAATTAGAGGAATCAAATATTTATTATCTGTCAATAGAACATCTGTTTG

ATAGAGAAATTAGTAGAAAACAAGAATGGAAAATTTCTAGTAATTTAGAATTTGATAACAATTTATATGGGGAATATAAAATAGAGACAAATGACCTTTATAACACGATATTAAAAATTGAATATGAAA

AAATGAGTATCATTGTATCACCAAAATTAATAACGCCAATGTTACAATCAGAAAATTTTAATGAATATTTAATTTTAGATGTTGATGATAATGAACATGATTTTGATAAAGACCTAGTCATTAAAAAGAGA

CCATTACCAAAATTATGGCACTTTCATACTTATGTTATTAAATCAACTCAACTTAAAACTGACAAATGGGATTTAAGAGAAGAAATTAACAAATTAGACATAATTTCAGAAAATTTAGATATTTGGGATGT

TTTTATAAGGTATAAAATTAATAATCAAATCTCAGATAAATACTTATTGGTAAATGATTCTTTTGATAAAAAAAATATTCACTAATAATGGAGTGAATTTTATATTTTATAAAACCAAAAGTAATAGTTTAGC

TATTGAAGTTCGAAAAAATAATTACTTAATTCAGGGAAAATTAGATGATATTACAGAAACAAAAGAAAAATTAATTTTTAAATACAAAAATTTCACATTGAATAAATTTATTATGAAAGAAAAAACAAAT

AAAATGGTACCGCAAATTTTTGAATTTTCTTTGAAAGATAATAGTATAAATAAAAATATAATATTGGAAAAACTTCCAGATTTAGATGCTGATTATGATTTATTTTTAGAAAATGATTCCGTAGAAAATAA

ATTAAAAAATATAAATACTAAAATTAATTTTTCATCTGAAAATAAAGGAATTGAAATATTTGAGAATAATAAAAATGAGTTAGTATTAAAAGTTAAGGAAAGAACTAGTAATAAAATACGTATCGCTGTA

TTAGGATCTTGTTATTCAAGGAATATGTTTAATTCTGGTGAATATTTTAATCCAAATTATAAAGACATATATGATGTTGTTTTTACACAATTTCATAGTTCAGTCATTTCAGCTGTATCATCTCCTGTAAATT

TGAATTATAGTAGTGAAAAATATATAAAAGATTCCCAATTACAGTATGTAAAGAGAGACTTTCAAAAAAATATTTTTAATGAGTTAAGTAGAGTAAATTGTCAGTATATAATAATTGACTTTTTTGTAGAT

GCAACGCAACCGCTTATAAAAACTAATGATAATAATTATGTGTCTGGAAATTTACACCTTAGAGAAACAAAATTATTAAAGTGTTGGAAGGATATAGATTTTATAAGACAAGTAGAAAATGAAGAATATT

TTATTAAATGGAAAGAAGATTTGAATACATATATGGATAACATCTCAAAGATAGTTCCATTAGAAAAAATAATCTTAGTTAAAGGTAGGTCAGCTTATGCATATAAAGATAAATTAGGTAACAAACATAA

TGTTAAAGACCCTAAATTATCAATTCAACAAAATTATTTTTGGGAGCGAATGAACAATCATTTCTTAAAATCTTATCCTAGAGTAAAGGTTATAGATATGACAGAAGACTTTTGGATTGCTGATTTCAAAC

ATCCTTTTGGCGCTTCTTTAGTTCATTATTCAAGTGAATTTTATAAAGAAGAATTTAATAGGCTCAACAAAATTATTTTAAAAGATCTTCTTGTTAATAAGTAGAAAGGTTTTAATAAAATGTTTTCTGTAAT

TATACCTGTACATAATAGTTGGAAAACTATTGATAGAACGATAAATAAAACTTTAAATTCATTTATAGACATAAATGATGAAATAATTTTAGTTAATGATAATAGTAATGAGTGTACTATACAAAAACTAA

ATTATTATTCTAAATATCCTAATATTCGAGTTATAAATAATTTGGGAGTAGGAGTAAGTGATGCAAGAAATAGTGGGATAGAAATAATAAATAAAAAAAATGAATTTGTGACATTCATTGACGATTCTGA
TTGGGTGAGTCAATCCTTTTTTTGTGAAGCTAAAAATTTTTTTCAGAAAGAGAAAGAGATTGATATAGTTTTTACACCTATAAAAATTTTTAAAGAAGGAAAAATTTATGAACATACGATGAATGATAAAT
TCAAATTCAATGGGAATATTATAAATATATTTAAAAATTATCAATTTGTTCAATATCACATAGGTGGGGTAATATTTAGAAACACATTATTAAAAAATGAAAAGTATCGCTTTGATAATACTATAGATTAT
TGGGAGGATGCAAAATTAATAAATACAATTTTATTAAACAAGCAAAAATATGGGATGTTAAAAAATTCAATTTATTTTTATGATAGAAATGATCCTAACTCTTTAAGTAACAAAGCTTGGTTGAATAAAA
ATAGGTACTTAAATCATTTGAAAGATAATTATATGTATTTAATTAACCAATCTAAATACAAATATGGTTATGTAATAAATTATGTTCAATATTTAATTACAAAACACTATTTAAACTATTTGTATTACGATA
ATCAAAACAAAATGTTGGAACACAATGATGAAGAATTTGACGAGTTTAAAAAAGAATCACAATTAGTATTTAAAAATATAGACAAGAAGATCATCGATTTACAACCAGTTCCAGAAGTGTATAAAAACT
ATTTATATCAATTAAAAAATTCAACTTATTTTTGGAAAAAAAATATAAATCATATTCATGTTTATATACATTCGATTTCAATTATAAAAAGAAATATTGTTTTTTCGTTCTCCGATAATAGTTTTTTTATATC
AGAAGATTCAAATATATATATCAAATTTAAGGGAAAAAAATTTATAAACCTAAATTAAAATTAGTGAGAAATCATTTGATTTTTGGAAAGAAAGTGAATGATTTTAGTAGACAAATATTTAATGTAACTA
TTCCGATAAAGTTCTTTTTTCATGATGCTATTTTTGTAATTGAAGATAAAAATAATAAAATAATTTTAAATATAAAATCGAAATCAATTGTTAAGAGAATATTAAAAAGAAAAAATAAATTGAGGGATTAT
TAATGAATAAAAAATATGAAGTTACTGTAATTATACCTGTGTATAATGCTGAGGAGTACATTATTAATACACTAAACTCAGTAGAAAATCAAAATTTTAATAAAAATGTTGAAGTTTTGATAATTAATGAT
GGTTCGAAAGACAATTCCATTAATATAATTAAAAGTTTTATTTCAAATTCAAGCAATGGCTATATAGACTATAAGCTATATGACGATGGGATAAATAAAGGGCAGGGAGCACGAAGAAATTTTGGTATAG
AAATTGCTAAAGGAGAATCGATATTATTCTTAGATTCTGATGATTACTTAGTTGAGAATGCTTTGAAAATAGCTTATTGGAGATTGATGGCTGTTCCCGATAATAGTTTTGCAATTTTTGAGTGGGCATATT
ATTACCCTGAAACAGGTGAAACCAAATATATAAATAAAGAACAATATAATAAAAAAAATGCATTGTATAGGGAAACTTGTGAGTTATTATTAGCATGTACTACATATTTTTCGGTTAATAAACTTTATAAA
AAAGAGTTTTTAATAGATCATGATATCAGATTTGGAGAAGGGTATTTATATGAAGACTTCGAATTTTATGTAAAAAGTGTATTAAGGTCAGTTCGAGTACCAGTGATTTCTAACATATTATATAAAGTCAG
AGTTCATGAGAACTCTTCTACAAAATCTGATGGAAAAACTTTAAAGCATAGAGATTCATTTTTAATTGCTATAGAAAGAGCATTGAAAAATTTATATGAAACAGGCTATCGCCATCATTTTACGCCATATC
ATGTAGTAAAATATTTTCTACATCGATGTCTATTGTATTCTGAAAACCGTTTACCTAAAAGAAAAAATATTAGAAAAAAAATTTATATATGATACTATGCAAATTCTAAATGATTATCTACATATGGTTACTG
TACCTAATCATGTAATTCCACTTTATGATTATGCTTTTAATAAACACTTGATAAGAGATTTAAAGGTGAAAAAAATGCAAAAAATATTTCGCTTACATAGGACTAATTTATTGAATCATTATGCAAACAGA
AAATCTTTGAAAGCTGATAAAAAGTATAATTTAAAGCAGTCAGTAAAAAATAATTATTATTTAGAACCATTATTATTTTATACTAGAAGAAAAGTTCATAAACACAGAAAAAAACAAAGAGGTAGACAGC
TAAAGAAATATTTAGATTTAAATATAAATAATGAAACTATTTTAATGTTAGGATTTGATTATCAATATAGAGGAAATTCCAAATATCTTTTTAATTATTTACAACAAAATTTTTCACCAAATCAATTAAAAA
TAGTATCTTTTGATAAAAACATAAATGAAGAGTATAGAATAGCTCCAAGAAGCGATGAATTTTATAAATACTTATATACTAGTAAAGTAATAATTGGTGAATCGTGGATTCCATTAGCATTCAAAAAGCG
AGAGGCACAGGTTTGGATTCAATTATGGCATGGCACTCCTTTTAAAAGGATGCTTTTTGATTCAAATGAATTTAAAATGCTTTCCTTAAACCCTAGCCATAAAACAAATAAAAAGTCTGATATTGATCGTT
GGGATTATTTATTATCAGATTCTAGTAAAGCTGTAGATAAATTCATTTCCTCTTATGATATAGACCGGAAAAAAATTATTAATTTTGGGTATCCAAGAAATGAATGGTTAGTAAAAAATTTAAACAATAAA
GAATTAATAAGAAACTTTAAGTTGAAAAATAATATTCCTTTAGATAAAAAAATAATATTATACGCACCAACATGGAGAGATTATAATTATCTTACTCCTGAAAGTAGAAAAGACACTAGCTATATAGCTG
ATTTTAATAAGTTATTAAAGAATTTAGATGATGAATACATAATCATAAATAAAGCGCATCCAATGGATAAACAACCGAGCTGGAATAATGGTATAAGAAATGTATTAACAGTTAATAATAATGTAGATAG
CCAAGAATTAATTTTGATTTCCGATATAATAGTAACTGATTTCTCTTCAATAATTTTTGATGCAGTACATATAAATAAACCATTTTATTTATTAATAAAGGATTTCAATAAATATATAAATACTAGGGGTGT
TTACATGGATATGTATAACTCTCTTTTACCTCTAGTATCTAATAGTGAAATCGCATTAGCTAATAATATAAATAAAAATAAATTTAATGACTTCAACATACCAGAATCATATAAAAATTCTGAAATAAGAA
ATGCTAATGTTAATATTGAAGCTATAATAAAAGAAAGTTTTATTAATTAAATAAAAATTCAATAAATATATAAATAATTTAAAAAATAGAATTATTGTAATTATAGCAATAATTCTATTTTTAATCTCTAAA
GAGGTATATTGATTTAACAAGTAACTGGTAACATTTGAACGCGTGTAATTTTTTACTATTGATACACTAAAATGAATATATGAGTTATTTTTTATGCTACAAAATATATTCAGATTTCAATAATGACATAAA

ATAGACATCTTTATATTTACCTTTAGTGTAGAATTGCTCTTTGAGTAATCCTTCAGTTTTAAATCCTTGTGACTCGTATATATGAATACTTTTTTGTTATCTGCATCAACATATAGATAGATCTTGTGCATGTT
TAAAATATCGAATGCATAAATTATCGCTTTTTTGAATGTGAATTTTGCATAACCTTTACCACTGAACTCAGGTTTAATAATTATTTGTATTTCACAATTACAATGGATGTAATTAATTTCTACTAATTCAACA
ATACCTACGACTTGATTTTCATCTTCAACAATAAAACGTCTCTTCGATTCATCTAATAAATGCTTATCAAATAAATGTCGAAGTTCCATTAAGGATTCATAAGGCTCTTCAAACCAATAAGACATAATAGA
ATATTCATTATTTAATTCATGAACAAAAAGTAAATCATTATACTCTAATGTTCTTAGTTTCATAATCCCATTCCCAAAATTTTCTTATATATTTGCTTTATAAATATAAATAACGAATAAGTCATCATTCACT
GTGAATACTCTATTTTAACAATTCACTACATACTAATTCTCATTTTCTTATTATTTTCTAATATCTCTGATTTATTACTCTTACTATGAAACATATAAAACTGTCACATTTGTTTGTATTAAAAAAATATATCA
ATAGTAACAATAAAAAAATAAATAATAGAGCATCCCTCACCGCAAAGTGAAGGATGCTCTAGTTTTATTGTAAGTAGTATGATATTCATTTTGATAGGTATTGGTTAATAATTGCCTGTAAATCATTGTTAA
GTACCGTTGTTATAGAGTCATCATTCATAAAATCATCATAGATCTTATCAAGTATTTCTTCATCTTCAATTGCTGTGAAGTGATGTACTAGGCCTTTTAAGATTGAAGTATAATTTTGAGATGCTAAGTAAC
TAGCAACCGTTCGTCAGTCTGACTGTATGGTTTTATAATCCTCCTTTTATTTCAATGTCCATTTTTGACGCGCTTTAGGATTGAGTGGATGCATGATTTCATTTGTTGCTGGGTTTATAAGCTTTTTAATTTTC
TTTTTATGAGGCTTCAACATTTCCATTACTTGTTCGACACGTTCAACGACAACTGGACGCTTCGCATAAGCACCATAAGCTAGAATGACTGTGTCACTCTCACTAATTGCTTTCATTAAGTGAATGTCTGTG
TGCTCATCGTATGGCTCGTTGATATGTTTGAGATTTTCTGGACTTTTAATATTAGAGAATAGATTTACAAGATATACAGCACCGTATCGTTCTGAATTAGCTAATTGGTTGAGGATAAGAACAGTTGTGAGA
TCGAGTGATAATACACCATCTAAATGAGGATACATCGTTATCACTGTACAAGCGGGTTTCTTTTCATCCCAAGTTTTCTTGAGTAAATAGCGGTGCTGTTTGTCATTGCTAAATATGGCTTCTGTGTGTATT
GTACTTTTAATTGTATTCATATATCATCATTTCCTTTAGTATTCTTCTGGTAAAAGCATCACATAATAAAAAGCGTCTACATCATCTTCTCGGATGACATAGACTTTCTTAGGTAATGCATTTTGATTTTTTAC
ATAGTTTGTATAGTGATATTCCAATTTATATGCGGGTTGTTCTTGTTCATGTGTGATTGAGAGTATATTATCATCTTCTTGTAGTTTAAAAATGTGTAGGTAATCTGTATTAAGTTGATTGTCTCGATCTTTTA
CCATATTCCAAAGTAGGATTTGAAGGTCTAGAGATAGTTGTTCGCTAATGCCTCTTGTGATGTATCGATTGATTTTCATACTATTTTCCTCCATTTTGCTTTTCTTTCATGATGTCAATCACTTCGTTAATGAC
TGTAACAGATAGTTGAGCGACCTTGATCCAGTTCATATTATTCTCCTCCTTTTTGTAATAGATGACGTTCATCGATAATCGTATTTTTAGTATCTGTGAGGTATAGAAAGTCCATATCAAAATGATCCAGAT
AACCAATGCTGATGAGCTGGTTATTGGCGTACATTAGAAATGGATAGATACTTAGCTCATGTAGTTCATCGTTATAGTAGGTATAAGTTTCAAGTGTAAGATGCGCAAGTGGAGTATGGTCTACAAAGCG
AGTAGGAAGTATATTTTCTGCTGCTTCCTCAAGTGCTTCACATTCCCATGTTTCGTTGTTAGATAGTTGGAATAGACGAGTTATATATTGATTGAGTTCTTGAGTGGTTGTTTTCATATCATTGCCTCCTAGA
TAGTGTGATAGTGATGTAGTTGACATACATCATTGGGATAATATATATTTGATTTGTCATTTATTACGGATCCCGGTGGGAATAAGAGAAAATTCCATGTAAAAACCCGCTACAAACGTTGGTGTAACAAG
GAAATCCCGGAATCCCACTCATTTTGACGAACAATCAACTCATTATTTATAAGTATTAATGACTGTGTTGGGTCACTACTTCCTTATATATTATCTATTTATAAAGAGTAACGGGATTTTGGGATTGTGCTT
GCACAATCCTTCTGCTTCTTCGAGTAAGCAAATCCCATTCATTTCCCGTTAAAAAATCATTGTGGGATGTTCTTTAGCAATTTCAATATAAGCATCGTGTAGTTATGAAAAAAATTATGACAATGACTGTTT
CATTAGATAAGTGTTATTGAAATTGATAAAGAGAATTCTTATAAATAGTTAGAAAAAGTAATCAGAAGAAGAAAATAAAGAACTTTCTATAATAAAAAGTCTTCACTATTAAATTTTAGTGAAGACTTTTT
ACTTAAAATTTTATTATTTATAAAACTAAGCCAATAATACTTGCTGAAATAATTGATGCTAATGTTGCACCTAATAATAATCTTAGGGCAAAAGAAGCTACTTTATTTCCCTGTTTACTATCAATACCTTTA
ATTGCTCCTACCATAATTCCAACAGTACCAAAGTTAGCAAAGCTTACAAGATAAACAGATATTATACCTTGAGTCTTTGGAGATAGAGAAGAGGCAATTTTTTGAAAATCTAACATTGCTACGAACTCATT
TGTAATTAACTTTGTTGCCATAATTGATCCAGCACGTATAGCTTCACTCCATGGAATACCCATAATGAAAGCGATAGGAGCAAATATGTAACCAATCAATTTTTTAAAATCTAGACCGACTAAATCAAATA
CAATAGTAATTCCTTTCATTAAAGATATAAAAGCTAACAACATTATTGCTACAGTAATAGCAATTCTGAAGCCATCAATAGCACTTTCACCAATCATTTGAAAGAAAGTTGTTTTTTTAATATTTTTTTCTG
ATTCAGCTTCTAATTCTTTATTATCATTTTTGTAAGGATTAATGACGCTAGCAATTATCAAAGCACTGAAAATATTTAAAACAACTGCAGTAACTACATATTTAGGATCGATCATTTGCATATATGAGCCTA
GCATAGCCATACTGACAGCACTCATACCTGAAGTAGCGATAGTATATAACTTTTCTTTTGACAAGCTTGGAATAATATTTTTAACTGTTAAATAAACCTCTGGTTGACCTAACATAGAAGTAGATATTGCG
ATATAGCTTTCAAGTTGGCCCATATGAGTTATTTTATTAATTATCCAACCTATAATTTTAATTAGAAAAGGTAAAATTTTAAAATAGTTTAACATACCAATTAAAACTGAAATAAAAACTAAAGGTAGTAA

TACATTTAGAAAGAAATTATAGCCGTTTTTATTTTGTAAATCTCCAAAAACGAATTCAATACCTTCTTTACTAATATTCATTAAACCTTCAAAAAATGTACTCATTGCGGTTAATAAATTAAGACCTATTGA

TGTATTCATCATAAATAAAACAAGAACAATTTGTATTACTATCATAATCATTGGTTTTTTTATATCTATAGCTTTTCTATTAAAACTAAAAATATATGCAACTAATAAAGCAAAAACTATTCCTAAAAATGT

GAAGATTATTGACATAGTCAAGCACCTACATTTTGCTGAAAGCTACAGCTATTTTAGCTCCTAAAACAGCGTTATTTTCTACCAACTTAATATTTGTTTCTAAGCTTTTGCCATCTGTTTTTTCAACGATTGT

TTTAAGAAGGAAAGGTGTAGAGTCTTTTCCAATAATTCCATTGGCAATAGAATCTTTAACAGCTTCTTCAATTATAGTGTCAATATAATCTTTAGAAAGTTCATGTTCTTTAGGAACAGGATTTGCAATGAC

CATTCCACCATGTAAATTTAACTCCATTTTGGTCTTGAAAATTTCAGCTATTTCTGTAGGTTCCTCAACTGAACTATTTAGTTTTAAACCGCTTTCTCTAGTGAAAAAAGCAGGTAGTTCAGTTGTTTGATAT

CCAATTACCGGAACACCTTTAGTTTCTAAGTATTCAAGCGTTTTAGGCAAGTCTAAAATAGATTTTGCTCCTGCGCATATAACTGTAACATTTGTATGCGCTAGTTCTTCTAAATCTGCTGAAATATCCATA

CTAGTTTCTACGCCCTTATGAACACCACCAATTCCACCAGTGACAAAAAATGGTATCCCAGCTAATTCTGAACAAATCATAGTAGAAGCAACAGTTGTAGCCCCAATTTTTTTAGTAGCAACTATTTCTGCT

AAGTCGCGACGAGATACTTTAGCGACATTAGGACTATTTGCTAGAGTCTCCAAGTCATTTTCGTTTAAACCAATTTTTATTTTCCCATCCATTATGGCAATAGTAGCAGGTGTGGCGCCATTGTCTCGTATT

ATTTGTTCAACTTTTTTTGCCATTTCAATGTTTTGTGGGTAGGGCATACCATGAGAGATTATTGTAGATTCTAAAGCTACTATGGGACTATTGTTTTTTTTGGCTTCTTCAACTTCTGTAGAAAATTCAAGAA

ATTTTTTCATTTTTAAATTCCTCCAATTCAATTTCTAATTGTTTTTGATCTAAATTTTGTCTAACAGTGTAATTAGTTTCAACAGTTTTCTTCGAGTTTAACAAAGCTAAGTTAATAATGTCTTCATTAGTATA

ATTTTTTAGCCAACTATATATTACTGCGCTAGTAAATGAATCACCAGCACCAGTTACATCAACTACATTTTTAGATGAAATTACTGAGTGAAATGATTCTGAACTATTATCTTTGAAAATGAGTGGTTTTTAG

TCCATTTGTAATAATGACGTTAGACACACCAAGATTAATTAATTTCTCAGCAGCAATACTCATGTCATCATTACTATCAATTTTCATTTTTAAAAAAGTTTCGGTTTCATCGCGATTAATAATTAACCAATC

AATTGAATGTAGTGAATTAGGCATATGGATCATTTTAGGTGAAGATACTGGTACAATTACTAACTTAATATTGTTTTTTTCTGCGAATGAAGTGAGGTACTCAATAGATTTTTTGGGTAAATTTAAATCTAT

TACTAAACATTTAGCTTTTAATAAGATAGATGACTGCTTTGACAGAAATTCAGGTGTAATTTCCTCGTAAATATTCATATCTGCAAAACCATACTGCATATCACCTTTTTTATCTATAATAGCTGTATATGAT

CCTGTACTTGCATTTTCTATAGGTTGAATATAATTTGTATTCATAAAAGGCTCAGATAGTTTTTTAATCAAACTCCATTCGCTATCATTTCCAGCTGCTGTTAAAAATGTAACATTTTCACCAAGTCTCCCTA

AGTTTTCAGCAATATTTCTTGCTACACCTCCAATCGATTTTGTTGATTTTACTGGATTGGAAGTCATAGATTGTAGATCAGTATCACAATAAAACTTTCTATCAACATTAGCAGCTCCAATACAGATTATGG

AATCTTCCTCATTAACAATGTATGCTTTTCCTAATATATATTTCTTTTTTATTAAACCAGATATTATATTAGCTGTGGCTGGCCTTGATAAACCAATACGTTCTGCTAACTCTTGTTGAGTAATAAAAGGATT

TTTTTTAATTTCGTTTAAAATAAATTGCTCTTTATTTTTCAAAATAGCACCTCCTTTTAAAGAAACAATAGTTCACAAACAAAACATTTGTTTGTTTCATTCAAAGAGATAATAACATATATACATTTTTGTG

TAAACGTTTTCTTGAAGGGTTTTATTTTTTATTCTATTAAAAGATAATTATACTCACTTTATTTACTAAATAATAAGATACAGTGTTTGGGACAAAAATAAAGACGAAGTGCTAAGAAGCACTTCGTCAAGT

GGATGGTTATTAAATAGTTGTTTGATAATCTCATTATTCAGTTTGAGCGTAACGTAAAATTGCTTCTTATGATGCTCATCTTTTCTTATGTCAATACGATCGATGATTGTTAGATATAGTGATTTCAACTGAG

ATTTCTCTAATTTGTCTATATCTTTGAATATTGCTTGTAATACATTAGCAATCATATCTGCATCATAATGTGGAGATTCCGCTTGTTTATCTTGTTCTAGTTGATGGATTTGATTATTTATTTGATTCAATTCA

TCTTGGTAGCTAAGTATTGTTGGTTTCAATACGCTATCTAAGTCAGGGGAGTCTTCGATAGTTTTCGTTAGTGTATGCATTTTGGCTTTTATTTCTTCACATTGTGACTGTTTATAGGCAATATCATGATTCA

AAGAAGATACGTCTATTTGACTTCTTTCATTTACCTTTTCAACCAATTGCTTCAATACCTTTTTGCTTTTGATAATTTCCAATATCTGATCCATAACATATTTTTCTAGTACATCTGCTCTAACACTATTGGCT

GAACAAACTTTTGAACCTTTGTTTCTAAAATTACTACATGAGTAATATCTGATTCTTTTCTTAGTGCCATCTTTTAATGTATTGGTCGTATTACTTGCGGCCATTGCTGCACCACATTTCGGACATTTTACAA

TGCCAGTCAGTAGGTTAGTCCCTTTACCGTGAACTTGTGGTTTCTTGCGACTCTCTTGGCGTTTAAACTGTACTTTATCCCGTAAAGCTTTATCAATAATAGGCGCATGCTTACCATCTGCAATGATTGGTTC

TTCATTGAGTCCTTTTCGTTTTTTATCGCTCCAGTGTCTATACTTCGCAAACTGTATCTTTCCAATGTAAAAAGGGTTTGAGATGATGTAAGTAATGGACGAAATGCTAAAAGGTTTCGCTTTCTTAGTCACA

TAACCTTTATGATTCAATGCGTTCGCAATCTTACGATAACCATGACCTTTAGCGTATGACTCAAAAATATATTTAACAATATTCGCTTCATGTTGATTGATCATGAGCTCTTTTTTACTGTCAGGTACTTTAT

CATAGCCTAAAGGTAAATTACCTTGGTAATAACCTTCAATCGCTCGTTGTCTTTGACCGTTATAAACGTTTTCTATAATCGTATTTCTTTCAAATTCTGCGAAGCTGGCTAAAATTTGGAGCATCAATTTACC

TGTTGAGCTGGCAATTTCTATTTTTTCAGTTAGACTAAAAAATTCGACATTGATTTTATACAATTTTTCCACAATATTTAATAAGTCTGAGGTATTTCTAGCTAAACGATTGGTTTTGTAGACCATAATACAG
TCTAACTTACCGTCATTTGCATCTTTTAACATACGTTGTAATTCTGGACGTTCCATTGTTTTACCCGATATACCACGATCGGTATATTCATCAACGACCTCATAACCTTGAAATTGACAATACTCTGTAAGTT
GGTTTAATTGACCTTGAATACTGTAACCATTTTTTTGAGTTTCAGTCGATACACGGGCATATAATCCAATATGTTTCTTTTTAAGTTGTTTCATATTACTTCCATCCTTTCAGATATTATTAAGTGTGATTGTT
CAACGATATTGAGTGGACTATTTTTAAAGTAGATACCTTGTAATTGTTTAGTTTGAGTTATTGTAATGTCATTAATCAAAGGTGCTATGGTTTCTAATGTCAATTTATCTTTGATTATATGACGGATTGTTTC
GGTAATTTGTTTATGTGAATAATTAGAGTAACGTTCTTCTCTTTCAAAGCCAGCAGATAAACGTTTAAACGTTTCGATATCAATTTGATTTTGTGCCAGTTTTTCGATGAGTTGTTCTTGTGTTAAATGTGAT
TGTCTGTGTTTCATTTGTTGGTGTTTTAGAGCTTTTAGTATTGTCTTATTTAATTTGTCATGAAATGACGGATTATCACAATATGCTTTACAAGTGTTGAGGACCTCTGTTTCAATCATTTGCGCATTGATACC
TTTAAATGGACACGTATGATAGGCTTCATTCATATTCTTAGGACAAACATAGTAACGTAGGGAGTGTTTCTCTTTTTTTATCGTTAAGTTTGTTAATGTTGATTGACAGTAAGGACATTTGATTCGTCGCTTT
AGCTTATTTCTAGAATTGGATCGATTGTGTTGCTTATGAATACGACGCTCTTGTGCTTCTTCAAACATATCAACATCAATAATAGGCGGAACGATATCATTAAATGTGCCATATTTATTAATGACACGACCG
CAATAGTTAGGGTTCAAGAGAATATTTCTAACTTGATAGGGCTTACGAGGAATAAGCTTAGGATGGTTATCTAAATGTTGGGAAATCTTTTTGTAGCCTAGACCGTGTAAGTACCAGCGATAAACTGCTTT
GACTGTATAGGCTTCTTCTTCATGTAAAACAAAATGACCTTGTCTATAACGATACCCAAAAGGTGCATGAGTTGTGATTAGCTTACCTTGTTTGGCTTTTTCTCTTGTCCCATTTCGTGTCTGTTCACTGATA
TTATTAGACTCCATTTCAGCTAGACTCATGAGTATGTTTAAGCGAAAGCAATCAAACTCTTTAGACAAATCAAAATATCCATCGTTAACACTGATGATTGTGACGTGATGCTTTTTACAAATTTCAAAGAAT
TGTATGGCATTTTTCAAATTACGATGGAGCCGATTTAAGCGATAGCAACATAATACTTTACATTTTCCAGATGTAATCATTTCTACCATTTTTTGATAACCCGAACGTTTTGTATGTCGACCTGTTTTCTTAT
CATCATAAAATGTTACATTGGACCATCCATATTGCTTAGCAGTGTCTATAATGAGGGATTTCTGAGTCGCTAAGCTTTGTTGTTTGAGTGTACTTTGACGTACATAAGCAATAACTTCTTCCATGTTATACA
CCTCCAAAAAGATAATATATATTTGTGAGTAAATTCAAATAAAGGTCCAACGTGCTTCTAACACGTTGGACCGCAATGATTAATGATCATGATTTTATTTTTCAATAAAGTAATTAGTTAGTGATGTAATA
AGTTTATATTTCAAATATGAATTATTTAATAAATATTGTAAAATTTCATTAAGTTATTTATGATAAATATAAATTAAAACGTTATTTTTATTTTATAGTGAAAGGATTAATAATAATGAAACACATACATTT
ATCTGTTTTAGCTTATAGTACAGGTCTACATCCTGCCTCTTGGAGACTACCACATTCATATGTTGAAGAAGTTGGAGATATTGATTTTCAAATTAAGTTAGCGAAATTAGCCGAAAAAGGTAAACTGGATG
CATTCTTCTTAGGTGATGGTCAATATATTTCTGGAGAAGAAACAGGACATATTTCTTATTATTTTGAACCACTAACGGCGCTTGCTGCCATTTCACGTGAAACACATTCAATTGGTTTGATAGGTACCATGT
CTTCTTCTTTTTATGAACCTTATCTTGCAGCACGTATGCTTTCAAGTTTACATCAAATATCTCATGGGCGTATTGGGGCAAATATTGTAACATCTCAATTTGATTTAGAAGCACAAAACTACTCGATACAAG
CTTTACCACATCTTGAAAAACGTTATGAACGAGCAGATGAGTTTATTAATGTAATGAAAAAATTATGGGAATCATTTACTGTAGAGGCTATCGTTAATCACAAAAACTCAGGAATTGGCTTAAATCATCAG
TATATTCATCCACTTCATTATCAAGGAAAGTATTTTCAAGTTGCCGGAGCTATTAACATTCCAACACCCAAATATGGTCGACCACGTTTATTTCAAGCAGGAACTTCCATTCCAGGTCGTGATTTAGCAGTA
CGACACGTGGATGCTATTTTTTCAATTGCATGGAATTTACAAGATGGCCAACAGTTCAGACAAGACATTCATCAACGTGCCATGGAAGCAAATCGTCAACCCCCTCTTGTATTACCAGGTCTCACTGTTTA
TGTTGATGAGAATGAAGAGGATGCTTATAAACTTAAGCAACAATTAGATGATATGATTCCACTTGAAAAACGCAAGAAACAATTATCTAAAGCTATTGGTCTAGATATAAGTGAATGGAAACTAGATGAA
GTTATACCGTCCTTACCAGATTATGAAACATTATCGAAAAAGGTCGTAAAATCAGTATATGAAGCCATACAACGTGCCATTAAGACTGAGCATCTTACATTAAGAGATTTATTAGATCGTTTTGGTACTTG
GGTAGGGCACAAGACAATAGTAGGAACTCCCGAACAAGTGGCGGATGAAATGATCGAATGGTTTAAAAAAGAAGCGTGCGATGGTTTTATGTTGATGGCCCCGACGTATCCAGAATCATTTGAAAAATT
TATTCATTTAGTGATACCAATTCTTCAAGAACGTGGAGTGTTTAGAAGAGATTATGAGAGTCATCTGTTAAAAAATCATTTGGGTATAAAAAACTAAAAATAAACCGCATCATTAACCGATACGCAGAGG
CGTATCATAAGTAAAACTAAAAAATTCTGTATGAGGAGATAATAATTTGGAGGGTGTTAAATGGTGGACATTAAATCCACGTTCATTCAATATATAAGATATATCACGATAATTGCGCATATAACTTAAGT
AGTAGCTAACAGTTGAAATTAGGCCCTATCAAATTGGTTTATATCTAAAATGATTAATATAGAATGCTTCTTTTTGTCCTTATTAAATTATAAAAGTAACTTTGCAATAGAAACAGTTATTTCATAATCAAC
AGTCATTGACGTAGCTAAGTAATGATAAATAATCATAAATAAAATTACAGATATTGACAAAAAATAGTAAATATACCAATGAAGTTTCAAAAGAACAATTCCAAGAAATTGAGAATGTAAATAATAAGG

TCAAAGAATTTTATTAAGATTTGAAAGAGTATCAATCAAGAAAGATGTAGTTTTTTAATAAACTATTTGGAAAATAATTATCATAATTTAAAAACTGACAATTTGCGAGACTCATAAAATGTAATAATGGA

AATAGATGTAAAATATAATTAAGGGGTGTAATATGAAGATTAATATTTATAAATCTATTTATAATTTTCAGGAAACAAATACAAATTTTTTAGAGAATCTAGAATCTTTAAATGATGACAATTATGAACTG

CTTAATGATAAAGAACTTGTTAGTGATTCAAATGAATTAAAATTAATTAGTAAAGTTTATATACGTAAAAAAGACAAAAAACTATTAGATTGGCAATTATTAATAAAGAATGTATACCTAGATACTGAAG

AAGATGACAATTTATTTTCAGAATCCGGTCATCATTTTGATGCAATATTATTTCTCAAAGAAGATACAACATTACAAAATAATGTATATATTATACCTTTTGGACAAGCATATCATGATATAAATAATTTGA

TTGATTATGACTTCGGAATTGATTTTGCAGAAAGAGCAATCAAAAATGAAGACATAGTTAATAAAAATGTTAATTTTTTTCAACAAAACAGGCTTAAAGAGATTGTTAATTATAGAAGGAATAGTGTAGA

TTACGTTAGACCTTCAGAATCTTATATATCAGTCCAAGGACATCCACAGAATCCTCAAATTTTTGGAAAAACAATGACTTGTGGTACAAGTATTTCATTGCGTGTACCGAATAGAAAGCAGCAATTCATAG

ATAAAATTAGTGTGATAATCAAAGAAATAAACGCTATTATTAATCTTCCTCAAAAAATTAGTGAATTTCCTAGAATAGTAACTTTAAAAGACTTGAATAAAATAGAAGTATTAGATACTTTATTGCTAAAA

AAACTATCGAATTCTTCAACTACAGAAAATATATCTATAGATATATCAAGATTTTTAGAACTAAGTAATATGATACTCTTGTTAGATGATATGCTCGACGTCAATATATATATATAAATTCTTTTAAAAATA

ATACATTAGAGACATTTGATGCGACTGATCCTGAAGTTGATTACATCACTGAAATAGGAGATTACTTATTAAAATATGACGTTAATTCTATAAATGATGTCAGAATTGAAGTGATAGATAATTTGGGGCAT

TCAAATAATATGCTACTAAAAACGATACTACATGCAGAAGTTGAAATGGAAGATGGAAAGAAATATTTATTACAAAATGGGAAATGGGGTTATTTTAATAGAGAATTTTTTGACCTTTTGAATGATCATTT

GAATGAGATAGAAATTAGGTATAACACACTAACTCCCACAGGTTTAGTATTTAAAGAAGGAGAAGAAGGATATATAAAAGAAATAGTAGGAAGATTGCCAGAAGAATATTTAATGTTACATAAAAAATT

CATAAAACCAATAAATAAGAATTTTATAGTAAAAGGAAATGGAATAGAGTTGGCAGACTTATACAATATTAAAAACAAAGAGCTTTTCACGATTAAAAGAGGAATTAATACATCTTTATCTCTTTATAGT

CTAGAACAGAATATAATAGCAATTAACGCCTTAAAATATCCAGAATCATATAATTTTGAAGAATTAATAGAAGCTATTCCTGATAACTCGGAAAATATATTTAATGATATACAACGGAGTACAAATTTTA

GTATAGTATGGATTTTACCGATATCATCTATTGATAATATGCCCATAAAAGATATGGTTCATACTAGTAACGTAATAAATAAAAACTTTCAATTAACTAATTTAGGTTCAGTATTACTTAAAAATAAATTAG

TAGAGTGGTCTCTCTATTTAAAAGATCAAAGAATTAATCCAATTATTTATATGGAAACGCCAACTGAAGATAGAAATTAAACTTTTATTTTAAATCACCCTAGTTCATACCTAAAAGACTTTCACACAAAC

AAAGGAGGAAACTTAAATTCCTCCTTTCCCTTATTACTCATACTATAATTCAATTTTAACGTCTTCGTCCATTTGGGCTTCAAATTCATCTAGTAGTGCTCGTACTTCTGCAATTGATTGTGTGTTCATCAAT

TGATGGCGAAGTTCGCTAGCGCCTCTTATGCCACGCACATAGATTTTAAAGAATCTACGCAAACTCTTGAATTGTCGTATTTCATCTTTCTCATATTTGTTAAACAATGATAGATGCAATCTCAACAAATCT

AATAGTTCTTTGCTTGTGTGTTCGCGTGGTTCTTTTTCAAAAGTGAATGGATTGTGGAAAATGCCTCTACCAATCATGATGCCATCAATACCATATTTTTCTGCAAGTTCAAGTCCTGTTTTTCTATCGGGAA

TATCATCGTTAATTGTTAACAATGTGTTTGGTGCAATTTCGTCACGTAAATTTTTAATAGCTTCGATTAATTCCCAATGTGCATCTACTTTACTCATGCGTTTGATAAAAACTTAAATAATATTAATTCGGTC

ATCAGTGGCGTTAAATCTTTTATCATTTTTAGTTATAGTTGATAAATTTATATTTATAAGCATATATGGATATTTCATCAAAAATTTTTATTTATATAAATCCGAACTGCATACATATTTGTTTAAATAAGAG

GTATTATTTTTCGGGAAATTGCTGTCTGAGTTAAAAGGATTAGTTTTATAAAATGAGTTGAACTATAGCAAAAACGATTAAAATACTGATAATCCATTTTTGTATTATGTTAGGGACTTTTTTACTTAATTTT

AACCCTATTGGAGCAAATATAATACTCCCTATTATAAGGAATAAGGCGTCATATAAAGGGATATAACCTTGAATAAGTTTGATGACAAAAGCACCAATTGAAGATATAAAAGCAATTACTATACTATTAG

CGACTACAGTATTCATTGGTAATTTGAATAAAACCAATAATATAGGAATAATAATGAAGGCACCACCTGCACCTACTATACCTGAAATAATACCAATGAAAAGGCCAATGATAACTAATAAATATTTATT

AAATGAAGACTTTTCGGAACTAGGTTTCACTTTAATAAACATTAATGTTAATGCAAGTAAAGCAATAATGATATATACCGTATTTACAAATGTAGCATCAAATAAATTTGCTAGAAATGCACCTAACATAC

TCCCTATAATCATGCCGCCACCCATATAAAGAACTAATTGTGGCGAGAACTCTGTTTTTTTTCGAGCTTTTAATGAGCCACTTAATGTACTGAAAAAGACTTGGCTAGAAGTAAGACCTGATGCGATATAT

GCGCTATATGCAGGGGCTCCGAATAATGGTGGTAATAATAAAATAGCTGGATAAATAATGATAGCACCACCTACGCCTACTAGACCAGATATGAACCCACCGAATACCCCAATGAGTAACATGATAACTA

TATTAACAATATCCATTACTTACTTTTCACCAATAAGTTTACAGCTTCATTAATTAACTCTTGGGAGCTTTCTTCATCATCCGCAGCTGCTTTTACACATTCTATTAAATTCTCACTAATAATGATACCCATC

AAGCGTTGGAGTGAACTCTTTGATGCACTTATTTGTGTAATGACATCTTTACAGTCTTTTCCTTCCTCCATCATTTTAATAATTCCATTTAGTTGCCCTTGTATTCTATTAATACGATTAATCATTTTTTTATC

ATAATTCATAGTCATACCTCCACTTTTAATTGAATAAAAATATATTAATAGATAAATACAAATGTGTCAAATACCCCTAGAGGTATTTGACAAGTTCCATCCAACTGTTTAAAATACCCCTACAGGTATTTT
TAGGGAGGTTATTATGAAACAATACGGAGAAAAGTTTATCGATGAATTTAGTAAAGCAGAATTGGAAAAACTAGCCAAGCAAGGGCAATTAATTGACGTTAGAACAGAAGAGGAGTATGCATTAGGACA
TATCAATGGTTCCATACTTCATCCTGTTGATGAGATTGAGTCATTCAATAAAGAAAAAAATAAAACCTATTATGTAATCTGTAGAAGTGGTAACAGAAGTGCTAATGCTAGTAAATATTTAGCTAAACAAG
GTTATAACGTTATAAATCTTGATGGTGGTTATAAAGCTTATGAAGAAGAAAACGATAGTTATGATACACAAGAAGAATATAAAAGTATAGAAATTAAAGCAGATCGTAAACAATTTAACTATCGTGGTCT
TCAATGTCCAGGGCCAATTGTAAAAATTAGTCAAGAAATGAAGAATATTGAAGTAGGTGACCAAATTGAAGTCAAAGTCACAGACCCTGGATTCCCTAGTGACATTAAAAGTTGGGTGAAACAAACAAG
GCATACTTTAGTTAAGCTTGATGAAAATAACAATGGAATTAATGCGATTATTCAAAAAGAAAAAGCAAAAGATTTAGATATAAATTATTCTGCTAAAGGTACTACAATTGTATTATTTAGTGGAGAATTA
GACAAAGCTGTAGCAGCGTTGATTATTGCAAATGGTGCTAGAGCTGCTGGAAAAGATGTAACTATCTTCTTTACTTTTTGGGGGCTTAATGCATTAAAAAAAGTGCAAACAGTTAATGTTAAAAAGCAAG
GTATTGCAAAAATGTTTGATTTAATGTTGCCCAAAAAGAATATACGAATGCCTCTTTCCAAAATGAATATGTTTGGTTTAGGAAATATGATGATGCGCTACGTAATGAAAAAGAAAAATGTTGATTCATTA
CCAACACTTATCAATCAAGCTATTGAGCAAAATATCAAATTAATCGCTTGTACGATGAGTATGGATGTCATGGGTATTCAGAAAGAAGAACTTAGAGATGAAGTTGAGTACGGTGGTGTAGGCACTTATA
TTGGTGCTACTGAAAATGCGAATCATAATTTATTTATCTAATTAAATCTATTAATAAAAGGAGTTGTTATCATGTTTTTAAACAGTTTTACGATAATCATTTATCTCAAGCATCATATTTAGTGGGTTGTCA
ACGTACAGGAGAGGCAATAATAATAGACCCTGTTCGTGATTTATCGAAATATATAGAAGTTGCAGATTCTGAAGGTTTAACAATTACACAAGCTACAGAAACACATATTCATGCTGATTTTGCTTCAGGA
ATTCGTGATGTGGCTAAACGCTTAAATGCAAATATATATGTGTCTGGCGAAGGTGAAGATGCATTAGGGTATAAAAATATGCCATCAAAAACACAATTTGTTAAACATGGAGATATCATTCAAGTAGGCA
ATGTTAAATTAGAAGTTCTGCATACTCCAGGACACACGCCTGAAAGTATTAGCTTTTTACTCACTGATTTAGGTGGTGGTTCAAGTGTTCCGATGGGATTATTTAGTGGTGACTTTATTTTTGTTGGTGATAT
AGGTAGACCTGATTTATTAGAAAAATCTGTTCAAATAAAGGGTTCTACAGAAATTAGCGCGAAACAAATGTATGAGTCCGTTCAAAATATTAAAAATTTACCAGACTATGTTCAAATCTGGCCGGGTCAT
GGTGCTGGAAGCCCTTGTGGTAAAGCATTAGGTGCCATACCTATATCTACAATAGGTTATGAGAAAATTAATAACTGGGCATTTAATGAAATTGATGAGACTAAATTTATTGAATCATTAACATCAAATCA
ACCAGCACCACCGCATCATTTTGCACAAATGAAACAAGTTAATCAGTTTGGTATGAATTTATATCAATCATATGATGTTTATCCTAGTTTAGATAATAAGAGAGTAGCATTTGATCTTCGTAGCAAAGAGG
CCTTTCACGGTGGCCACACAAAAGGAACAATCAATATACCATACAACAAAAACTTTATTAATCAAATTGGTTGGTACTTAGATTTTGAAAAAGATATAGATTTAATTGGAGATAAATCTACTGTTGAGAA
AGCGAAACACACTTTACAATTAATTGGGTTTGATAAGGTAGCAGGCTATCGTTTGCCAAAATCAGGCATTTCAACCCAGTCCGTTCATAGCGCTGATATGACAGGTAAAGAAGAACATGTATTAGACGTA
CGTAATGATGAAGAGTGGAATAATGGACACTTAGATCAAGCAGTTAATATTCCGCATGGTAAATTATTAAATGAAAATATTCCTTTTAATAAAGAGGATAAAATATATGTACATTGTCAGTCAGGTGTTA
GAAGTTCAATTGCAGTGGGTATATTGGAAAGCAAAGGTTTTGAAAATGTGGTGAATATTAGAGAAGGCTATCAAGATTTTCCAGAATCATTAAAATAATTTAAGGATGTGGAAAAAAATGAATAAGCATTA
TCAAATTGTTATTATTGGTGGCGGTACAGCAGGTGTTACCGTAGCATCAAGACTATTAAGAAAAAATCAAAACTTAAAAGAGAAAATAGCAATTATAGATCCAGCAGACCATCATTACTATCAACCATTA
TGGACGTTGGTTGGTGCAGGGGTATCTAGTTTGAAAAGTTCTCGTAAAGATATGGAAAGTGTTATACCTGAAGGTGCTAACTGGATAAAACAGGCTGTTTCAAGTTTTCAACCTGAAAATAATAGCGTTAT
TTTAGGAGATAATACAGTCGTTTATTATGATTTTTTAGTAGTAGCTCCAGGATTACAGATTAATTGGTCTTCAATTAAAGGACTAAAAGAAAATATAGGTAAAAATGGTGTTTGCTCTAACTATTCACCTGA
CTATGTTAACGAAACTTGGAACCAAATTTCTAATTTTAAACAAGGAAATGCCATTTTTACGCATCCAAACACTCCTATAAAGTGTGGAGGTGCGCCTATGAAAATTATGTATTTAGCTGAAGATTATTTTA
GGAAACATAAAATCCGTTCTAACGCTAATGTGATATATGCAACGCCAAAAGATGCTTTATTTGACGTAGGAAAATATAATAAAGAATTAGAAAGGATTGTTGAAGAAAGAAATATAACAGTCAATTATA
ATTATAACCTTGTTGAAATCGACGGTGACAAAAAAGTGGCTACATTCGAACATATCAAAGCATACGATAGAAAAACAATAAGTTATGATATGTTACATGTAACACCACCTATGGGTCCCTTAGATGTAGT
AAAAGAAAGTACACTTTCAGATAGTGAGGGTTGGGTAGATGTTAACCCAACCACATTACAGCATAAAAGCTACTCTAATGTATTTGCACTTGGTGATGCTTCAAATGTACCTACTTCAAAAACAGGCGCA
GCTATTCGTAAGCAAGCACCTATCGTCGCTAATAATTTATTGCAAGTGATGAATAATCAAATGTTAACGCATCATTATGATGGTTATACTTCATGCCCTATTGTTACTGGATATAATAGGTTAATACTTGCA

GAGTTTGATTATAATAAAAATACTAAAGAAACAATGCCGTTTAATCAGGCCAAAGAACGTAGAAGTATGTATATATTTAAGAAAGATTTATTACCTAAAATGTATTGGTACGGCATGCTAAAAGGATTAA

TATAATAAAGTACAGAAAACAATAAATTTTTAATGAAAAATCTTTTACTATAAAAGATTAAGTATTTAAATGACGTGTCAGTGTTGTGTTTATATGTCGTGAATTTTTAGCTCTAAATAGTATAAGATTGAA

AAAGTTGTTACTGTTTTAAATGATCACGATGAAGTCATTCAATAAGAATGATTATGAAAATAGAAACAGCAGTAAGATATTTTCTAATTGAAAATCATCTCACTGCTGTTTTTTAAAGGTTTATACCTCATC

CTCTAAATTATTTAAAAATAATTAATGGTATTTGAGCACGTTTAGCGACTTTATGACTGACATTACCAATTTCCATTTCTTGCCAGATATTCAAACCACGTGTACTCAAAATGATAGCTTGGTATGTACCTC

CAATAGTAATTTCAATAACTTTGTCTGTTGAACACTAAGAGCAATTTTAATTTCATAATGTGTTGTAAACATTTTTTTTGATTGGAGTTTTTTTCTGAGTTAAACGATATCCTGATGTATTTTTAATTTTGCAC

CATTTCCAAAAGGATAAGTGACATAAGTAAAAAGGCATCATCGGGAGTTATCCTATCAGGAAAACCAAGATAATACCTAAGTAGAAAGTGTTCAATCCGTGTTAAATTGGGAAATATCATCCATAAACTT

TATTACTCATACTATAATTCAATTTTAACGTCTTCGTCCATTTGGGCTTCAAATTCATCGAGTAGTGCTCGTGCTTCTGCAATTGATTGTGTGTTCATCAATTGATGTCGAAGTTCGCTAGCGCCTCTTATGC

CACGCACATAGATTTTAAAGAATCTACGCAAGCTCTTGAATTGTCGTATTTCATCTTTTTCATATTTGTTAAACAATGATAAATGCAATCTCAATAGATCTAATAGTTCCTTGCTTGTGTGTTCGCGTGGTTC

TTTTTCAAAAGCGAATGGATTGTGGAAAATGCCTCTACCAATCATGACGCCATCAATGCCATATTTTTCTGCCAGTTCAAGTCCTGTTTTTCTATCGGGAATATCACCGTTAATTGTTAACAATGTATTTGGT

GCAATTTCGTCACGTAAATTTTTAATAGCTTCGATTAATTCCCAATGTGCATCTACTTTACTCATTTCTTTACGTGTACGAAGATGAATAGATAAATTGGCAATGTCTTGTTCGAAGACGTGCTTCAACCAA

TCTTTCCATTCATCGATTTCATAGTAGCCAAGGCGTGTTTTAACACTTACCGGAAGCCCACCTGCTTTAGTCGCTTGAATAATTTCGGCAGCAACGTCAGGTCTTAAGATTAAGCCGGAACCCTTACCCTTT

TTAGCAACATTTGCTACAGGACATCCCATATTTAAGTCTATGCCTTTAAAGCCCATTTTAGCTAATTGAATACTCGTTTCACGGAACTGTTCTGGCTTATCTCCCCATATATGAGCGACCATCGGCTGTTCAT

CTTCACTAAAAGTTAAGCGTCCGCGCACACTATGTATGCCTTCAGGGTGGCAAAAGCTTTCAGTATTTGTAAATTCAGTGAAAAACACATCCGGTCTAGCTGCTTCACTTACAACGTGTCGAAAGACGATA

TCTGTAACGTCTTCCATTGGCGCCAAAATAAAAAATGGACGTGGTAATTCACTCCAAAAATTTTCTTTCAT
